# Annotated type catalogue of the Orthalicoidea (Mollusca, Gastropoda) in the Museum für Naturkunde, Berlin

**DOI:** 10.3897/zookeys.279.4701

**Published:** 2013-03-25

**Authors:** Abraham S.H. Breure

**Affiliations:** 1 Naturalis Biodiversity Center, P.O. Box 9517, 2300 RA Leiden, the Netherlands

**Keywords:** Bothriembryontidae, Bulimulidae, Megaspiridae, Odontostomidae, Orthalicidae, Simpulopsidae, type material, correspondence

## Abstract

The type status is described of 96 taxa classified within the superfamily Orthalicoidea and present in the Mollusca collection of the Museum für Naturkunde der Humboldt-Universität zu Berlin. Lectotypes are designated for the following taxa: *Orthalicus elegans* Rolle, 1895; *Bulimus maranhonensis* Albers, 1854; *Orthalicus nobilis* Rolle, 1895; *Orthalichus tricinctus* Martens, 1893. *Orthalicus sphinx tresmariae* is introduced as new name for *Zebra sphinx turrita* Strebel, 1909, not *Zebra quagga turrita* Strebel, 1909. The following synonyms are established: *Zebra crosseifischeri* Strebel, 1909 = *Orthalicus princeps fischeri* Martens, 1893; *Orthalicus isabellinus* Martens, 1873 = *Orthalicus bensoni* (Reeve, 1849); *Zebra zoniferus naesiotes* Strebel, 1909 = *Orthalicus undatus* (Bruguière, 1789); *Porphyrobaphe (Myiorthalicus) dennisoni pallida* Strebel, 1909 = *Hemibulimus dennisoni* (Reeve, 1848); *Zebra delphinus pumilio* Strebel, 1909 = *Orthalicus delphinus* (Strebel, 1909); *Orthalicus (Laeorthalicus) reginaeformis* Strebel, 1909 = *Corona perversa* (Swainson, 1821); *Bulimus (Eurytus) corticosus* Sowerby III, 1895 = *Plekocheilus (Eurytus) stuebeli* Martens, 1885. The taxon *Bulimus (Eudioptus) psidii* Martens, 1877 is now placed within the family Sagdidae, tentatively in the genus *Platysuccinea*. Appendices are included with an index to all the types of Orthalicoidea extant (including those listed by Köhler 2007) and a partial list of letters present in the correspondence archives.

## Introduction

A steady stream of papers on type material of the superfamily Orthalicoidea in major museums has been published during recent years (Neubert and Janssen 2004, Köhler 2007, Breure 2011, 2012, Breure and Ablett 2011, 2012, Breure and Whisson 2012). For the Berlin museum only part of the extant type material was treated by Köhler (2007) due to limitations at that time. A recent visit to the Berlin museum under the SYNTHESYS programme made it possible to compile a list of all type material in that collection related to the superfamily.

The Orthalicoidea is a large and diverse superfamily distributed mainly in the Neotropics, but with an important Gondwanan element (Herbert 2007, Neubert et al. 2009, Delsaerdt 2010, Breure and Whisson 2012). The classification of the group at the family level has recently been revised by Breure and Romero (2012), who recognized in total seven families based on phylogenetic results: the Neotropical families Amphibulimidae, Bulimulidae, Megaspiridae, Odontostomidae, Orthalicidae, and Simpulopsidae; the family Bothriembryontidae has a Gondwana distribution (southern South America, South Africa, Australia, Melanesia; see also Breure and Romero 2012).

## The collection

The Berlin museum (ZMB) has a history of nearly 200 years, which has resulted in a malacological collection of ca. 118000 registered lots and many unregistered ones. Some of the malacological curators had an active role in describing new orthalicoid species, viz. E. von Martens, B. Rensch, and J. Thiele (see below). However, as may be seen from the catalogue herein, these curators have been able to acquire a substantial amount of type material described by other authors, sometimes through intermediates like the shell dealer Krantz, but more relevant through the acquisition of private collections (e.g. Dunker, Albers) and the exchange of material with other institutions.

Material has been found which was collected or studied by the following persons: Johann C. Albers (1795–1857); Heinrich Dohrn (1838–1913), who exchanged material with Doering, Pfeiffer and Stelzner, cf. Dohrn (1875: 202–203); Adolfo Doering (1848–1926), who supplied type material to Dohrn and others with manuscript names before these were published, cf. Breure and Miquel 2012; Wilhelm Dunker (1809–1885); Ermst C.L. Gruner (1786–1857), cf. Tëmkin et al. (2009); Siegfried H.F. Jaeckel (1892–1970); Eduard von Martens (1831–1904), author of the Mollusca part in ‘Biologia Centrali-Americana’, including several brief biographies on collectors in the introduction of that work (Martens 1901 [1893–1901]: vi–xii); ? Moritz (no further data found); Friedrich Paetel (1812–1888), who donated his large shell collection to ZMB; Felipe Poey y Aloy (1799–1891); Bernhard Rensch (1900–1990) and his wife Ilse Rensch-Maier (1902–1992); G? Sievers; Hermann Strebel (1834–1915), see below; A. Stübel (no further data available); Johannes Thiele (1860–1935); and Franz H. Troschel (1816–1882). Brief information on the origin of the ZMB collection and the contributions of several persons mentioned above may also be found in Richling and Glaubrecht (2008), Tëmkin et al. (2009), Glaubrecht and Zorn (2012).

The material of H. Strebel is especially important in the context of this paper, since his own collection and the material in the Hamburg museum was destroyed during the 1940–45 war. Strebel made two major contributions to the orthalicoid literature. The first one was when he published, together with G. Pfeffer, his fifth and final volume on Mexican land shells (Strebel and Pfeffer 1882). Here he treated both (in our current understanding) Bulimulidae and Orthalicidae from that region and published ten new taxa. He also gave detailed anatomical data, something that only had started to be done recently at that time. His second contribution (Strebel 1909) was the revision of all the Orthalicidae known at that time. This work was following the treatment of this group by Pilsbry in the Manual of Conchology (Pilsbry 1899, 1900); only a decade later he described eight new (sub)genera and 19 new species-level taxa. His very detailed and precise studies led him to recognize both ‘Formen’ and ‘Heteromorphen’, of which he additionally recognized 37 taxa. According to the index of Strebel (1909), the names of ‘Hetromorphen’ were sometimes not indicated as new names, but the combination of main text and index always makes it clear when these names were newly introduced by Strebel. Under ICZN Art. 10.2 and 45.6.4 these taxa have to be treated as infrasubspecific names (ICZN 1999). On one hand, the ‘Formen’ and ‘Heteromorphen‘ point to Strebel as a ‘splitter’; on the other hand, his meticulous works made him stand out ahead of his time.

## Handwritings and labels

**Figure 1.**
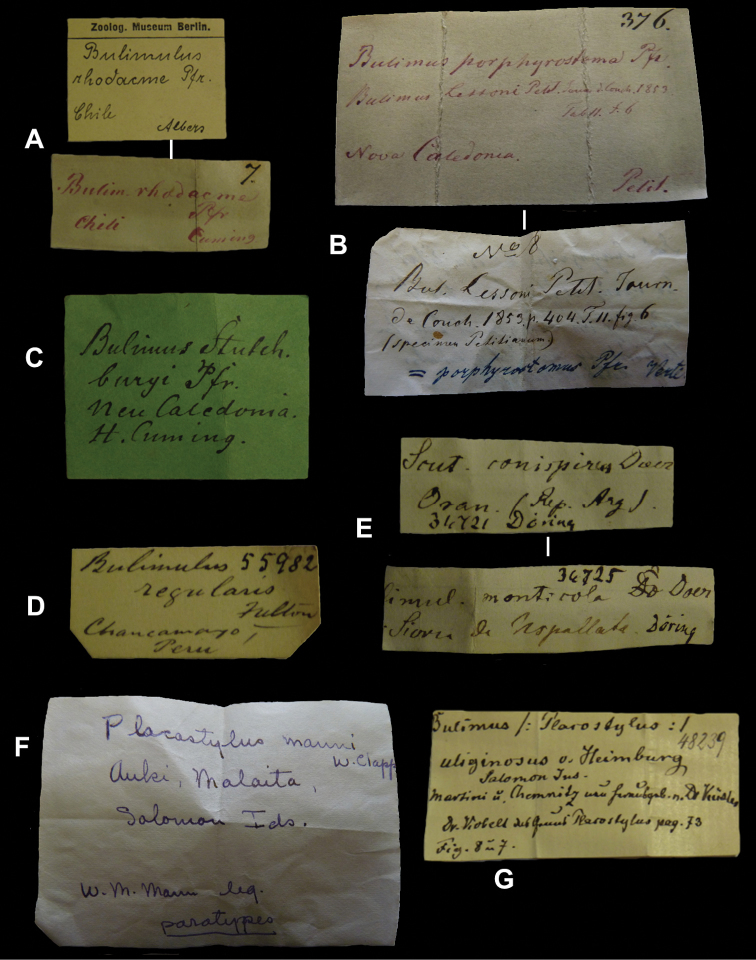
Labels with author’s handwriting. **A–B** Two different series of labels originating from Albers, one (**A**) without the original label, the other (**B**) with the original label (in this case from Petit); note that the lower line is in L. Pfeiffer’s hand **C** W. Dunker **D** H.C. Fulton **E** Two labels with partly the handwriting of A. Doering, and partly written in Martens’ hand (the numbers and the second occurrence of Doering’s name (“Döring”)). Note the two different inks on the lower label **F** W.F. Clapp **G** H. von Heimburg.

**Figure 2.**
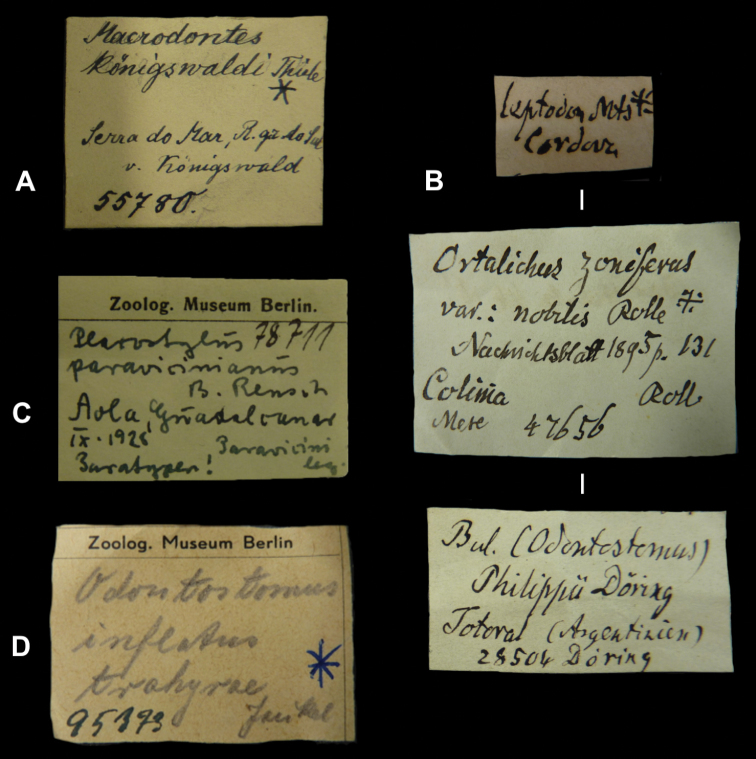
Labels with author’s handwriting. **A** J. Thiele **B** E. von Martens **C** B. Rensch **D** S.H.F. Jaeckel.

**Figure 3.**
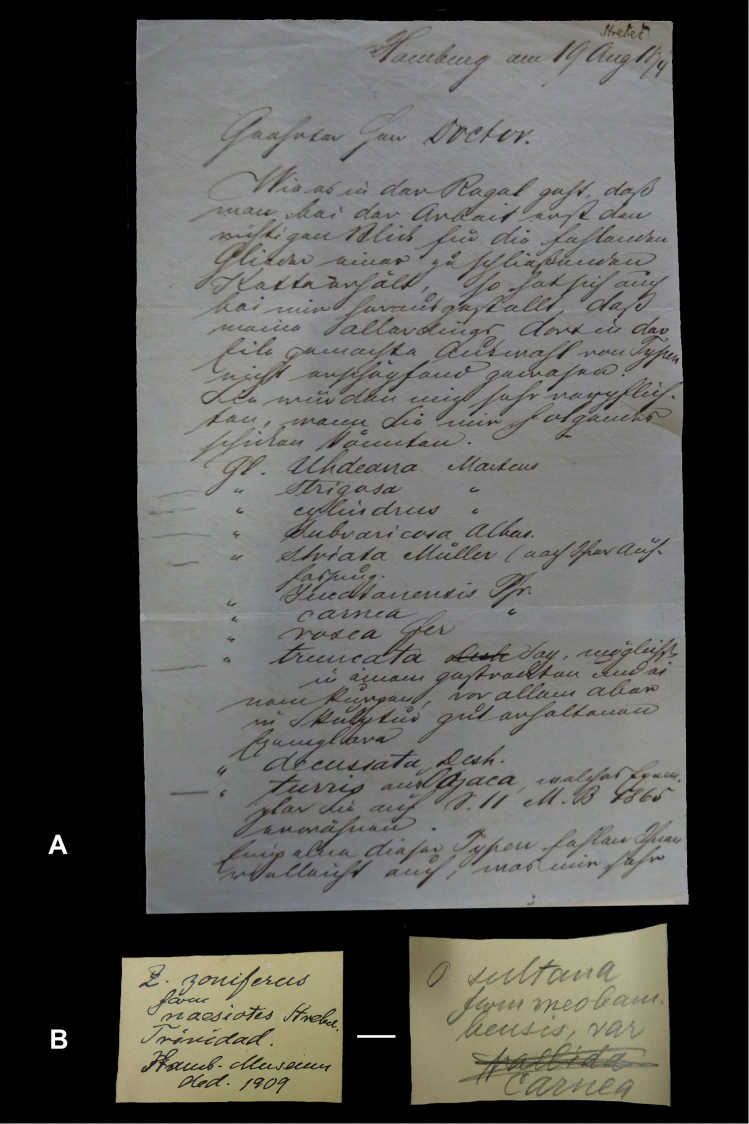
Example of a handwriting through time. **A** Excerpt from letter of H. Strebel, d.d. Hamburg, 22.v.1877 (13.5 × 22.5 cm) **B** Labels in Strebel’s handwriting, ca. 1908.

**Figure 4.**
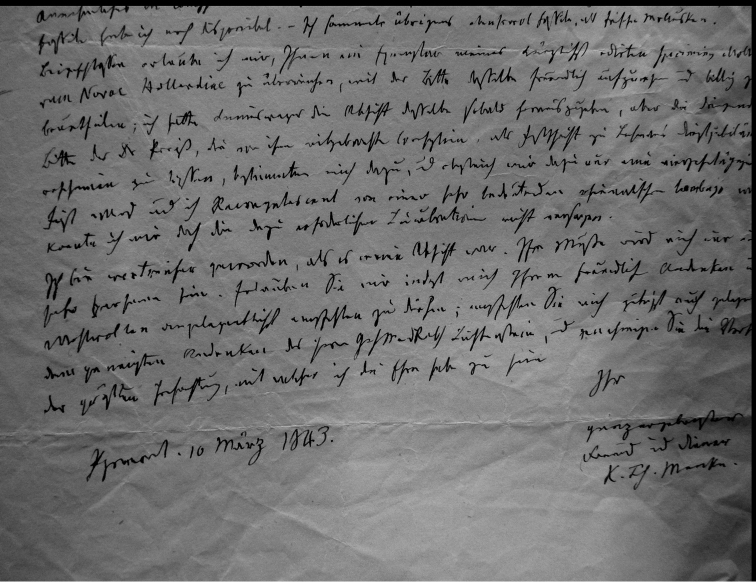
Handwriting of K.Th. Menke. Excerpt from letter d.d. Pyrmont, 10.iii.1843 (19.5 × 25 cm).

**Figure 5.**
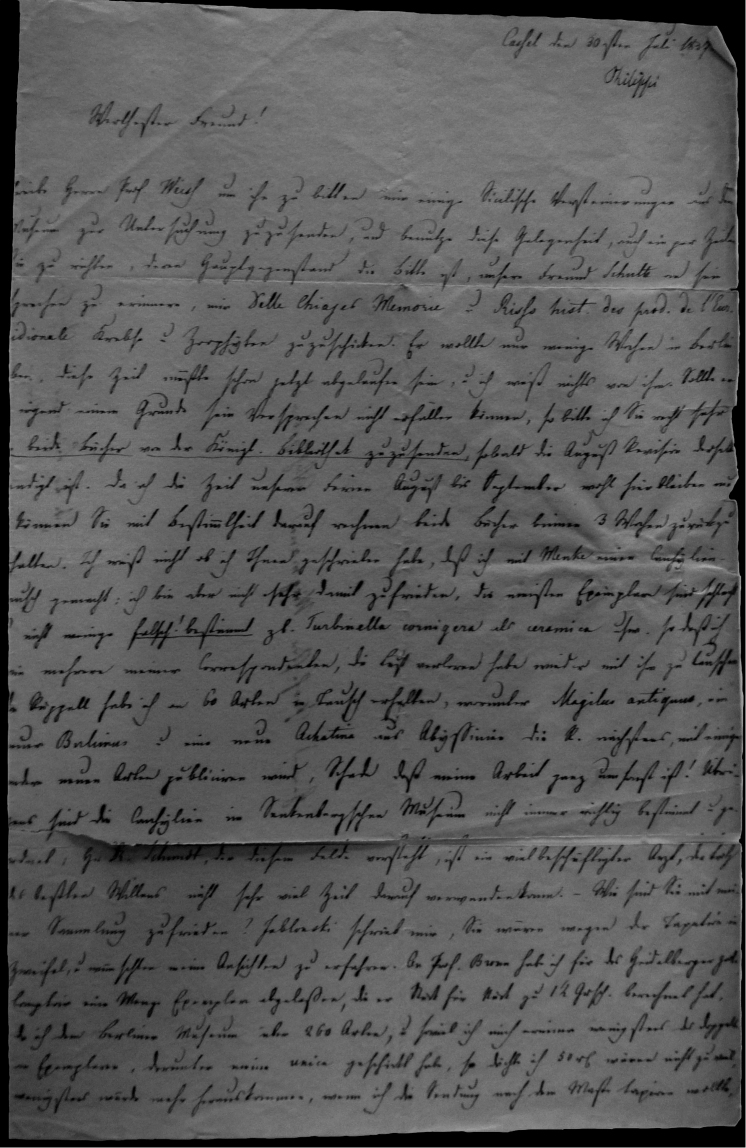
Handwriting of R.A. Philippi. Excerpt from letter d.d. Cassel, 30.vii.1837 (17.5 × 27.5 cm).

**Figure 6.**
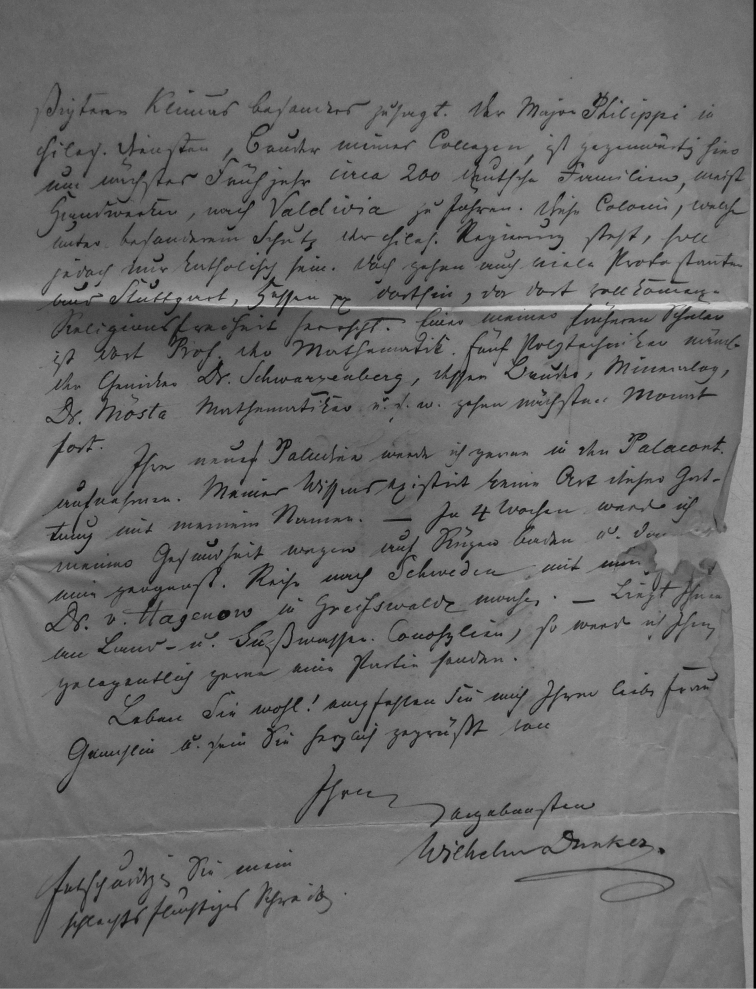
Handwriting of W. Dunker. Excerpt from letter d.d. Cassel, 26.vi.1849 (22 × 27.5 cm).

The importance of handwriting recognition for the deciphering and authorship of labels in collections has recently been illustrated by Breure and Ablett (2011: 5–12). In the Berlin collection I found that often information from the original label has (partly) been copied on a new label, but sometimes the first label has not been kept. E.g., shells originating from the Albers collection are labelled ‘Albers’ on the modern label; on Albers’ labels his source was always noted (e.g. Cuming), but in most cases he has not kept the original label (Fig. 1). Labels were found in the collection that can be atrtibuted to the following authors (with references to biographical data, see also above): J.C. Albers (Fig. 1A–B), W.F. Clapp (Clench 1953; Fig. 1F), A. Doering (Fig. 1E), W.B.R.H. Dunker (Tëmkin et al. 2009; Fig. 1C), H.C. Fulton (Fig. 1D), H. von Heimburg (Fig. 1G), S.H.F. Jaeckel (Fig. 2D), E. von Martens (Glaubrecht and Zorn 2012; Fig. 2B), L. Pfeiffer (Fig. 1B), B. Rensch (Fig. 2C), S.A.A. Petit de la Saussaye (Crosse and Fischer 1871; Fig. 1B), H. Strebel (Fig. 3), J. Thiele (Fig. 2A). Positive identification of authorship was facilitated by the large collection of correspondence in ZMB archives. However, some could only be solved with the help of other sources (e.g. Zilch 1967: 35–37). It may be noted that (slight) differences in handwriting may occur with age, e.g. letters of H. Strebel from the 1870s and his labels from the first decade of the 20th century (Fig. 3). See also the Appendix for a selection of persons, relevant to Neotropical malacology, for which handwritten correspondence is present in the archives. Examples are presented for K.Th. Menke (Fig. 4), R.A. Philippi (Fig. 5), and W. Dunker (Fig. 6).

**Figure 7.**
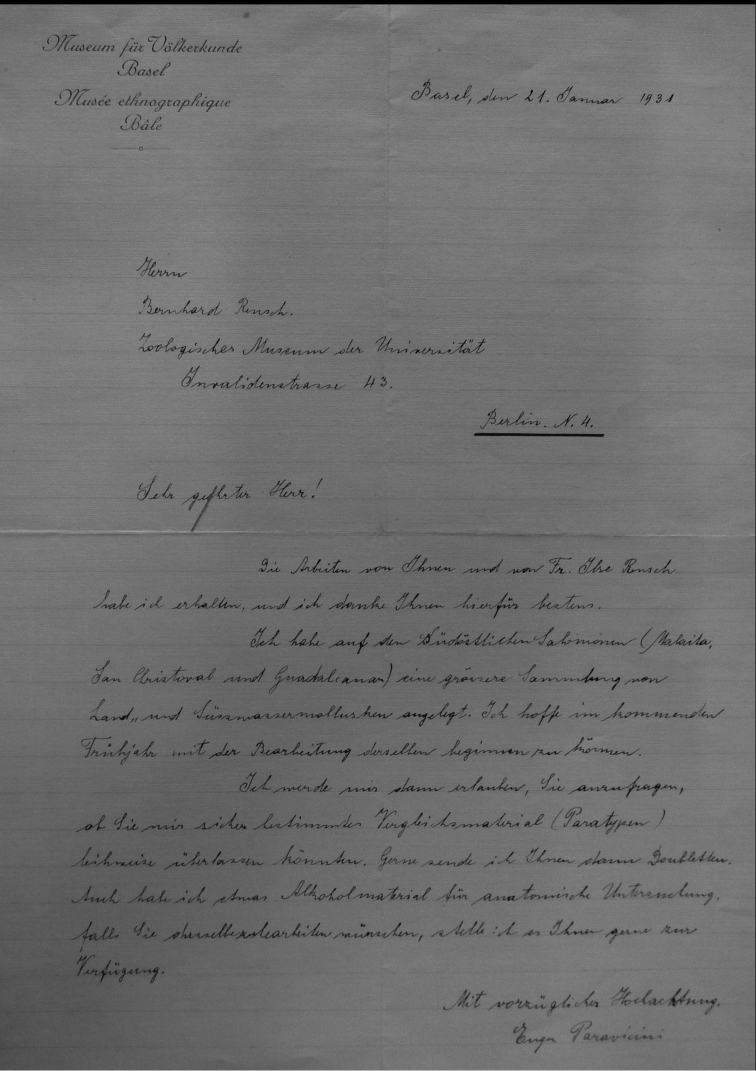
Letter of E. Paravicini to B. Rensch, Basel, 21.i.1931 (21 × 27.5 cm).

Finally, a special note may be made about the B. and I. Rensch material. They studied material obtained from E.M.M. Paravicini. He wrote to B. Rensch (letter d.d. 21.i.1931; Fig. 7) “Ich habe auf den südostlichen Salomonen (Malaita, San Christoval und Guadalcanar) eine grössere Sammlung von Land- und Süsswassermollusken angelegt. Ich hoffe in kommende Frühjahr mit der Bearbeitung derselben beginnen zu können. Ich werde mir dann erlauben, Sie anzufragen, ob Siue mir sicher bestimmter Vergleichsmaterial (Paratypen) leihweise überlassen könnten. Gerne sende ich Ihnen dann Doubletten. Auch habe ich etwas Alkoholmaterial für anatomische Untersuchung, falls Sie derselbe zubearbeiten wünschen, stelle ich es Ihnen gerne zur Verfügung”. Paravicini probably never worked on his material, but trusted this to B. and I. Rensch. It led to their publication (Rensch and Rensch 1934), in which they provided brief diagnoses of new species (two named after Paravicini); a more detailed account based on all material collected by Paravicini was published the next year (Rensch and Rensch 1935). In their first paper they indicated a locality and “Typus”, giving a range of dimensions, except when they had only one shell (“1 Schale = Typus”). In the latter case this is interpreted as a holotype designation, in all other cases their material is considered as syntypes or as paratypes. According to Delsaerdt (2010), who did not mention the ZMB material, the depository of the holotypes is the NMB collection.

The aim of this paper is to present a survey of the types of Orthalicoidea in the ZMB collection, supplementing the earlier paper of Köhler (2007). Therefore, the emphasis lies on the Bothriembryontidae (especially the Placostylinae), Odontostomidae and Orthalicidae, with additional notes on the type material of other families within the superfamily (sensu Breure and Romero 2012). It must be emphasized that the current systematic position under each taxon does not imply a revision, but generally follows the current understanding of recent authors (e.g. Miquel 1993, 1995; Richardson 1993, 1995; Simone 2006; Neubert et al. 2009; Delsaerdt 2010, Thompson 2011).

## Methods

For each taxon, the original publication—in which the taxon was proposed—is mentioned, as well as papers in which reference is made to the type material. The type locality is quoted from the original publication in the original wording and language, with clarifying notes between square brackets. As far as possible, localities have been traced with the NGA gazetteer (http://earth-info.nga.mil/gns/html/ ) or the Fuzzy gazetteer (http://isodp.hof-university.de/fuzzyg/query/ ). The name of the collector, if given in the original paper, is only mentioned (in italics) if it might give a clue about the type status of material present in the collection. The text of the original, or oldest, label is quoted, together with information from subsequent labels if containing information necessary for a correct interpretation. All labels have been photographed and are figured for future historic reference. The dimensions of the type are quoted, as given in the original paper. Dimensions of the type specimens have been taken with a digital caliper; measurements up to 10 mm have an accuracy of 0.1 mm, those above 10 mm are accurate to 0.5 mm. Due to improvements in accuracy of Vernier calipers, the measurements given herein are in several cases slightly different from those originally reported. In the case of syntypes, only the largest specimen has been measured. Under type material the ZMB-registration numbers are given; if specimens from different localities are present, the order of the lots corresponds with the information of the different labels. The number of specimens originally available, if quoted by the original author, is mentioned under remarks. Further remarks are given to describe any individual characteristics of the type specimens or any other details of the type lot. The current systematic position is given, following the generic scheme of Breure (1979) and the familial arrangement of Breure and Romero (2012).

Publication dates of works which have been disputed in literature generally follow the collations of Coan et al. (2012a, 2012b). A special note may be made about the papers of Doering, who in the 1870s published a series on the land and freshwater shells of Argentina. The years in which his papers have been published are variously cited in literature (e.g., Zilch 1971, Neubert and Janssen 2004, Miquel and Aguirre 2011). Since a large number of taxa have been described in these papers, a separate paper has been prepared on the dates of publication of Doering’s work; see Breure and Miquel (2012).

Abbreviations used for depositories of material are: FML, Fundación Miguel Lillo, Tucumán, Argentina; MCZ, Museum of Comparative Zoology, Cambridge, Mass., U.S.A.; MHNG, Musée d’Histoire Naturelle, Genève, Switzerland; MNHN, Muséum nationale d’Histoire naturelle, Paris, France; NMB, Naturhistorisches Museum, Basel, Switzerland; NHMUK, Natural History Museum, London, U.K.; RBINS, Royal Belgian Institute of Natural Sciences, Brussels, Belgium; SMF, Senckenberg Natur-Museum, Frankfurt am Main, Germany; UF, Florida Museum of Natural History, Gainesville, U.S.A.; ZMB, Museum für Naturkunde der Humboldt-Universität, Berlin, Germany; ZMZ, Zoologisches Museum, Universität Zürich, Switzerland. Other abbreviations used are: **/** end of line in cited text; coll., collection; D, diameter; H, shell height; leg., *legit*, collected; W, number of whorls. For the way measurements on the shell have been taken, see Breure and Ablett (2012: fig. 1).

## Data resources

The data underpinning the analyses reported in this paper are deposited in the Dryad Data Repository at doi: 10.5061/dryad.25g11

## Systematics

### Systematic list of taxa arranged in generic order

This systematic list follows Breure (1979); the family classification amended as proposed by Breure and Romero (2012). The generic classification has been adapted from Breure (1979) and unpublished data from the author. It may be noted that ongoing phylogenetic research may alter the classification. Within the family, genus and species level taxa are presented in alphabetical order.

**Family Amphibulimidae P. Fischer, 1873**

***Plekocheilus (Eurytus)*** Albers, 1850

*aulacostylus* Pfeiffer, 1853; *lugubris* Dunker, 1882; *stuebeli* Martens, 1885.

**Family Bothriembryontidae Iredale, 1937**

***Aspastus***Albers, 1850

*manugiensis* B. Rensch, 1934; *paravicinii* B. Rensch, 1934.

***Callistocharis*** Pilsbry, 1900

*paeteli* Kobelt, 1891

***Eumecostylus*** Martens, 1860

*aukiensis* W.F. Clapp, 1923; *phenax* W.F. Clapp, 1923; *uliginosus* Kobelt, 1890.

***Placocharis*** Pilsbry, 1900

*macgillivrayi* Pfeiffer, 1855; *manni* W.F. Clapp, 1923; *strangei* Pfeiffer, 1855; *stutchburyi* Pfeiffer, 1860.

***Placostylus*** Beck, 1837

*eximius* Albers, 1857; *insignis* Petit, 1850; *scarabus* Albers, 1854.

**Family Bulimulidae Tryon, 1867**

***Bostryx*** Troschel, 1847

*conospirus* Doering, 1879; *laurentii* Sowerby I, 1833; *monticola* Doering, 1879; *peristomatus* Doering, 1879; *rhodacme* Pfeiffer, 1842; *rhodolarynx* Reeve, 1849; *stelzneri* Dohrn, 1875; *terebralis* Pfeiffer, 1842; *tortoranus* Doering, 1879.

***Bulimulus*** Leach, 1814

*coriaceus* Pfeiffer, 1857; *monachus* Pfeiffer, 1857.

***Cochlorina*** Jan, 1830

*involutus* Martens, 1867.

***Drymaeus (Mesembrinus)*** Albers, 1850

*bahamensis* Pfeiffer, 1862; *flavidus* Menke, 1829; *livescens* Pfeiffer, 1842; *loxensis* Pfeiffer, 1846; *moussoni* Pfeiffer, 1853; *translucens* Broderip in Broderip & Sowerby I, 1832.

***Lopesianus*** Weyrauch, 1958

*crenulatus* Weyrauch, 1958.

**Remarks.** This taxon is not represented by type material but by topotypes; the holotype and one paratype are present in the SMF collection (Neubert and Janssen 2004: 207). However, as the taxon could not be re-found at the type locality (Rezende & Araujo, pers. commun. 1976; see Breure 1979: 46), the availability of topotypic material may be important to document for future research.

**Family Orthalicidae Martens, 1860**

Corona Albers, 1850

*cincta* Strebel, 1909; *reginaeformis* Strebel, 1909.

Hemibulimus Martens, 1885

*excisus* Martens, 1885; *pallida* Strebel, 1909.

Liguus Montfort, 1810

*archeri* Clench, 1934; *barbouri* Clench, 1929; *blainianus* Poey, 1853.

Orthalicus Beck, 1837

*crosseifischeri* Strebel, 1909; *elegans* Rolle, 1895; *euchrous* Strebel, 1909; *ferussaci* Martens, 1864; *fischeri* Martens, 1893; *gruneri* Strebel, 1909; *isabellinus* Martens, 1873; *jamaicensis* Strebel, 1909; *lividus* Martens, 1864; *maclurae* Martens, 1893; *maculiferus* Strebel, 1909; *miles* Strebel, 1909; *naesiotes* Strebel, 1909; *nobilis* Rolle, 1895; *richardsoni* Strebel, 1909; *selectus* Strebel, 1909; *sphinx* Strebel, 1909; *tricinctus* Martens, 1893; (*maclurae*) *turrita* Strebel, 1909; (*sphinx*) *turrita* Strebel, 1909; *uhdeanus* Martens, 1893; *varius* Martens, 1873; *xanthus* Strebel, 1909.

Sultana (Metorthalicus) Pilsbry, 1899

*carnea* Strebel, 1909; *maranhonensis* Albers, 1854; *shuttleworthi* Albers, 1854.

**Family Odontostomidae Pilsbry and Vanatta, 1894**

Bahiensis Jousseaume, 1877

*albofilosus* Dohrn, 1883; *ringens* Dunker, 1847.

Burringtonia Parodiz, 1944

*pantagruelina* Moricand, 1833.

Cyclodontina Beck, 1837

*trahyrae* Jaeckel, 1950; *tudiculatus* Martens, 1868.

Moricandia Pilsbry and Vanatta, 1898

*willi* Dohrn, 1883.

Odontostomus Beck, 1837

*koenigswaldi* Thiele, 1906; *simplex* Thiele, 1906.

Plagiodontes Doering, 1868

*brackebuschii* Doering, 1877; *rocae* Doering, 1881; *weyenberghii* Doering, 1877.

Spixia Pilsbry and Vanatta, 1898

*achalana* Doering, 1877; *aconjigastana* Doering, 1877; *bergii* Doering, 1877; *bohlsi* Martens, 1894; *champaquiana* Doering, 1877; *leptodon* Martens, 1875; *martensii* Doering, 1875; *multispirata* Doering, 1877; *philippii* Doering, 1875; *reticulata* Doering, 1877; *salinicola* Doering, 1877; *tumulorum* Doering, 1875.

**Remarks.** Breure and Ablett (2012: 4) cited Pilsbry and Vanatta, 1894 in error. E. Salas Oroño (pers. comm.) confirmed that all taxa of Doering listed are considered valid species in her on-going revision of this genus.

**Family Simpulopsidae Schileyko, 1999**

**Remarks.** Schileyko (1999: 324) named the tribe Simpulopsini for the genera *Rhinus*, *Simpulopsis* and *Leiostracus*. This tribe has been raised to family status by Breure and Romero (2012) based on their phylogenetic results.

The taxon *Simpulopsis (Eudioptus) psidii* (Martens, 1877), considered to belong to this group by Köhler (2007), is now placed within the Sagdidae (see below).

### Alphabetic list of taxa by species name

#### Bulimus (Odontostomus) achalanus

Doering, 1877

http://species-id.net/wiki/Bulimus_achalanus

Figs 32D, 32iv

Bulimus (Odontostomus) achalanus Doering 1877: 324; Doering 1878: 243; Neubert and Janssen 2004: 197, pl. 18 fig. 219.
Scalarinella (Spixia) achalana ; Zilch 1971: 199, pl. 12 fig. 16 (lectotype designation).

##### Type locality.

[Argentina, Prov. Córdoba] “Sierra de Achala (Quebrada de Musi)”.

##### Label.

“Sierra de Achala”, taxon label in Martens’ handwriting.

##### Dimensions.

“Long. 21–27mm, lat. 6–7mm”; figured specimen herein H 21.7, D 6.9, W 9.4.

##### Type material.

ZMB 28505, two paralectotypes; ex Doering.

##### Remarks.

The locality matches the type locality. The specimens were directly received from Doering and are herein considered type material. Doering did not mention on how many specimens his description was based upon, but the fact that he gave a range indicates that he had several specimens at hand. The lectotype and four other paralectotypes are in SMF (Neubert and Janssen 2004). The current systematic position is after Richardson (1993).

##### Current systematic position.

Odontostomidae, *Spixia achalana* (Doering, 1877).

#### Bulimus (Odontostomus) aconjigastanus

Doering, 1877

http://species-id.net/wiki/Bulimus_aconjigastanus

Figs 32E, 32v

Bulimus (Odontostomus) aconjigastanus Doering 1877: 326; Doering 1878: 245; Neubert and Janssen 2004: 197, pl. 18 fig. 225.
Scalarinella (Spixia) aconjigastana ; Zilch 1971: 200, pl. 12 fig. 15 (lectotype designation).

##### Type locality.

Not given [Argentina].

##### Label.

“Sierra de Aconjigasta”, taxon label in Martens’ handwriting.

##### Dimensions.

“Long. 18–21mm, lat. 5mm”; figured specimen herein H 20.0, D 5.27, W 10.1.

##### Type material.

ZMB 28503, two paralectotypes; ex Doering.

##### Remarks.

The locality given on the label could not be found in modern gazetteers, but is likely in Prov. Córdoba. The specimens were directly received from Doering and are herein considered type material. Doering did not mention on how many specimens his description was based upon, but the fact that he gave a range indicates that he had several specimens at hand. The lectotype and 24 other paralectotypes are in SMF (Neubert and Janssen 2004). The current systematic position is after Richardson (1993).

##### Current systematic position.

Odontostomidae, *Spixia aconjigastana* (Doering, 1877).

#### Bulimus albofilosus

Dohrn, 1883

http://species-id.net/wiki/Bulimus_albofilosus

Figs 30A–B, 30i

Bulimus albofilosus Dohrn 1883: 351, pl. 11 fig. 7.
Odontostomus albofilosus ; Pilsbry 1901 [1901–1902]: 50, pl. 8 figs 90–91.

##### Type locality.

Not specifically given. From the title and introduction it may be concluded “östlichen Brasilien (...) Quellgebiet des Mucury”.

##### Label.

“Minas geraes, Oberes Mucury”, in handwriting, probably not Dohrn’s.

##### Dimensions.

“Long. 22-24 , diam 6 1/2-7 (...) mm”; figured specimen herein H 22.5, D 7.12, W 7.7.

##### Type material.

ZMB 36424, one syntype; ex Dohrn.

##### Remarks.

As Dohrn mentioned a range in his measurements, he must have based his description on several specimens. Only one shell is present in the ZMB collection. The current systematic position is after Simone (2006).

##### Current systematic position.

Odontostomidae, *Bahiensis albofilosus* (Dohrn, 1883).

#### Liguus fasciatus archeri

Clench, 1934

http://species-id.net/wiki/Liguus_fasciatus_archeri

Figs 19B, 19i

Liguus fasciatus archeri Clench 1934: 106, pl. 7 fig. 5.

##### Type locality.

“Mogote de Ramon Milo, Viñales, Pinar del Rio, Cuba”.

##### Label.

“Mogote de Ramon Milo, Viñales, Cuba”, typewritten, with the name *archeri*—after correction—written in pencil in an unknown handwriting.

##### Dimensions.

“Length 55.5 Width 25.0”; figured specimen herein H 51.9, D 26.1, W 5+.

##### Type material.

ZMB 78796, four paratypes; ex Clench, A.F. Archer leg., vii.1930.

##### Remarks.

The holotype is MCZ 80901. The data of the specimens correspond to the original publication. The current systematic position is after Richardson (1993).

##### Current systematic position.

Orthalicidae, *Liguus fasciatus* (Müller, 1774).

#### Placostylus (Placocharis) hargravesi aukiensis

W.F. Clapp, 1923

http://species-id.net/wiki/Placostylus_hargravesi_aukiensis

Figs 9C, 9i

Placostylus (Placocharis) hargravesi aukiensis W.F. Clapp 1923: 409, fig. 49.
Eumecostylus hargravesi ; Delsaerdt 2010: 47, pl. 8 fig. 3.

##### Type locality.

[Solomon Islands] “Auki, Malaita Id.”.

##### Label.

“Auki, Malaita / Solomon Ids.”, in Clapp’s handwriting.

##### Dimensions.

“G.d. 22 mm. alt. 54.5 mm”; figured specimen herein H 51.1, D 22.0, W 5.1.

##### Type material.

ZMB 74853, two paratypes; W.M. Mann leg..

##### Remarks.

Delsaerdt (2010) recently discussed the systematic position of this taxon and concurred with the opinion of Clench (1941), who synonymized it with *Bulimus hargravesi* Cox, 1871. The holotype is MZC 32442, first figured by Delsaerdt (2010).

##### Current systematic position.

Bothriembryontidae, *Eumecostylus hargravesi* (Cox, 1871).

#### Bulimus aulacostylus

Pfeiffer, 1853

http://species-id.net/wiki/Bulimus_aulacostylus

Figs 8D–E, 8i

Bulimus aulacostylus Pfeiffer 1853: 316.

##### Type locality.

[West Indies] “St. Lucia”.

##### Label.

“India occident. / Inz. St. Luzia”, label in Albers’ handwriting.

##### Dimensions.

“Long. 37; lat. 18 mill.”; figured specimen herein H 37.6, D 21.2, W 4.8.

##### Type material.

ZMB 112723, one probable syntype; ex Albers coll., ex Cuming coll.

##### Remarks.

Pfeiffer described this species from “Mus. Cuming”, without mentioning on how many specimens he based his description on. Type material of this taxon has not been found in the NHMUK collection (Breure and Ablett 2011). It is known that Albers received many shells from Cuming, either directly or indirectly; although a taxon label in Pfeiffer’s handwriting is not present, there is hardly any doubt that this material is a syntype.

##### Current systematic position.

Amphibulimidae, *Plekocheilus (Eurytus) aulacostylus* (Pfeiffer, 1853).

#### Bulimus bahamensis

Pfeiffer, 1862

http://species-id.net/wiki/Bulimus_bahamensis

Figs 17A–B, 17i

Bulimus bahamensis Pfeiffer 1862: 204; Pfeiffer 1868 [1866–1869]: 415, pl. 94 figs 21–24.
Drymaeus bahamensis ; Pilsbry 1899: 8, pl. 13 figs 81–84.

##### Type locality.

[West Indies] “New Providence insulam Bahamensium”.

##### Label.

“Bahama”, label in Dohrn’s handwriting.

##### Dimensions.

“Long. 31; lat. 10 1/2 mill.”; figured specimen herein H 29.4, D 11.8, W 6.7.

##### Type material.

ZMB 25727, two possible syntypes; ex Dohrn.

##### Remarks.

Pfeiffer described this species from material received from Th. Bland. It is known that Pfeiffer exchanged material with Dohrn (Richling and Glaubrecht 2008). Although a taxon label in Pfeiffer’s handwriting is not present, this material is considered a possible syntype.

##### Current systematic position.

Bulimulidae, *Drymaeus (Mesembrinus) bahamensis* (Pfeiffer, 1862).

#### Liguus crenatus barbouri

Clench, 1929

http://species-id.net/wiki/Liguus_crenatus_barbouri

Figs 19C, 19ii

Liguus crenatus barbouri Clench 1929: 18.

##### Type locality.

“Pinecrest region, central Everglades, Fla. Hammock no. 21 (Farnum number). J.N. Farnum, collector.”.

##### Label.

“Hammock #17 / The Everglades / South Florida / U.S.A.” [ZMB 74876], “Hammock #10, Everglades, / Pine Crest Region, Florida” [ZMB 78792].

##### Dimensions.

“Length 51.5 Width 27 (...) mm.”; figured specimen herein H 46.3, D 23.4, W 7.4.

##### Type material.

ZMB 74876, two paratypes; ex Clench. ZMB 78792, two paratypes; ex Clench, J.N. Farnum leg.

##### Remarks.

Clench mentioned in his remarks “Found in nearly all the hammocks of the Pinecrest region”. Although Clench did not mention on how many specimens his description was based, the type status of this material is not disputed. Holotype MCZ 84527.

##### Current systematic position.

Orthalicidae, *Liguus fasciatus* (Müller, 1774).

#### Bulimus (Odontostomus) bergii

Doering, 1877

http://species-id.net/wiki/Bulimus_bergii

Figs 32F, 32vi

Bulimus (Odontostomus) bergii Doering 1877: 327; Doering 1878: 246; Neubert and Janssen 2004: 201, pl. 18 fig. 221.
Scalarinella (Spixia) aconjigastana ; Zilch 1971: 200, pl. 12 fig. 23 (lectotype designation).

##### Type locality.

[Argentina, Prov. Córdoba] “*a. var*. de Alta Gracia [D.C. Berg leg] (...) *b*. Cuesta de S. Antonio (Sierra Chica), region de Coco y Moya (...) *c*. Cerro Salado (S. de Aconjigastana, pendiente Oeste) (...) *d*. Pozo de Piedra (S. de Aconjigasta, pendiente Oeste)”.

##### Label.

“Sierra de Aconjigasta”, taxon label in Martens’ handwriting.

##### Dimensions.

“Long. 16–22mm, lat. 5–6mm”; figured specimen herein H 19.2, D 5.28, W 10.2.

##### Type material.

ZMB 28508, two paralectotypes; ex Doering.

##### Remarks.

The locality given on the label could not be found in modern gazetteers, but is likely in Prov. Córdoba. Doering did not mention on how many specimens his description was based upon, but the fact that he gave a range indicates that he had several specimens at hand. The lectotype and five other paralectotypes are in SMF (Neubert and Janssen 2004). The current systematic position is after Richardson (1993).

##### Current systematic position.

Odontostomidae, *Spixia bergii* (Doering, 1877).

#### Achatina blainiana

Poey, 1853

http://species-id.net/wiki/Achatina_blainiana

Figs 19A, 19v

Achatina blainiana Poey 1853 [1851–1861]: 206, pl. 12 figs 4–6; Pfeiffer 1865 in Küster and Pfeiffer 1840–1865: 364, pl. 24 figs 4–5.

##### Type locality.

[Cuba] “la loma de Rangel, á unas 30 leguas S–S.O. de la Habana”.

##### Label.

“294. Rangel / Gundl.”, “Cuba. Poey / Felipe Poey”.

##### Dimensions.

“Long. 43 millimetros; diametro 18”; figured specimen herein H 39.0, D 18.2, W 7.3.

##### Type material.

ZMB 117781, four probable syntypes; ex Dunker, ex Poey, J. Gundlach leg.

##### Remarks.

The locality on the label correspond with the type locality given by Poey (1853 [1851–1861]), who did not state on how many specimens his description was based. Moreover, the specimens were collected by Gundlach, who is known to have collected many shells for Poey; see also Richling and Glaubrecht (2008: 271). Analogous to their reasoning on the type status of taxa described by Pfeiffer (Richling and Glaubrecht 2008: 268, sub (i)), we here consider it plausible that ZMB 117781 is to be considered as probable syntypes since these are labelled as received from Poey. For details on the connection between Gundlach and Dunker (and Pfeiffer) see Glaubrecht in Tëmkin et al. (2009).

Two other lots are present: ZMB 9044, three specimens; labelled “Rangel, Cuba”, J. Gundlach leg., and ZMB 294, three specimens; labelled “Rangel”, ex Dunker. Although this material is from the same source, the specimens are not considered syntypes in the sense of the ICZN.

##### Current systematic position.

Orthalicidae, *Liguus fasciatus* (Müller, 1774).

#### Odontostomus striatus bohlsi

Martens, 1894

http://species-id.net/wiki/Odontostomus_striatus_bohlsi

Figs 32H, 32viii

Odontostomus striatus bohlsi Martens 1894: 166.

##### Type locality.

[Paraguay] “Barranca de la Novia”.

##### Label.

Paraguay / Barranca / de la Novia”, with a second, taxon label in Martens’ handwriting.

##### Dimensions.

“48–50 mm lang und nur 12–13 dick im Querdurchmesser mit Einrechnung der Mündung”; figured specimen herein H 50.2, D 12.6, W 13.8.

##### Type material.

ZMB 47507, eight syntypes; Bohls leg.

##### Remarks.

Parodiz (1942: 201) synonymized this as variety described taxon by Martens with “*Odontostomus spixi* var. *major* (d’Orb.)”, stating that d’Orbigny’s taxon has 12.5 whorls; the lectotype of *major* d’Orbigny, 1837 designated by Breure and Ablett (2011: 25) has 10.6 whorls and a shell height of 34.8 mm. Subsequently, this and other varieties were synonymized with *Spixia striata* (Spix, 1827) by Richardson (1993). The fact that Martens (1894) recognized four varieties—ranging from 26–50 mm shell height and 10 1/2–14 whorls—from the same locality, calls for an in-depth study of the variability of *Spixia striata*. Provisonally, the systematic position by Richardson (1993) is here retained.

##### Current systematic position.

Odontostomidae, *Spixia striata* (Spix, 1827).

#### Bulimulus bonneti

Ancey, 1902

http://species-id.net/wiki/Bulimulus_bonneti\according_to_Breure_2013

Bulimulus bonneti Ancey 1902: 40, fig. 1; Köhler 2007: 131, fig. 22; Wood and Gallichan 2008: 29; Breure 2011: 17, figs 11A, 11i.

##### Remarks.

Köhler (2007) reported one possible syntype (ZMB 62578) with label “Bolivia”, received from Bonnet. It is, however, not accompanied by a label in Ancey’s handwriting and therefore not considered type material. Type material is present in the MNHN (lectotype) and RBINS (one paralectotype) collections (Wood and Gallichan 2008, Breure 2011).

#### Bulimus (Plagiodontes) brackebushii

Doering, 1877

http://species-id.net/wiki/Bulimus_brackebushii

Figs 32A, 32i

Bulimus (Plagiodontes) brackebushii Doering 1877: 321; Doering 1878: 240; Neubert and Janssen 2004: 202, pl. 20 fig. 252.
Scalarinella (Plagiodontes) brackebushii Doering; Zilch 1971: 198, pl. 11 figs 6–7 (lectotype designation).

##### Type locality.

[Argentina, Prov. San Luis] “en la Sierra de S. Luis, cerca de S. Francisco”.

##### Label.

“Sierra de S. Luis”, with a second, taxon label in Martens’ handwriting.

##### Dimensions.

“Long. 26mm; lat. 11mm”; figured specimen herein H 25.1, D 11.32, W 7.4.

##### Type material.

ZMB 28511, one paralectotype; ex Doering, D.L. Brackebush leg.

##### Remarks.

Doering did not mention on how many specimens his description was based upon. Zilch (1971) mentioned two specimens in SMF, of which he has chosen one as lectotype. The specimen in ZMB was received directly from Doering and is herein considered a paralectotype.

**Current systematic position.** Odontostomidae, *Plagiodontes brackebushii* (Doering, 1877).

#### Orthalicus meobambensis carnea

Strebel, 1909

http://species-id.net/wiki/Orthalicus_meobambensis_carnea

Figs 28A–B, 28i

Orthalicus meobambensis carnea Strebel 1909: 149, pl. 19 fig. 428.

##### Type locality.

[Peru] “Meobamba”.

##### Label.

“Meobamba”, in Albers’ handwriting.

##### Dimensions.

Not given; figured specimen herein H 68.7, D 42.8, W 6.3.

##### Type material.

ZMB 101823, holotype; ex Albers coll. No. 558, ex Cuming coll.

##### Remarks.

Besides the Albers’ label and the second label, previously mentioned by Strebel (“This shell cost me a Guinea”), there is a third label “zwischen Dennisoni / cf. Rv 166 / u. gall.sultana / aber nicht zu trullisatus / Shuttleworth”. As Strebel based himself explicitly on one specimen and mentioned “Berl. Museum”, the specimen found is the holotype.

##### Current systematic position.

Orthalicidae, *Sultana (Metorthalicus) meobambensis* (Pfeiffer, 1855).

#### Bulimus (Odontostomus) champaquianus

Doering, 1877

http://species-id.net/wiki/Bulimus_champaquianus

Figs 32G, 32vii

Bulimus (Odontostomus) champaquianus Doering 1877: 330; Doering 1878: 249; Neubert and Janssen 2004: 203, pl. 18 fig. 231.
Scalarinella (Spixia) champaquiana ; Zilch 1971: 200, pl. 12 fig. 25 (lectotype designation).

##### Type locality.

[Argentina, Provs. Córdoba and San Luis] “en la pendiente Sudoeste de la Sierra de Achala. (...) la pendiente Este de la Sierra de Aconjigasta, cerca de Nono (...) la Quebrada del Rio de Mina Clavero (...) la Quebrada de Oyada, en la Provincia de S. Luis”.

##### Label.

“Sierra de Aconjigasta”, taxon label in Martens’ handwriting.

##### Dimensions.

“Long. 15–16mm, lat. 4mm”; figured specimen herein H 14.1, D 4.48, W 8.5.

##### Type material.

ZMB 28512, four paralectotypes; ex Doering.

##### Remarks.

The locality given on the label could not be found in modern gazetteers, but is likely in Prov. Córdoba. The specimens were directly received from Doering and are herein considered as type material. Doering did not mention on how many specimens his description was based upon, but the fact that he gave a range indicates that he had several specimens at hand. The lectotype and ten other paralectotypes are in SMF (Neubert and Janssen 2004). The current systematic position is after Richardson (1993).

**Current systematic position.** Odontostomidae, *Spixia champaquiana* (Doering, 1877).

#### Corona pfeifferi cincta

Strebel, 1909

http://species-id.net/wiki/Corona_pfeifferi_cincta

Figs 18A–B, 18i

Corona pfeifferi cincta Strebel 1909: 135, pl. 21 fig. 337, pl. 22 figs 356–357.

##### Type locality.

“Ecuador”.

##### Label.

“Ecuador”, with a second, taxon label in Strebel’s handwriting.

##### Dimensions.

“55,2 × (23,4)25,0 [H × (Dmin)Dmax in mm]”; figured specimen herein H 55.0, D 25.3, W 7.2.

##### Type material.

ZMB 101836, one syntype; ex Paetel coll.

##### Remarks.

Strebel described this taxon on the basis of two specimens available to him, one from “des Berliner Museums aus der Paetelschen Sammlung”. The type status of this specimen is thus not disputed.

##### Current systematic position.

*Corona pfeifferi* (Hidalgo, 1869).

#### Bulimulus (Scutalus) conispirus

Doering, 1879

http://species-id.net/wiki/Bulimulus_conispirus

Figs 15B, 15ii

Bulimulus (Scutalus) conispirus Doering 1879: 67.

##### Type locality.

[Argentina] “la sierra de Tucuman”.

##### Label.

“Oran (Rep. Arg.)”, taxon label in Doering’s handwriting.

##### Dimensions.

“Long. 21–24mm, lat. 13–16 1/2mm”; figured specimen herein H 21.9, D 14.4, W 4.7.

##### Type material.

ZMB 34721, one syntype; ex Doering.

##### Remarks.

Doering did not mention on how many specimens his description was based; however, he gave a range in measurements indicating more than one specimen. The material was received directly from Doering and there is but little doubt about its type status. There are three localities with the name Orán in Argentina: two in Prov. Tucúman, both south of San Miguel de Tucumán, and one in Prov. Salta. The data on the label are thus more specific than the published locality in Doering’s paper. The current systematic position is following Miquel (1993).

##### Current systematic position.

Bulimulidae, *Bostryx stelzneri* (Dohrn, 1875).

#### Bulimus coriaceus

Pfeiffer, 1857

http://species-id.net/wiki/Bulimus_coriaceus

Figs 16A, 16i

Bulimus coriaceus Pfeiffer 1857a: 318.

##### Type locality.

[Mexico, Edo. Veracruz] “Cordova”.

##### Label.

“Mexico”, in Albers’ handwriting.

##### Dimensions.

“Long. 18, diam. 9 mill.”; figured specimen herein H 12.92, D 8.42, W 5.1.

##### Type material.

ZMB 117767, one syntype; ex Albers coll. No. 586, ex Cuming coll.

##### Remarks.

This species was described from material collected by Sallé in the Proceedings of the Zoological Society of London; type specimens of other species described in the same paper have been found in the NHMUK collection, but not of this taxon (Breure and Ablett, in preparation). Although the shell from the Albers collection is not accompanied by a label in Pfeiffer’s handwriting, its type status is not disputed herein. It is likely a subadult shell. The current systematic position is after Thompson (2011).

##### Current systematic position.

Bulimulidae, *Bulimulus coriaceus* (Pfeiffer, 1857).

#### Lopesianus crenulatus

Weyrauch, 1958

http://species-id.net/wiki/Lopesianus_crenulatus

Lopesianus crenulatus Weyrauch 1958: 121, pl. 6 figs 7–8; Neubert and Janssen 2004: 207, pl. 17 fig. 218.

##### Remarks.

ZMB 101037 is a lot with five specimens, of which three subadult and one juvenile. It was identified as *Bulimulus gorrietiensis* Pilsbry, 1896 by the collector, H. [de] S[ouza]. Lopes. The specimens were collected in March 1951. Weyrauch (1958) based himself on material from the same collector and the same locality to describe his *Lopesianus crenulatus*, but did not include the ZMB specimens in his type series; they are considered as topotypes (see also page 6).

#### Zebra crosseifischeri

Strebel, 1909

http://species-id.net/wiki/Zebra_crosseifischeri

Figs 20A–B, 20i

Zebra crosseifischeri Strebel 1909: 27, pl. 1 fig. 4, pl. 2 fig. 17.

##### Type locality.

“San Isidro, Guatemala”.

##### Label.

“S[an] Isidro Guatemala”, with a second, taxon label in Strebel’s handwriting.

##### Dimensions.

“51,5 × (25,9)31,3 [H × (Dmin)Dmax in mm]”; figured specimen herein H 50.2, D 29.3, W 6.0.

##### Type material.

ZMB 109951, holotype; Champion leg.

##### Remarks.

Strebel based himself on “Ein Stück des Berliner Museums”. Both from his text and from his label with the specimen, it is clear that the original description was based on one of the specimens which Martens (1893 [1890–1901]) described as *Orthalichus princeps fischeri* (see also remarks under this taxon, page 20). Strebels taxon is thus a junior objective synonym (**syn. n.**).

##### Current systematic position.

Orthalicidae, *Orthalicus princeps fischeri* Martens, 1893.

#### Orthalicus elegans

Rolle, 1895

http://species-id.net/wiki/Orthalicus_elegans

Figs 19D–E, 19iii

Orthalicus elegans Rolle 1895: 131; Neubert and Janssen 2004: 237, pl. 23 fig. 280.
Orthalichus princeps elegans ; Martens 1901 [1890–1901]: 629, pl. 44 fig. 15.

##### Type locality.

[Mexico] “Colima”.

##### Label.

“Colima / Mex”, in Martens’ handwriting.

##### Dimensions.

“Alt. 62, diam. 28.5 (...) mm.”; figured specimen herein H 61.2, D 28.5, W 5+.

##### Type material.

ZMB 47655, lectotype; ex Rolle.

##### Remarks.

Rolle did not state on how many spcimens his description was based. The top of the specimen is damaged. There is no original label in Rolle’s handwriting, but the measurements agree and Martens has marked the specimen as ‘type’ on the label. Neubert and Janssen (2004) correctly indicated that Rolle distributed more specimens under the same name, without it being clear if they were part of the original series. This being the case, I now designate the ZMB specimen as lectotype (**design. n.**) to define the taxon. The current systematic position is after Thompson (2011).

##### Current systematic position.

Orthalicidae, *Orthalicus elegans* Rolle, 1895.

#### Zebra zoniferus euchrous

Strebel, 1909

http://species-id.net/wiki/Zebra_zoniferus_euchrous

Figs 21E–F, 21iii

Orthalichus [sic] *zoniferus*; Martens 1893 [1890–1901]: 186, pl. 10 fig. 12.
Zebra zoniferus euchrous Strebel 1909: 52, pl. 8 figs 120–123, 127, pl. 9 figs 128–129.

##### Type locality.

[Mexico] “dem Staate Oaxaca (...) Venta de Zopilote, Staat Guerrero”.

##### Label.

“Venta de Zopilote / Prov Guerrero / 2800´”.

##### Dimensions.

“42,4 × (22,5)27,3 [H × (Dmin)Dmax in mm]”; figured specimen herein H 41.6, D 25.0, W 6.1.

##### Type material.

ZMB 28001, two syntypes; H.H. Smith leg.

##### Remarks.

Martens (1893) and Strebel (1909) both based new species descriptions on material in the ZMB; Strebel had access to 14 additional specimens from the Hamburg museum when he described this taxon. The specimen figured corresponds to Strebel’s pl. 9 figs 128–129. The current systematic position is following Richardson (1993).

##### Current systematic position.

Orthalicidae, *Orthalicus zoniferus* Strebel and Pfeffer, 1882.

#### Liguus (Hemibulimus) excisus

Martens, 1885

http://species-id.net/wiki/Liguus_excisus

Figs 18E–F, 18iii

Liguus (Hemibulimus) excisus Martens 1885: 173, pl. 35 figs 1–2, 4–5.
Liguus (Hemibulimus) magnificus ; Pilsbry 1899: 185, pl. 36a figs 31–34.
Porphyrobaphe (Hemibulimus) excisus ; Strebel 1909: 108, pl. 23 fig. 363.

##### Type locality.

“Columbiae (Novae Granadae) prope Popayan, circa 2400 Met., leg. Dr. A. Stübel”.

##### Label.

“Popayan”, in unknown handwriting and the name “(excisus*)” added in a later hand.

##### Dimensions.

“Long. 44, diam. maj. 22 (...) Millim.”; figured specimen herein H 43.1, D 22.0, W 5.9.

##### Type material.

ZMB 101837, holotype; A. Stübel leg.

##### Remarks.

Martens mentioned “nur ein Exemplar von Herrn Stübel gesammelt wurde”; the specimen in the ZMB collection is thus the holotype. The current systematic postion is after Richardson (1993).

##### Current systematic position.

Orthalicidae, *Hemibulimus excisus* (Martens, 1885).

#### Bulimus eximius

Albers, 1857

http://species-id.net/wiki/Bulimus_eximius

Figs 11A, 11i

Bulimus eximius Albers, 1857: 96; Neubert et al. 2009: 63, 65.

##### Type locality.

“Nova Caledonia”.

##### Label.

“Nova Caledonia”, in Albers’ handwriting.

##### Dimensions.

“Longit. 119, diam. 60 millim. (...) Specim. maxim. / Longit. 106, diam. 55 millim. (...) Specim. alterum.”; specimen figured herein H 119.1, D 61.0, W 7.0.

##### Type material.

ZMB 117761, two syntypes; ex Albers coll. No. 550 and 552 respectively, Marguier leg.

##### Remarks.

Albers based his description on three specimens, of which one belonged to Mousson’s collection. According to Neubert et al. (2009: 65) this specimen could not be found in the ZMZ collection. The dimensions of the smallest specimen correspond to those given by Albers (1857: 97) in his remarks. The current systematic position is after Neubert et al. (2009).

##### Current systematic position.

Bothriembryontidae, *Placostylus fibratus souvillei* (Morelet, 1857).

#### Orthalicus ferussaci

Martens, 1864

http://species-id.net/wiki/Orthalicus_ferussaci

Figs 22C–D, 22ii

Orthalicus ferussaci Martens 1864: 542; Martens 1873: 188, pl. 1 fig. 6.
Orthalichus ferussaci ; Martens 1893 [1890–1901]: 184, pl. 10 figs 8–10.

##### Type locality.

[Mexico] “Bei Tehuantepec, am südlicheren Theil der Westküste”.

##### Label.

“Mexico”, “Tehuantepec”, taxon label in Martens’ handwriting.

##### Dimensions.

“Long. 65, diam. 33 (...) Millim.”; figured specimen herein H 39.5, D 24.3, W 6.0.

##### Type material.

ZMB 4599, one syntype; Deppe leg.

##### Remarks.

Martens (1864) did not mention the number of specimens his description was based upon. The (subadult) specimen found in the ZMB collection does not confirm to the original measurements; it corresponds to the figure given in Martens (1893 [1890–1901]), but not to the figure given in Martens (1873). The current systematic position follows Thompson (2011).

##### Current systematic position.

Orthalicidae, *Orthalicus ferussaci ferussaci* Martens, 1864.

#### Orthalicus princeps fischeri

Martens, 1893

http://species-id.net/wiki/Orthalicus_princeps_fischeri

Figs 20C–D, 20ii

Orthalicus princeps Broderip; Fischer and Crosse 1874 [1870–1878]: pl. 18 fig. 2b [figure only].
Orthalichus [sic] *princeps fischeri*Martens 1893 [1890–1901]: 183 [not pl. 10 fig. 7].
Zebra crosseifischeri Strebel 1909: 27, pl. 1 fig. 4, pl. 2 fig. 17.

##### Type locality.

“W. Guatemala: El Reposo and San Isidro near Mazatenango (*Champion*)”.

##### Label.

“El Reposo Guatemala” [ZMB 109950], “S[an] Isidro Guatemala” [ZMB 109951], taxon label in Martens’ handwriting.

##### Dimensions.

Not given; figured specimen herein H 60.0, D 35.9, W 6.3.

##### Type material.

ZMB 109950, one syntype. ZMB 109951, one syntype; both Champion leg.

##### Remarks.

Martens did not mention the number of specimens his description was based upon. The specimens found in the ZMB collection do not confirm to the figure given in Martens (1893 [1890–1901]). Both have later been re-identified by Strebel: ZMB 109950 is figured in Strebel 1909: pl. 2 fig. 23 as *Zebra fischeri*; ZMB 109951 on pl. 2 fig. 17 as *Zebra crossei-fischeri*. The latter taxon was introduced by Strebel (1909: 27) as a new species, but from his text it is clear that he based himself on the same specimen which Martens (1893 [1890–1901]) regarded as his *Orthalichus fischeri*. The name *Zebra crosseifischeri* Strebel, 1909 is thus an objective synonym of *Orthalicus fischeri* Martens, 1893 (**syn. n.**). See also under *Zebra crosseifischeri* Strebel, 1909, page 17. The current systematic position follows Thompson (2011).

##### Current systematic position.

Orthalicidae, *Orthalicus princeps fischeri* Martens, 1893.

#### Bulimus flavidus

Menke, 1829

http://species-id.net/wiki/Bulimus_flavidus

Figs 17C–D, 17ii

Bulimus flavidus Menke 1829: 6.

##### Type locality.

Not given.

##### Label.

“v. Malsburg”, in Martens’ handwriting.

##### Dimensions.

Not given; figured specimen herein H 23.4, D 10.7, W 5.7.

##### Type material.

ZMB 10338, three probable syntypes; ex von Malsburg coll.

##### Remarks.

The specimens are only accompanied by a label that states they belonged to the von Malsburg collection, which has been described by Menke (1829). The label gives three names, viz. “*Bulimus liliaceus* Fér.”, “*Bul. flavidus* Mke”, and “*Bulimus roseoflavus* m”; the latter name is an unpublished name, in different ink and probably later added. The “m” may possibly refer to “mihi” and would indicate that Martens added this name. Since the specimens originate from the von Malsburg collection and bear Menke’s name (who is known to have corresponded with the ZMB, see below), they are here regarded as probable syntypes.

##### Current systematic position.

Bulimulidae, *Drymaeus (Mesembrinus) flavidus* (Menke, 1829).

#### Zebra gruneri

Strebel, 1909

http://species-id.net/wiki/Zebra_gruneri

Figs 20E–F, 20iii

Orthalicus maracaibensis ; Martens 1873: 188, pl. 1 fig. 7.
Zebra gruneri Strebel 1909: 63, pl. 16 figs 252–253.

##### Type locality.

[Venezuela] “Maracaibo”.

##### Label.

“Maracaibo”, in Albers’ handwriting.

##### Dimensions.

“57,8 × (28,0)32,6 [H × (Dmin)Dmax in mm]”; figured specimen herein H 57.4, D 31.8, W 6.4.

##### Type material.

ZMB 117783, holotype; ex Albers coll. No. 37, Grüner leg.

##### Remarks.

Martens (1873) and Strebel (1909) both based themselves on material in the ZMB. Strebel mentioned “Das Stück stammt aus den Albersschen Sammlung und ist von Grüner in Maracaibo gefunden”, thus he implicitly described this taxon from one shell. The specimen found corresponds to the figures given by Strebel, and is regarded the holotype. There is also a label indicating that Albers had two specimens (“37–38”), but only one specimen is present and was mentioned by Strebel (1909). The current systematic position is following Richardson (1993).

##### Current systematic position.

Orthalicidae, *Orthalicus gruneri* (Strebel, 1909).

#### Bulimus insignis

Petit de la Saussaye, 1850

http://species-id.net/wiki/Bulimus_insignis

Figs 11B, 11ii

Bulimus insignis Petit de la Saussaye 1850: 57, pl. 3 fig. 1.

##### Type locality.

“nous cryons appartenir à la Nouvelle-Calédonie”; see remarks.

##### Label.

“oc:pacif:”, in Petit’s handwriting.

##### Dimensions.

“Long. 65 mill.”; specimen figured herein H 61.9, D 31.5, W 6.8.

##### Type material.

ZMB 117762, one syntype; ex Albers coll. No. 375, ex Petit.

##### Remarks.

Petit did not mention on how many specimens his description was based upon; however, since he mentions a variety, he must have seen more than one specimen. Although the original label does not mention the published locality, Petit suggested explicitly New Caledonia in his paper. Therefore, the type status is not disputed. The current systematic position is after Neubert et al. (2009: 53), who considered the type material “very probably lost”.

##### Current systematic position.

Bothriembryontidae, *Placostylus fibratus fibratus* (Martyn, 1784).

#### Zebra delphinus intermedius

Strebel, 1909

http://species-id.net/wiki/Zebra_delphinus_intermedius

Figs 21A–B, 21ii

Zebra delphinus intermedius Strebel 1909: 35, pl. 16 figs 254–255.

##### Type locality.

“angeblich Costarica”.

##### Label.

“angeblich Costarica”, with a second taxon label in Strebel’s handwriting.

##### Dimensions.

“59,8 × (27,3)32,7 [H × (Dmin)Dmax in mm]”; figured specimen herein H 57.4, D 31.8, W 6.4.

##### Type material.

ZMB 21848, holotype; Von Patten leg.

##### Remarks.

Strebel (1909: 35–36) mentioned two shells from ZMB with a label “Costa rica vPatten”, one with a Martens’ label “*zoniferus* var. *crossei*” [which Strebel held for “ein typischer *Zebra maracaibensis var. jamaicensis*”]; the other was labelled “*Orthalicus undatus* Brug.” and corresponds to the figured specimen by Strebel. As he implicitly described this taxon from one shell, this is thus the holotype.

##### Current systematic position.

Orthalicidae, *Orthalicus delphinus* (Strebel, 1909).

#### Bulimus involutus

Martens, 1867

http://species-id.net/wiki/Bulimus_involutus

Figs 16D–F

Bulimus involutus Martens 1867: 63.

##### Type locality.

“Brasilien (...) angeblich (...) bei Bahia”; see remarks.

##### Label.

No label.

##### Dimensions.

“Diam. maj. ?, min. 18 1/2, alt. testae=alt. aperturae 15 Mm.”; figured specimen herein H 15.2, D -, W 4.5.

##### Type material.

ZMB 117768, holotype; see remarks.

##### Remarks.

Martens (1867) based his description on one shell with a damaged aperture (“Ein Exemplar mit verletzten Mündung im zoologischen Museum in Berlin”), which was said to have been collected by Kähne near Bahia. The label is lost, but the damaged specimen undoubtedly is the shell described by Martens and is thus the holotype. The current systematic position is after Simone (2006: 134), who has figured a non-type specimen from the ZMB collection.

##### Current systematic position.

Bulimulidae, *Cochlorina involuta* (Martens, 1867).

#### Orthalicus isabellinus

Martens, 1873

http://species-id.net/wiki/Orthalicus_isabellinus

Figs 22G–H, 22iv

Orthalicus isabellinus Martens 1873: 190, pl. 1 fig. 8; Pilsbry 1899: 142, pl. 31 fig. 66.
Zebra fischeri Martens; Strebel 1909: 27, 29, pl. 3 figs 33–34, 37.

##### Type locality.

“Peru”.

##### Label.

“Peru”.

##### Dimensions.

“Long. 49, diam. 23 1/2 (...) Millim.”; figured specimen herein H 37.0, D 23.6, W 5.9.

##### Type material.

ZMB 8876, two syntypes; ex Neuchatel Museum, Tschudi leg.

##### Remarks.

Martens writes “von Tschudi gesammelt, in der Albers’schen Sammlung: mehrere Exemplare vom Neuchateler Museum für das Berliner erhalten”. Two subadult specimens, not corresponding to the original measurements; it may be possible that one or more specimens have been lost. There are no labels in the handwriting of neither Albers nor Martens, but the type status is herein not disputed. One of the shells corresponds to the figure given by Martens (1873: pl. 1 fig. 8), of which Strebel (1909: 29) remarked “nicht gerade sehr characteristisch”; this shell was refigured in his pl. 3 figs 33–34 and attributed to the Guatemalan taxon *Orthalicus princeps fischeri* Martens, 1893. However, assuming that the locality is correct, we cannot agree with Strebel’s re-identification and assign this taxon to *Orthalicus bensoni* (Reeve, 1849) (**syn. n.**).

##### Current systematic position.

Orthalicidae, *Orthalicus bensoni* (Reeve, 1849).

#### Zebra maracaibensis jamaicensis

Strebel, 1909

http://species-id.net/wiki/Zebra_maracaibensis_jamaicensis

Figs 22A–B, 22i

Zebra maracaibensis jamaicensis Strebel 1909: 90, pl. 20 figs 314–315, 319–320, 322–326.

##### Type locality.

“Jamaica”.

##### Label.

“angeblich Costarica” [ZMB 21848a]; “Kingston, Jamaika” [ZMB 50676]; labels referring to Strebel’s figures in his handwriting.

##### Dimensions.

“54,9 × (27,6)32,8 [H × (Dmin)Dmax in mm]”; figured specimen herein H 54.9, D 31.7, W 6.7.

##### Type material.

ZMB 21848a, one syntype; Von Patten leg. ZMB 50676, three syntypes, Hoppe leg.

##### Remarks.

Strebel (1909: 35–36) mentioned two shells from ZMB with a label “Costa rica vPatten”, one with a Martens’ label “*zoniferus* var. *crossei*” [which Strebel held for “ein typischer *Zebra maracaibensis var. jamaicensis*”]; this is one of the shells mentioned by Strebel (1909: 91) under his “Form *jamaicensis*”. Only one specimen was found and this shell corresponds to Strebel’s pl. 20 fig. 324. The locality of this specimen is probably erroneous. Strebel mentioned also several specimens from Jamaica, present in the ZMB collection and collected by Hoppe (ZMB 50676); these are the shells corresponding to Strebel pl. 20 figs 319–320. The current systematic position is after Richardson (1993).

##### Current systematic position.

Orthalicidae, *Orthalicus maracaibensis* (Pfeiffer, 1856).

#### Bulimuss jonasi

Pfeiffer, 1846

http://species-id.net/wiki/Bulimuss_jonasi

Figs 16C, 16iii

Bulimus jonasi Pfeiffer in Philippi 1846 [1845–1847]: 125, pl. 5 fig. 4; Breure 1979: 120.
Drymaeus (Mesembrinus) jonasi ; Thompson 2011: 125.

##### Type locality.

“Vera Cruz Americae centralis (Lattre in coll. Cuming)”.

##### Label.

“Vera Cruz”, in Albers’ handwriting.

##### Dimensions.

“Long 13´´´, Diam. 5´´´ [H 28.3, D 10.9 mm]”; figured specimen herein H 21.5, D 10.3, W 5.0.

##### Type material.

ZMB 117769, one syntype; ex Albers coll. No. 139, ex Cuming coll.

##### Remarks.

The specimen has been received through Albers from the Cuming collection, and there is no doubt about its type status. According to Thompson (2011) three other syntypes are present in NHMUK 1975557; the current systematic position is following this author.

##### Current systematic position.

Bulimulidae, *Drymaeus (Mesembrinus) jonasi* (Pfeiffer in Philippi, 1846)

#### Macrodontes koenigswaldi

Thiele, 1906

http://species-id.net/wiki/Macrodontes_koenigswaldi

Figs 31D–F, 31ii

Macrodontes koenigswaldi Thiele 1906: 69, fig. 2a; Simone 2006: 162, fig. 557.

##### Type locality.

[Brazil] “Serra do Mar (Rio grande do Sul)”.

##### Label.

“Serra do Mar, R. gr do Sul”, in Thiele’s handwriting.

##### Dimensions.

“Höhe etwas über 30 mm, Dicke 11 mm”; figured specimen herein H 30.5, D 10.7, W 6.0.

##### Type material.

ZMB 55780, holotype; von Königswald leg.

##### Remarks.

The current systematic position follows the generic classification of Schieleyko (1999) and the species status of Simone (2006).

##### Current systematic position.

Odontostomidae, *Odontostomus koenigswaldi* (Thiele, 1906).

#### Liguus fasciatus laureani

Platt, 1949

http://species-id.net/wiki/Liguus_fasciatus_laureani

Liguus fasciatus laureani Platt 1949: 77, fig. 15.

##### Remarks.

Two specimens were found in the ZMB collection labelled as “paratypes”; they are accompanied with a typewritten label and originate from the McGinty collection (now in UF; Slapcinsky, pers. commun.). The taxon name is one of the many manuscript names of Carlos de la Torre, most of which have been used by Clench, that may be found in several museums (González Guillén, pers. commun.). This taxon was published by Platt in a paper that does not fullfil the requirements of ICZN Art. 8, 11 nor 13; it is thus a nomen nudum. The name has only been cited in species catalogues by subsequent authors (Jaume 1952, Clench 1965, Breure and Schouten 1985).

#### Bulinus laurentii

Sowerby I, 1833

http://species-id.net/wiki/Bulinus_laurentii

Figs 14A, 14i

Bulinus laurentii Sowerby I 1833: 37.

##### Type locality.

“in Peruviâ”; see remarks.

**Label.** “Peru Callao”, in Albers’ handwriting.

##### Dimensions.

“long. 0.55, lat. 0.3 poll. [H 13.9, D 7.6 mm]”; figured specimen herein H 15.3, D 7.9, W 6.5.

##### Type material.

ZMB 117770, one probable syntype; ex Albers coll. No. 10, ex Cuming coll.

##### Remarks.

The specimen has been received through Albers from the Cuming collection, and there is little doubt about its type status. For a clarification why type material described by G.B. Sowerby I found its way to the Cuming collection, see Breure and Ablett (2011: 3, 10, and 12). The type locality was restricted by Ramírez (2004: 41) to “Depto Lima, Las Salinas de Huacho, perto [sic, puerto] de Huacho”, which seems contradicting to the locality on the label found. The current systematic position has been taken from the same author.

##### Current systematic position.

Bulimulidae, *Bostryx modestus* (Broderip in Broderip and Sowerby I, 1832).

#### Bulimus (Odontostomus) leptodon

Martens, 1875

http://species-id.net/wiki/Bulimus_leptodon

Figs 33A–B, 33i

Bulimus (Odontostomus) leptodon Martens 1875: 276.

##### Type locality.

“Cordova im Binnenland des argentinischen Staates”.

##### Label.

“Cordova”, with a second, taxon label also in Martens’ handwriting.

##### Dimensions.

“Long. 21 1/2, diam. 7 (...) Mill.”; figured specimen herein H 22.2, D 7.36, W 9.1.

##### Type material.

ZMB 24077, two possible syntypes; ex Dohrn.

##### Remarks.

The taxon was described by Martens based on material from Stelzner. The current systematic position is after the last reviser, Parodiz (1942).

##### Current systematic position.

Odontostomidae, *Spixia alvarezii* (d’Orbigny, 1837).

#### Bulimus livescens

Pfeiffer, 1842

http://species-id.net/wiki/Bulimus_livescens

Figs 17E, 17iii

Bulimus livescens Pfeiffer 1842: 48; Pfeiffer 1848: 175.

##### Type locality.

“Mexico”.

##### Label.

“Mexico”, in Albers’ handwriting.

**Dimensions.** “Long. 22, diam. 10 mill.”; figured specimen herein H 22.5, D 9.7, W 7.0.

##### Type material.

ZMB 117771, one probable syntype; ex Albers coll. No. 528, Hegewisch leg.

##### Remarks.

Pfeiffer (1842) mentioned “Hegewisch in litt.”, which points to Hegewisch as the collector of the material described by Pfeiffer. As Albers had the same source, and the dimensions closely match those given by Pfeiffer (given as “Long. 23, diam. 9 mill.” in Pfeiffer 1848), there is little doubt about the type status. The shell is not fully grown. The current systematic position is after Thompson (2011).

##### Current systematic position.

Bulimulidae, *Drymaeus (Mesembrinus) livescens* (Pfeiffer, 1842).

#### Orthalicus lividus

Martens, 1864

http://species-id.net/wiki/Orthalicus_lividus

Figs 24A–B, 24ii

Orthalicus lividus Martens 1864: 542; Strebel and Pfeffer 1882: 30, pl. 11 fig. 18; Pilsbry 1899: 124, pl. 19 figs 18–19.
Orthalichus [sic] *lividus*; Martens 1893 [1890–1901]: 186, pl. 11 figs 11–11a.

##### Type locality.

“Am Vulkan Jorullo und in der Provinz Mechoacan [sic], Uhde”.

##### Label.

“Jorullo Vulkan / Prov. Michoacan”, with a second, taxon label in Martens’ handwriting [ZMB 4458]. “Michoacan” [ZMB 4461, 4594].

##### Dimensions.

“Long. 79, diamet. 43 (...) Millim.”; figured specimen herein H 79.4, D 42.3, W 7.2.

##### Type material.

ZMB 4458, one syntype; Uhde leg. ZMB 4461, three syntypes; Uhde leg. ZMB 4594, two (juvenile) syntypes.

##### Remarks.

Martens did not specify on how many specimens his description was based. The specimen ZMB 4458 is broken in the last whorl due to the thiness of the shell; it corresponds to the figures of Strebel and Pfeffer (1882) and Martens 1893 [1890–1901]. The specimens ZMB 4461 are accompanied by labels referring to Strebel and Pfeffer’s (1882) “Form A” and “Form B”. The current systematic position is after Thompson (2011).

##### Current systematic position.

Orthalicidae, *Orthalicus lividus* Martens, 1864.

#### Bulimus loxensis

Pfeiffer, 1846

http://species-id.net/wiki/Bulimus_loxensis

Figs 17F–G, 17vi

Bulimus loxensis Pfeiffer 1846: 85; Breure 1979: 121 (lectotype designation).
Drymaeus (Mesembrinus) loxensis ; Breure and Eskens 1981: 77. pl. 7 fig. 6.

##### Type locality.

[Ecuador, Prov. Loja] “El Catamaija prope Loxa reipublica Aequatoris. (Hartweg in coll. Cuming)”.

##### Label.

“Republ. equador Loxa”, in Albers’ handwriting.

##### Dimensions.

“Long. 35, diam. 14 mill.”; figured specimen herein H 24.7, D 11.5, W 5.7.

##### Type material.

ZMB 117772, one syntype; ex Albers coll. No. 131, ex Cuming coll.

##### Remarks.

The material originates from the Cuming collection and has the locality nearly in the same wording; the type status of this specimen is not disputed herein. Further type material is in the NHMUK collection (Breure and Ablett, in preparation).

##### Current systematic position.

Bulimulidae, *Drymaeus (Mesembrinus) loxensis* (Pfeiffer, 1846).

#### Bulimus lugubris

Dunker, 1882

http://species-id.net/wiki/Bulimus_lugubris

Figs 8A–C, 8ii

Bulimus lugubris Dunker 1882: 378, pl. 11 figs 1–2.
Plekocheilus lugubris ; Pilsbry 1895 [1895–1896]: 68, pl. 37 figs 95–96.

##### Type locality.

[Colombia] “Prope Pasto Columbiae australis”.

##### Label.

“Santiago 2100 M.”.

##### Dimensions.

“Long. testae 51; ejuaque latit. 28 mill.”; figured specimen herein H 50.4, D 33.1, W 4+.

##### Type material.

ZMB 117760, one probable syntype; ex Dunker coll.

##### Remarks.

The locality on the label is more specific than the published type locality; “Santiago” possibly refers to a locality SW Pasto, just north of Tulcán across the Colmbian-Ecuadorian border. The top of the shell is slightly damaged, but it agrees otherwise well with Dunker’s figures. His figures shows the vertical sculpture too pronounced and may thus be misleading. The shell identified as *Plekocheilus (Eurytus) lugubris* by Borrero and Breure (2011: 49, figs 15K–M) does not correspond to this species, but is a hitherto unknown species. However, their reclassification of Dunker’s taxon with *Plekocheilus (Eurytus)*is supported.

##### Current systematic position.

Amphibulimidae, *Plekocheilus (Eurytus) lugubris* (Dunker, 1882).

#### Bulimus macgillivrayi

Pfeiffer, 1855

http://species-id.net/wiki/Bulimus_macgillivrayi\according_to_Breure_2013

Figs 10B, 10i

Bulimus macgillivrayi Pfeiffer 1855b: 108, pl. 32 fig. 2.
Placostylus (Placocharis) macgillivrayi ; Breure and Schouten 1985: 63 (lectotype designation).
Placocharis macgillivrayi ; Delsaerdt 2010: 61, pl. 10 figs 9–11; Breure and Ablett 2012: 25, figs 14B, 14v.

##### Type locality.

“Wanderer Bay, Guadalcanar, Salomon’s Islands (*Macgillivray*)”.

##### Label.

“Solomons Inseln”, in Albers’ handwriting.

##### Dimensions.

“Long. 59, diam. 22 mill.”; specimen figured herein H 51.3, D 21.3, W 6.2.

##### Type material.

ZMB 117763, one paralectotype; ex Albers coll. No. 383, ex Cuming coll.

##### Remarks.

Pfeiffer did not state on how many specimens his description was based. The specimen was received from Cuming by Albers and is here considered type material; the lectotype is in NHMUK. The current systematic position follows Delsaerdt (2010).

##### Current systematic position.

Bothriembryontidae, *Placocharis macgillivrayi* (Pfeiffer, 1855).

#### Orthalichus maclurae

Martens, 1893

http://species-id.net/wiki/Orthalichus_maclurae

Figs 23A–C, 23i

Orthalichus [sic] *maclurae*Martens 1893 [1890–1901]: 188, pl. 11 figs 1–3.
Orthalicus maclurae ; Pilsbry 1899: 125, pl. 21 figs 40–42.
Zebra maclurae ; Strebel 1909: 69, pl. 13 figs 193, 198.

##### Type locality.

“N.W. Nicaragua: Cacao, in the Bay of Fonseca, on trees of the yellow-wood, *Maclura aurantiaca*, fam. Moreae (*Capt. Joh. Sch*ä*ffer*)”.

##### Label.

“Cacao, Bay v. Fonseca / Nicaragua Capt. Joh. Schäffer / Borcherding”, in Martens’ handwriting.

##### Dimensions.

“Long. 52, diam. 29 (...) millim.”; largest figured specimen herein H 49.0, D 29.6, W 6.2.

##### Type material.

ZMB 48202, four syntypes. ZMB 109889, five (juvenile) syntypes.

##### Remarks.

Martens (1893 [1890–1901]) wrote “Owing to the kindness of Fr. Borcherding, Vegesack, Bremen, I have before me twenty-two specimens (...)”. Of the three specimens ZMB 48202a, one correspond to Martens “var. a”, and one to “var. b” (pl. 11 fig. 2); ZMB 48202b is one specimen, corresponding to pl. 11 fig. 1 (“var. c”). According to the register book these specimens have been acquired by exchange, but it is not known with whom or which institution. The current systematic position is following Thompson (2011).

##### Current systematic position.

Orthalicidae, *Orthalicus maclurae* Martens, 1893.

#### Zebra maculiferus

Strebel, 1909

http://species-id.net/wiki/Zebra_maculiferus

Figs 21G–H, 21iv

Orthalichus [sic] *princeps*; Martens 1893 [1890–1901]: pl. 10 fig. 6.
Zebra maculiferus Strebel 1909: 30, pl. 3 fig. 38.

##### Type locality.

“Costarica”.

##### Label.

“Costarica”, with a second, taxon label in Strebel’s handwriting.

##### Dimensions.

“38,7 × (21,1)25,5 [H × (Dmin)Dmax in mm]”; figured specimen herein H 38.7, D 23.9, W 6.0.

##### Type material.

ZMB 34609, holotype; Jordan leg.

##### Remarks.

Strebel described this taxon on a shell, also figured by Martens, from Costa Rica. The current systematic position follows Thompson (2011), but may need revisionary work.

##### Current systematic position.

Orthalicidae, *Orthalicus maculiferus* (Strebel, 1909).

#### Placostylus (Placocharis) manni

W.F. Clapp, 1923

http://species-id.net/wiki/Placostylus_manni

Figs 10A, 10ii

Placostylus (Placocharis) manni W.F. Clapp 1923: 411, pl. 5 figs 1–2; Abbott 1989: 103.
Placocharis manni ; Delsaerdt 2010: 55, pl. 10 figs 1-5.

##### Type locality.

[Solomon Islands] “Auki, Malaita Id.”.

##### Label.

“Auki, Malaita, / Solomon Ids.”, in Clapp’s handwriting.

##### Dimensions.

“G.d. 41 mm. alt. 85 mm”; figured specimen herein H 81.5, D 37.5, W 5.9.

##### Type material.

ZMB 74852, one paratype; W.M. Mann leg.

##### Remarks.

The holotype is MCZ 32437, also figured by Abbott (1989).The current systematic position is following Delsaerdt (2010).

##### Current systematic position.

Bothriembryontidae, *Placocharis manni* (W.F. Clapp, 1923).

#### Placostylus miltocheilus manugiensis

B. Rensch, 1934

http://species-id.net/wiki/Placostylus_miltocheilus_manugiensis

Figs 9A, 9iv

Placostylus miltocheilus manugiensis B. Rensch 1934 in I. and B. Rensch 1934: 453; I. and B. Rensch 1935: 73, pl. 1 fig. 3.
Aspastus miltocheilus manugiensis ; Delsaerdt 2010: 24, pl. 1 figs 12–17.

##### Type locality.

[Solomon Islands] “Manugia auf Sanchristoval”.

##### Label.

“Manugia / San Christoval”, in Rensch’s handwriting.

##### Dimensions.

“Höhe 46.4–74.0 mm; Durchm. 20.9–29.3 mm”; figured specimen herein H 67.1, D 29.1, W 5.3.

##### Type material.

ZMB 78674, eleven and five paratypes; E. Paravicini leg.

##### Remarks.

According to Delsaerdt (2010) the holotype is NMB 3792a; the current systematic position is also after this author.

##### Current systematic position.

Bothriembryontidae, *Aspastus miltocheilus manugiensis* (B. Rensch, 1934).

#### Bulimus maranhonensis

Albers, 1854

http://species-id.net/wiki/Bulimus_maranhonensis

Figs 28C–E, 28ii

Bulimus maranhonensis Albers 1854: 216; Pfeiffer 1855 [1854–1860]: 42, pl. 11 figs 11–12.
Orthalicus maranhonensis ; Pilsbry 1899: 198, pl. 41 figs 3–4.

##### Type locality.

“in Columbia ad fluvium Maranhon”.

##### Label.

“Columbia / ad fluvium Maranhon”, in Albers’ handwriting.

##### Dimensions.

“Long. 76, diam. 30 mill.”; figured specimen herein H 75.6, D 36.8, W 6+.

##### Type material.

ZMB 101825, lectotype; ex Albers coll. No. 545. ZMB 111927, one (juvenile) paralectotype; ex Albers coll. No. 471, both Warszewicz leg. See remarks.

##### Remarks.

Albers did not state on how many specimens his description was based. The largest specimen found (ZMB 101825) has the top damaged and a label ‘545’ glued on the last whorl behind the lip. This specimen is here designated lectotype (**design. n.**) to define this still ill-understood taxon. One specimen from the Paetel collection (ZMB 101826) is labelled “Maranhon”, without further data; there is no evidence that this specimen has been part of the original series and therefore it is not considered type material. This taxon is tentatively retained as a valid species.

##### Current systematic position.

Orthalicidae, *Sultana (Metorthalicus) maranhonensis* (Albers, 1854).

#### Bulimus (Odontostomus) martensii

Doering, 1875

http://species-id.net/wiki/Bulimus_martensii

Figs 33C, 33ii

Bulimus (Odontostomus) martensii Doering 1875a: 181; Doering 1875b: 455; Neubert and Janssen 2004: 216, pl. 19 fig. 242.
Scalarinella (Spixia) martensii ; Zilch 1971: 201, pl. 12 fig. 19 (lectotype designation).
Spixia martensii ; Salas Oroño 2007: 9, figs 15–26.

##### Type locality.

[Argentina] “cerca de Totoral (Prov. de Córdova)”.

##### Label.

“Totoral (Argentinien)”, taxon label in Martens’ handwriting.

##### Dimensions.

“Long. 19 mm.; lat. 7 1/3 mm.”; figured specimen herein H 21.4, D 7.34, W 8.8.

##### Type material.

ZMB 28807, two paralectotypes; ex Doering.

##### Remarks.

The locality matches the published one; see Salas Oroño (2007) for a discussion of the distribution range and the type locality. Doering did not mention on how many specimens his description was based. The specimens were directly received from Doering and are here considered probable type material. The lectotype and one paralectotype are in SMF (Neubert and Janssen 2004). The current systematic position is after Salas Oroño (2007).

##### Current systematic position.

Odontostomidae, *Spixia martensii* (Doering, 1875).

#### Zebra miles

Strebel, 1909

http://species-id.net/wiki/Zebra_miles

Figs 24C–D, 24i

Zebra miles Strebel 1909: 64, pl. 12 figs 183–184, 186–189.

##### Type locality.

[Mexico] “Dos Arroyos, 25 miles NE. of Acapulco”.

##### Label.

“Dos Arroyos 25 miles NE / of Acapulco”, with a second, taxon label in Strebel’s handwriting.

##### Dimensions.

“70,8 × (32,8)39,3 [H × (Dmin)Dmax in mm]”; figured specimen herein H 71.0, D 38.1, W 7.1.

##### Type material.

ZMB 101830, four syntypes; H.H. Smith leg.

##### Remarks.

Strebel based the original description on eight specimens, of which four have been found in the ZMB collection. The specimen figured herein corresponds to Strebel (1909: pl. 12 fig. 186). The current systematic position is following Thompson (2011).

##### Current systematic position.

Orthalicidae, *Orthalicus ponderosus ponderosus* Strebel in Strebel and Pfeffer, 1882.

#### Bulimus monachus

Pfeiffer, 1857

http://species-id.net/wiki/Bulimus_monachus

Figs 16B, 16ii

Bulimus monachus Pfeiffer 1857b: 333; Pfeiffer 1869 [1866–1869]: 493, pl. 106 figs 9–10.
Drymaeus monachus ; Pilsbry 1898 [1897–1898]: 282, pl. 51 figs 22–23.

##### Type locality.

“Meobamba, Peru”.

##### Label.

“Mejobamba”, in Albers’ handwriting.

##### Dimensions.

“Long. 31, diam. 11 1/2 mill.”; figured specimen herein H 24.2, D 12.0, W 6.3.

##### Type material.

ZMB 117773, one syntype; ex Albers coll. No. 587, ex Cuming coll.

##### Remarks.

This taxon was described from the Cuming collection and the type status of this specimen is herein not disputed. Other type material is in the NHMUK collection (Breure and Ablett, in preparation). The wrinkled protoconch sculpture, together with the corneous colour of the shell, reveals this taxon—classified within *Drymaeus* so far—belongs to the genus *Bulimulus* (**comb. n.**).

##### Current systematic position.

Bulimulidae, *Bulimulus monachus* (Pfeiffer, 1857) (**comb. n.**).

#### Bulimulus (Bulimulus) monticola

Doering, 1879

http://species-id.net/wiki/Bulimulus_monticola

Figs 15F–G, 15v

Bulimulus (Bulimulus) monticola Doering 1879: 69.

##### Type locality.

[Argentina] “la Sierra de los Granadillos (Catamarca) y en la cuesta de Tocima (sierra de Famatina)”.

##### Label.

“Sierra de Uspallata [?]”, taxon label in Doering’s handwriting.

##### Dimensions.

“Long. 13–17mm; lat. 7–9mm”; figured specimen herein H 13.05, D 7.4, W 5.2.

##### Type material.

ZMB 34725, two syntypes; ex Doering.

##### Remarks.

Doering did not state on how many specimens he based his description, but the range in measurements indicates that it was more than one. Although the locality on the label cannot be deciphered completely, it is different from the published type locality. However, it is possible that this locality is more specific as Doering tended to give quite general areas as type locality (see under *conospira* Doering). The specimens were received directly from him and are considered syntypes. The current systematic position is according to Miquel (1995).

##### Current systematic position.

Bulimulidae, *Bostryx tortoranus* (Doering, 1879).

#### Bulimus moussoni

Pfeiffer, 1853

http://species-id.net/wiki/Bulimus_moussoni

Figs 17H, 17v

Bulimus moussoni Pfeiffer 1853: 147.

##### Type locality.

“St. Domingo”.

##### Label.

“Haiti”, label in Albers’ handwriting.

##### Dimensions.

“Long. 26, diam. 12 mill.”; figured specimen herein H 23.5, D 12.5, W 5.9.

##### Type material.

ZMB 117774, one syntype; ex Albers coll. No. 124, ex Cuming coll.

##### Remarks.

This taxon was described from shells collected by Sallé on Hispaniola (also sometimes named Hayti, Haiti or St. Domingo on old maps), and deposited in the Cuming collection. The type status of this specimen is not disputed. Other type material is present in the NHMUK collection (Breure and Ablett, in preparation).

##### Current systematic position.

Bulimulidae, *Drymaeus (Mesembrinus) moussoni* (Pfeiffer, 1853).

#### Bulimus (Odontostomus) multispiratus

Doering, 1877

http://species-id.net/wiki/Bulimus_multispiratus

Figs 33D, 33v

Bulimus (Odontostomus) multispiratus Doering 1877: 326; Doering 1878: 245; Neubert and Janssen 2004: 218, pl. 18 fig. 228.
Scalarinella (Spixia) multispirata ; Zilch 1971: 201, pl. 12 fig. 24 (lectotype designation).

##### Type locality.

[Argentina] “Pendiente Oeste de la Sierra de Aconjigasta (Quebrada de Yatan, de Nieve, Agua de los Oscuros)”.

##### Label.

“Sierra de Aconjigasta”, in Martens’ handwriting, a second taxon label in Doering’s handwriting.

##### Dimensions.

“Long. 16–19mm. lat. 4mm”; figured specimen herein H 17.4, D 4.38, W 11.0.

##### Type material.

ZMB 28509, one paralectotype; ex Doering.

##### Remarks.

The locality given on the label could not be found in modern gazetteers, but is likely in Prov. Córdoba. The specimen was directly received from Doering and is here considered probable type material. Doering did not mention the number of specimens his description was based upon, but the fact that he gave a range indicates that he had several specimens at hand. The lectotype and two other paralectotypes are in SMF (Neubert and Janssen 2004). The current systematic position is after Richardson (1993).

##### Current systematic position.

Odontostomidae, *Spixia multispirata* (Doering, 1877).

#### Zebra zoniferus naesiotes

Strebel, 1909

http://species-id.net/wiki/Zebra_zoniferus_naesiotes

Figs 25A–B, 25i

Zebra zoniferus naesiotes Strebel 1909: 53, pl. 9 figs 130–145.

##### Type locality.

[Venezuela] “Puerto Cabello (...) Inseln Barbados und Trinidad [see remarks] (...) Barbados”.

##### Label.

“Trinidad”, with a second, taxon label in Strebel’s handwriting.

##### Dimensions.

“42,4 × (22,5)27,3 [H × (Dmin)Dmax in mm]”; figured specimen herein H 53.4, D 32.6, W 6.0.

##### Type material.

ZMB 117785, six syntypes; ex Hamburg museum.

##### Remarks.

Strebel (1909) based the original description on material from different sources; the ZMB specimens were apparently part of the material that is mentioned by Strebel (1909: 7) as received from “Inseln Barbados und Trinidad” through the shell dealer Umlauff. In total Strebel listed 19 specimens in his paper. The specimen figured is the largest in the lot, in which the different colour forms distinguished by Strebel are represented. The current systematic position is according to own unpublished data.

##### Current systematic position.

Orthalicidae, *Orthalicus undatus* (Bruguière, 1789) (**syn. n.**).

#### Bulimus nasutus

Martens, 1885

http://species-id.net/wiki/Bulimus_nasutus

Bulimus nasutus Martens 1885: 191; Simone 2006: 171.

##### Type locality.

“Theophilo Ottoni in der brasilischen Provinz Minas Geraes”.

##### Label.

“Brasilien”.

##### Dimensions.

“Long. 30, diam. 9 (...) mm.”; specimen found H 24.6, D 9.41, W 7.6.

##### Material.

ZMB 109741, one specimen; ex Linnaea.

##### Remarks.

This material was labelled as holotype, but does not comply with the data given by Martens (1885), who mentioned Hollerbach as collector. It is not considered as type material. Simone (2006) refers to a figure of the ZMB material which is missing; the shell height mentioned by him is not matching the specimen found.

#### Orthalicus nobilis

Rolle, 1895

http://species-id.net/wiki/Orthalicus_nobilis

Figs 19F–G, 19iv

Orthalicus nobilis Rolle 1895: 131.

##### Type locality.

[Mexico] “Colima”.

##### Label.

“Colima / Mex”, in Martens’ handwriting.

##### Dimensions.

“Alt. 59, diam. 29 (...) mm.”; figured specimen herein H 58.8, D 29.2, W 5+.

##### Type material.

ZMB 47656, lectotype; ex Rolle.

##### Remarks.

Rolle did not state on how many specimens his description was based. The specimen, of which the top is damaged, corresponds to Rolle’s measurements; the shell is here designated as lectotype (**design. n.**) to define this poorly known taxon. The classification of Thompson (2011) of this taxon as a valid species is tentatively retained.

##### Current systematic position.

Orthalicidae, *Orthalicus nobilis* Rolle, 1895.

#### Scutalus (Scutalus) ortizpuentei

Weyrauch, 1967

http://species-id.net/wiki/Scutalus_ortizpuentei

Scutalus (Scutalus) ortizpuentei Weyrauch 1967: 378, pl. 7 fig. 100; Breure 2012: 11, pl. 5 figs 52–54.

##### Type locality.

“Norte de Perú, vertiente occidental de la Cordillera Occidental, valle de Chancay, entre Chiclayo y Chota (80 km al norte de Quinden)”.

##### Label.

“Cascas bei Trujillo, 1400 m, leg. W. Weyrauch”, typewritten label by Weyrauch; see remarks.

##### Material.

ZMB 110778, one specimen; W.K. Weyrauch leg.

##### Remarks.

According to the typewritten label this specimen was identified by Weyrauch as *Scutalus latecolumellaris ortizpuentei* and distributed as “Paratypoide”. The locality does not match the type locality and—since the taxon was based on a single specimen—no other localities were mentioned in the text. Moreover, the text stated that the holotype was collected by J. Ortiz de la Puente. Therefore, we must conclude that this specimen is not a paratype of this taxon. Presumably, Weyrauch collected this material during his Peruvian time in the 1940s or 1950s (Barbosa et al. 2008), identified it and distributed it as paratype well before he described the taxon in 1967 (see also Breure and Neubert 2008). The holotype is FML 1234 (re-figured by Breure 2012).

#### Placostylus (Charis) paeteli

Kobelt, 1890

http://species-id.net/wiki/Placostylus_paeteli

Figs 13A, 13i–ii

Placostylus (Charis) paeteli Kobelt 1890 [1890–1891]: 65, pl. 16 figs 1–2.
Placostylus paeteli ; Pilsbry 1900: 102, pl. 43 figs 11–13.

##### Type locality.

[Fiji] “Viti-Inseln”.

##### Label.

“Viti Insl”.

##### Dimensions.

“Alt. 72, diam. 35 Mm.”; figured specimen herein H74.0, D34.5, W5.9.

##### Type material.

ZMB 101819, holotype; ex Paetel coll.

##### Remarks.

Kobelt wrote “Das abgebildete Stück liegt in der Paetel’schen Sammlung im Berliner Museum”, thus implying that he had only one specimen at hand; it is thus the holotype. The material is accompanied by a label “*Bulimus / moussoni /* Gräffe”. The latter refers to a manuscript name in Museum Godeffroy (not *Bulimus moussoni* Pfeiffer, 1853); Crosse (1875: 13) has noted this homonymy and proposed the name *Bulimus (Placostylus) graeffei*. Kobelt, however, stated that his taxon was different.

##### Current systematic position.

Bothriembryontidae, *Callistocharis paeteli* (Kobelt, 1890).

#### Porphyrobaphe (Myiorthalicus) dennisoni pallida

Strebel, 1909

http://species-id.net/wiki/Porphyrobaphe_dennisoni_pallida

Figs 18G–H, 18iv

Porphyrobaphe (Myiorthalicus) dennisoni pallida Strebel 1909: 115, pl. 21 fig. 328, pl. 24 figs 376–379.

##### Type locality.

[Colombia] “Cauca-Tal, Columbien (...) Frontino, 6–8000´”.

**Label.** “Frontina / Neu Grenada”, with a second, taxon label in Strebel’s handwriting.

##### Dimensions.

See remarks; figured specimen herein H 55.0, D 33.8, W 5.8.

##### Type material.

ZMB 117782, one probable syntype; ex Dunker ex Schmeltz [18]74, [?G. Wallis leg., see remarks].

##### Remarks.

Strebel (1909: 116–117) listed in total six lots and mentioned “Nach dem mir vorliegenden Material aus der O. Semperschen und meiner alten Sammlung, dass wir beiden z. Z. [zur Zeit] von Schmeltz, der den Vertrieb des Wallisschen Material hatte, erwarben, ist die *var. pallida* mit der Etikette Frontino, Neu-Granada, versehen”. This sentence makes clear that lot 2 and 3 (seven specimens in total) in Strebel’s list refers to this material, of which he figured two and gave measurements of four specimens respectively. The shell height in his material (totally more than 15 specimens mentioned) varies between 34.5–83.3 mm. The taxon label in Strebel’s hand makes it very probable that this specimen belonged to the original series. The provisional current systematic position is according to own unpublished data.

##### Current systematic position.

Orthalicidae, *Hemibulimus dennisoni* (Reeve, 1848).

#### Helix (Cochlodina) pantagruelina

Moricand, 1833

http://species-id.net/wiki/Helix_pantagruelina

Figs 30F, 30iii

Helix (Cochlodina) pantagruelina Moricand 1833: 542, pl. 1 fig. 7.
Odontostomus pantagruelinus ; Pilsbry 1901 [1901–1902]: 63, pl. 8 fig. 85.

##### Type locality.

“le Brésil”.

##### Label.

“Bahia”, in Albers’ handwriting.

##### Dimensions.

“Long. 6 cent. 5 mill.”; figured specimen herein H 54.3, D 20.2, W 8.6.

##### Type material.

ZMB 117779, one syntype; ex Albers coll. No. 413, ex Moricand.

##### Remarks.

Moricand did not state on how many specimens his description was based. The specimen was received from him by Albers; the type status is not disputed herein. The current systematic position is after Simone (2006).

##### Current systematic position.

Odontostomidae, *Burringtonia pantagruelina* (Moricand, 1833).

#### Placostylus paravicinianus

B. Rensch, 1934

http://species-id.net/wiki/Placostylus_paravicinianus

Figs 12B, 12iv

Placostylus paravicinianus B. Rensch in I. and B. Rensch 1934: 451; I. and B. Rensch 1935: 79, pl. 1 fig. 7.
Aspastus paravicinianus ; Delsaerdt 2010: 66, pl. 11 figs 7–11.

##### Type locality.

[Solomon Islands] “Guadalcanar”.

##### Label.

“Aola, Guadelcanar”, in Rensch’s handwriting.

##### Dimensions.

“Höhe 60.5–71.4 mm; Durchm. 28.4–32.2 mm”; figured specimen herein H 66.6, D 28.3, W 6.2.

##### Type material.

ZMB 78711, two paratypes; E. Paravicini leg.

##### Remarks.

According to Delsaerdt (2010) the holotype is NMB 3798a; the current systematic position is also after this author. Rensch and Rensch (1935) mentioned having 10 specimens (“Es liegen 10 Schalen...vor”), whereas Delsaerdt mentioned one holotype and 10 paratypes in the NMB collection; the status of the Berlin specimens is not disputed since the label reads “Paratypen!” in the handwriting of Rensch.

##### Current systematic position.

Bothriembryontidae, *Aspastus paravicinianus* (B. Rensch, 1934).

#### Placostylus miltocheilus paravicinii

B. Rensch, 1934

http://species-id.net/wiki/Placostylus_miltocheilus_paravicinii

Figs 9B, 9ii

Placostylus miltocheilus paravicinii B. Rensch in I. and B. Rensch 1934: 453; I. and B. Rensch 1935: 73, pl. 1 fig. 4.
Aspastus miltocheilus paravicinii ; Delsaerdt 2010: 22, pl. 1 figs 2–6.

##### Type locality.

[Solomon Islands] “Wai Beroni, Sanchristoval”.

##### Label.

“Wai Beroni / San Christoval”, in Rensch’s handwriting.

##### Dimensions.

“Höhe 52.5–58.7 mm; Durchm. 19.8–22.4 mm”; figured specimen herein H 55.9, D 19.6, W 5.7.

##### Type material.

ZMB 78795, nine paratypes; E. Paravicini leg.

##### Remarks.

According to Delsaerdt (2010) the holotype is NMB 3798a; the current systematic position is also after this author.

##### Current systematic position.

Bothriembryontidae, *Aspastus miltocheilus paravicinii* (B. Rensch, 1934).

#### Bulimulus (Scutalus) peristomatus

Doering, 1879

http://species-id.net/wiki/Bulimulus_peristomatus

Figs 15C, 15iii

Bulimulus (Scutalus) peristomatus Doering 1879: 66.

##### Type locality.

[Argentina] “la sierra de Pocho (Quebr. de Yatan, de Mermela, etc.)”.

##### Label.

“Sierra de Pocho (Cordoba)”, taxon label in Doering’s handwriting.

##### Dimensions.

“Long. 27–29mm; lat. 11–13 1/2mm”; figured specimen herein H 26.5, D 15.8, W 4+.

##### Type material.

ZMB 34723, one syntype; ex Doering.

##### Remarks.

Doering did not state on how many specimens he based his description, but the range in measurements indicates that it was more than one. The specimen was received directly from him and is considered a syntype. The current systematic position is according to Miquel (1993).

##### Current systematic position.

Bulimulidae, *Bostryx stelzneri* (Dohrn, 1875).

#### Placostylus (Eumecostylus) phenax

W.F. Clapp, 1923

http://species-id.net/wiki/Placostylus_phenax

Figs 9E, 9iii

Placostylus (Eumecostylus) phenax W.F. Clapp 1923: 412, pl. 5 figs 3–4.
Eumecostylus phenax ; Delsaerdt 2010: 40, pl. 5 figs 11–15.

##### Type locality.

[Solomon Islands] “Wainoni, San Christoval Id.”.

##### Label.

“Wainoni Bay / San Christoval /Solomon Ids.”, in Clapp’s handwriting.

##### Dimensions.

“G.d. 27 mm. alt. 82.5 mm”; figured specimen herein H 81.3, D 25.3, W 5+.

##### Type material.

ZMB 74851, one paratype; W.M. Mann leg.

##### Remarks.

The holotype is MCZ 32466. The current systematic position is after Delsaerdt (2010).

##### Current systematic position.

Bothriembryontidae, *Eumecostylus phenax* (W.F. Clapp, 1923).

#### Bulimus (Odontostomus) philipii

Doering, 1875

http://species-id.net/wiki/Bulimus_philipii

Figs 33E, 33iii

Bulimus (Odontostomus) philipii Doering 1875a: 180; Doering 1875b: 456; Neubert and Janssen 2004: 223, pl. 19 fig. 241.
Scalarinella (Spixia) philipii ; Zilch 1971: 201, pl. 12 fig. 21–22.

##### Type locality.

[Argentina, Prov. Córdoba] “cerca de Totoral”.

##### Label.

“Totoral (Argentinien)”, taxon label in Martens’ handwriting.

##### Dimensions.

“Long. 17–19mm; lat. 3 3/4–4 1/4mm”; figured specimen herein H 18.0, D 6.05, W 10.0.

##### Type material.

ZMB 28504, one syntype; ex Doering.

##### Remarks.

The locality matches the published type locality. The specimen was directly received from Doering and is here considered type material. There are 89 other syntypes in the SMF collection (Neubert and Janssen 2004). The current systematic position is after Richardson (1993).

##### Current systematic position.

Odontostomidae, *Spixia philipii* (Doering, 1875).

#### Bulimus (Eudioptus) psidii

Martens, 1877

http://species-id.net/wiki/Bulimus_psidii

Bulimus (Eudioptus) psidii Martens 1877: 351, pl. 12 fig. 6
Simpulopsis (Eudioptus) psidii ; Köhler 2007: 156.

##### Remarks.

Contrary to Köhler’s remark, Breure (1979) did not list this taxon under *Simpulopsis (Eudioptus)* since he did not consider *Bulimus (Eudioptus) psidii* belonging to the Bulimulidae (then Bulimulinae) (Breure, unpublished data). Upon examination of the type specimens in the ZMB it became clear that Martens’ taxon may be classified with *Platysuccinea* (Sagdidae). This has to be confirmed by further anatomical and phylogenetic studies.

#### Zebra delphinus pumilio

Strebel, 1909

http://species-id.net/wiki/Zebra_delphinus_pumilio

Figs 21C–D, 21i

Zebra delphinus pumilio Strebel 1909: 33, pl. 3 figs 43–44, 46, 48, pl. 4 fig. 51.

##### Type locality.

“Mazatlan”.

##### Label.

“Oaxaca / SW Mex”, with a second, taxon label in Strebel’s handwriting.

##### Dimensions.

“39,9 × (19,1)23,1 [H × (Dmin)Dmax in mm]”; figured specimen herein H 39.7, D 22.3, W 6.2.

##### Type material.

ZMB 101834, one syntype; ex Wallenberg coll., Höhe leg.

##### Remarks.

Strebel (1909: 33–34) described this taxon, which he regarded as a form of his variety *nebulosus*, from eight specimens; the shell indicated from the ZMB corresponds to Strebel’s plate 4 fig. 51.

##### Current systematic position.

Orthalicidae, *Orthalicus delphinus* (Strebel, 1909).

#### Orthalicus (Laeorthalicus) reginaeformis

Strebel, 1909

http://species-id.net/wiki/Orthalicus_reginaeformis

Figs 18C–D, 18ii

Orthalicus (Laeorthalicus) reginaeformis Strebel 1909: 180, pl. 22 figs 353a–c.
Sultana (Laeorthalicus) reginaeformis ; Zilch 1960: 513, fig. 1794.

##### Type locality.

[Brazil?] “Rio Branco”.

##### Label.

“Rio Branco”, with a second, taxon label in Strebel’s handwriting.

##### Dimensions.

“51,3 × (19,6)23,0 [H × (Dmin)Dmax in mm]”; figured specimen herein H 46.9, D 22.7, W 7.2.

##### Type material.

ZMB 101824, holotype; ex Staudinger.

##### Remarks.

Strebel had a single shell (“das sich im Berliner Museum (...) befindet”), thus this is the holotype although the shell height does not correspond to Strebel’s data; the shell, however, fits his figure very well. It is not known where Staudinger collected this shell, but Brazil is likely given the many localities ‘Rio Branco’ in gazetteers; cf. also Schieleyko (1999: 359), who attributes this species to a specimen found in Brazil, Edo. Amazonas, Tabatinga. His classification of *Laeorthalicus* as a separate genus is herein considered as erroneous; left- and right handed specimens may occur in the same population of Orthalicid species (Breure, unpublished data; Simone 2006: fig. 543), and the description of a separate subgenus for the single, somewhat subadult, sinistral shell by Strebel may be interpreted as premature.

This taxon is now placed within the genus *Corona*, which species list badly needs a revision; the current systematic position is therefore a provisional one.

##### Current systematic position.

Orthalicidae, *Corona perversa* (Swainson, 1821) (**comb. n., syn. n.**).

#### Bulimus (Odontostomus) reticulatus

Doering, 1877

http://species-id.net/wiki/Bulimus_reticulatus

Figs 33F, 33iv

Bulimus (Odontostomus) reticulatus Doering 1877: 331; Doering 1878: 250; Neubert and Janssen 2004: 227, pl. 18 fig. 229.
Scalarinella (Spixia) aconjigastana ; Zilch 1971: 202, pl. 12 fig. 29 (lectotype designation).

##### Type locality.

[Argentina, Prov. Córdoba] “la pendiente Este de la Sierra de Aconjigasta, los altos de la Tablada, Plumeria, etc.”.

##### Label.

“Sierra de Aconjigasta”, in Martens’ handwriting, a second taxon label in Doering’s handwriting.

##### Dimensions.

“Long. 17–18mm; lat. 5mm”; figured specimen herein H 18.8, D 5.29, W 9.1.

##### Type material.

ZMB 28502, two paralectotypes; ex Doering.

##### Remarks.

The locality given on the label could not be found in modern gazetteers, but is likely in Prov. Córdoba. The specimens were directly received from Doering and are herein considered type material. Doering did not mention the number of specimens his description was based upon, but the fact that he gave a range indicates he had several specimens at hand. The lectotype and four other paralectotypes are in SMF (Neubert and Janssen 2004).

##### Current systematic position.

Odontostomidae, *Spixia reticulata* (Doering, 1877).

#### Bulimus rhodacme

Pfeiffer, 1842

http://species-id.net/wiki/Bulimus_rhodacme

Figs 14B, 14ii

Bulimus rhodacme Pfeiffer 1842: 50.

##### Type locality.

“Huasco, Chile: prope urbem Frierina (Bridges, Cuming)”.

##### Label.

“Chili”, in Albers’ handwriting.

##### Dimensions.

“Long. 15, diam. 5 1/2 mill.”; figured specimen herein H 13.1, D 5.97, W 6.5.

##### Type material.

ZMB 117775, one syntype; ex Albers coll. No. 7, ex Cuming coll.

##### Remarks.

This taxon was described from the Cuming collection but Pfeiffer did not mention on how many specimens his description was based. Albers received this shell from Cuming, hence it type status is not disputed. No type material belonging to this species could be found in the NHMUK collection (Breure, unpublished data). This taxon is provisionally classified with *Bostryx* sensu lato (Breure 1979), for which a genus revision is overdue.

##### Current systematic position.

Bulimulidae, *Bostryx rhodacme* (Pfeiffer, 1842).

#### Bulimus rhodolarynx

Reeve, 1849

http://species-id.net/wiki/Bulimus_rhodolarynx

Figs 14F, 14iii

Bulimus rhodolarynx Reeve 1849 [1848–1850]: pl. 72 fig. 518; Breure 1979: 58.
Bostryx rhodolarynx rhodolarynx ; Breure 1978: 116 (lectotype designation).

##### Type locality.

“Banks of the Apurimac, Alto-Peru”.

##### Label.

“Andes Peruviae”, in Albers’ handwriting; see remarks.

##### Dimensions.

Not given; figured specimen herein H 27.0, D 16.1, W 6.3.

##### Type material.

ZMB 117776, one paralectotype; ex Albers coll. No. 1144, ex Cuming coll.

##### Remarks.

For an explication of the relation between material described by Reeve and the Cuming collection, see Breure and Ablett (2011: 10, 12). Since Albers received the specimen from Cuming, its type status is not disputed herein. It may be noted that the lectotype material in the NHMUK collection is also labelled “Andes of Peru” (Breure 1978); the published type locality is thus more detailed than the one found with the type material. The current systematic position is following Breure (1978), but see remarks under the previous taxon.

##### Current systematic position.

Bulimulidae, *Bostryx rhodolarynx rhodolarynx* (Reeve, 1849).

#### Zebra richardsoni

Strebel, 1909

http://species-id.net/wiki/Zebra_richardsoni

Figs 23F–G, 23iii

Zebra richardsoni Strebel 1909: 36, pl. 4 figs 60–62.

##### Type locality.

“Tepic, N.-W. Mexiko”.

##### Label.

“Tepic / NW Mex”, with a second, taxon label in Strebel’s handwriting.

##### Dimensions.

“47,0 × (23,3)27,6 [H × (Dmin)Dmax in mm]”; figured specimen herein H 47.1, D 26.6, W 6.3.

##### Type material.

ZMB 101831, three syntypes; W. Richardson leg.

##### Remarks.

Strebel described this taxon from three specimens; “Die Stücke gehören dem Berliner Museum”. The shell figured herein from the ZMB collection corresponds to Strebel’s plate 4 fig. 61. Another shell in the lot corresponds to his fig. 60. The current systematic position is after Thompson (2011).

##### Current systematic position.

Orthalicidae, *Orthalicus richardsoni* (Strebel, 1909).

#### Bulimus ringens

Dunker in Dunker et al., 1847

http://species-id.net/wiki/Bulimus_ringens

Figs 30C–E, 30ii

Bulimus ringens Dunker in Dunker et al. 1847: 83.

##### Type locality.

“Brasilien”; see remarks.

##### Label.

“Macahe, Bras. / Beschke”; in Dunker’s handwriting.

##### Dimensions.

“Long. 18, diam. 5 1/3 mm”; figured specimen H 18.6, D 5.66, W 6.6.

##### Type material.

ZMB 117780, one syntype; Beschke leg.

##### Remarks.

Dunker did not mention on how many specimens his description was based upon. However, he mentioned “(Rarissimus teste Beschke)” which reveals that Beschke was the collector. The material is accompanied by a label that mentions this name, therefore eliminating any doubt about the type status of this specimen. The label also reveals a more specific locality than published by Dunker; “Macahe” could be Macaé in Edo. Rio de Janeiro. The current systematic position is after Simone (2006).

##### Current systematic position.

Odontostomidae, *Bahiensis ringens* (Dunker in Dunker and Pfeiffer, 1847).

#### Plagiodontes rocae

Doering, 1881

http://species-id.net/wiki/Plagiodontes_rocae

Figs 32B, 32ii

Plagiodontes rocae Doering 1881: 65, pl. 1 figs 5–6.

##### Type locality.

[Argentina, Prov. Buenos Aires] “Sierra de Currumalan”.

**Label.** “Sierra de Currumalan”, with a second taxon label in Martens’ handwriting.

##### Dimensions.

“Long. 21-24mm; lat. 8-9mm”; figured specimen H 23.7, D 9.71, W 8.0.

##### Type material.

ZMB 34728, two syntypes; ex Doering.

##### Remarks.

Doering (1881: 67) gave measurements for four specimens. The specimens in ZMB were received directly from Doering and there is no doubt about their type status.

##### Current systematic position.

Odontostomidae, *Plagiodontes rocae* Doering, 1881.

#### Bulimus (Odontostomus) salinicola

Doering, 1877

http://species-id.net/wiki/Bulimus_salinicola

Figs 33G, 33vi

Bulimus (Odontostomus) salinicola Doering 1877: 328; Doering 1878: 247; Neubert and Janssen 2004: 228, pl. 18 fig. 230.
Scalarinella (Spixia) salinicola ; Zilch 1971: 203, pl. 12 fig. 31 (lectotype designation).

##### Type locality.

[Argentina, Prov. Córdoba] “al pié de la pendiente Oeste de la S. de Aconjigasta (Dep. Chancaní)”.

##### Label.

“Sierra de Aconjigasta”, in Martens’ handwriting, a second taxon label in Doering’s handwriting.

##### Dimensions.

“Long. 22mm; lat. 6mm”; figured specimen herein H 20.4, D 6.21, W 7+.

##### Type material.

ZMB 28506, two paralectotypes; ex Doering.

##### Remarks.

The locality given on the label could not be found in modern gazetteers, but is likely in Prov. Córdoba. The specimens were directly received from Doering and are herein considered type material. The lectotype and three other paralectotypes are in SMF (Neubert and Janssen 2004).

##### Current systematic position.

Odontostomidae, *Spixia salinicola* (Doering, 1877).

#### Bulimus scarabus

Albers, 1854

http://species-id.net/wiki/Bulimus_scarabus

Figs 12A, 12i–iii

Bulimus scarabus Albers 1854: 219.
Placostylus scarabus ; Neubert et al. 2009: 88, figs 131–146.

##### Type locality.

“Nova Caledonia”.

##### Label.

“Nova Caledonia”, in Albers’ handwriting.

##### Dimensions.

“Long. 60, diam. 30 mill.”; figured specimen herein H 53.8, D 38.7, W 6.0.

##### Type material.

ZMB 101820, one syntype, ex Petit; 101821, one syntype, ex Albert coll. No. 379.

##### Remarks.

The status of this taxon has recently been revised by Neubert et al. (2009). The specimen ex Cuming they mentioned could not be found in the ZMB collection.

##### Current systematic position.

Bothriembryontidae, *Placostylus scarabus* (Albers, 1854).

#### Zebra selectus

Strebel, 1909

http://species-id.net/wiki/Zebra_selectus

Figs 25C–D, 25ii

Zebra selectus Strebel 1909: 37, pl. 4 figs 54, 58, 63.

##### Type locality.

“Trinidad (...) Coban, Guatemala”.

##### Label.

“Trinidad”, with a second, taxon label in Strebel’s handwriting.

##### Dimensions.

“50,5 × (25,1)32,3 [H × (Dmin)Dmax in mm]”; figured specimen herein H 50.3, D 30.4, W 6.5.

##### Type material.

ZMB 25568, one syntype; ex Deutsche Malakozoologische Gesellschaft Tausch-Verein.

##### Remarks.

Strebel described this taxon from two specimens of very different localities. The shell in the ZMB collection corresponds to Strebel’s figures 54 and 58. The current systematic position only reflects the status of this shell, as the Guatemala specimen was part of Strebel’s own collection and may have been lost. Thompson (2011) expressed his doubts if this taxon may be included in the Central American malacofauna.

##### Current systematic position.

Orthalicidae, *Orthalicus undatus* (Bruguière, 1789).

#### Bulimus shuttleworthi

Albers, 1854

http://species-id.net/wiki/Bulimus_shuttleworthi

Figs 29A–B, 29i

Bulimus shuttleworthi Albers 1854: 216; Pfeiffer 1855 in Küster and Pfeiffer 1854–1860: 31, pl. 8 figs 14–15.
Orthalicus shuttleworthi ; Pilsbry 1899: 201, pl. 41 figs 1–2.
Orthalicus (Metorthalicus) shuttleworthi ; Strebel 1909: 157, pl. 30 fig. 437.

##### Type locality.

“in Columbia ad fluvium Maranhon”.

##### Label.

“Columbia ad fluvium Maranhon”, in Albers’ handwriting.

##### Dimensions.

“Long. 71, diam. 29 mill.”; figured specimen herein H 70.5, D 34.4, W 5.5+.

##### Type material.

ZMB 101827, two syntypes; ex Albers coll. No. 544, Warzewicz leg.

##### Remarks.

Albers did not state on how many specimens his description was based. The largest specimen found has the top damaged and a label ‘544’ glued on the last whorl behind the lip; moreover, the specimen has damage on the dorsal side of both (pen)ultimate whorls. The shells corresponds well to Pfeiffer’s figure, who stated “Aus der Albers’schen und meiner Sammlung”. One other lot (ZMB 210505, two specimens) is labelled “Oberes Maranhon”, from the same collector, but not originating from the Albers collection. Since there is no evidence that these specimens have been part of the original series and the locality does not match the published one, they are not considered as type material. This taxon is tentatively retained as a valid species.

##### Current systematic position.

Orthalicidae, *Sultana (Metorthalicus) shuttleworthi* (Albers, 1854).

#### Macrodontes simplex

Thiele, 1906

http://species-id.net/wiki/Macrodontes_simplex

Figs 31G–I, 31iii

Macrodontes simplex Thiele 1906: 70, fig. 1–1a; Zilch 1960: 508, fig. 1781; Schileyko 1999: 334, fig. 412.

##### Type locality.

[Brazil] “Serra dos Tapes (Rio grande do Sul)”.

##### Label.

“Serra dos Tapes / Rio gr. do Sul”, in Thiele’s handwriting.

##### Dimensions.

“Höhe 36 mm. Dicke 10 mm”; figured specimen herein H 34.9, D 10.6, W 6.6.

##### Type material.

ZMB 55781, holotype; von Königswald leg. ZMB 55782, paratype; Schlüter leg.

##### Remarks.

Thiele described this taxon from two specimens in the ZMB collection. The paratype specimen (ZMB 55782) bears the label “Rio Grande do Sul”. The current systematic position is modified after Simone (2006).

##### Current systematic position.

Odontostomidae, *Odontostomus simplex* (Thiele, 1906).

#### Zebra sphinx

Strebel, 1909

http://species-id.net/wiki/Zebra_sphinx

Figs 27A–B, 27i

Zebra sphinx Strebel 1909: 66, pl. 12 figs 181, 185.

##### Type locality.

“Tepic (...) Colima (...) West-Mexiko”.

##### Label.

“Tepic NW Mexico”, with a second, taxon label in Strebel’s handwriting.

##### Dimensions.

“50,5 × (25,1)32,3 [H × (Dmin)Dmax in mm]”; figured specimen herein H 50.3, D 30.4, W 6.5.

##### Type material.

ZMB 101832, one syntype; W. Richardon leg.

##### Remarks.

Strebel described this taxon from five specimens from the same general region. The shell in the ZMB collection corresponds to Strebel’s figures 181 and 185. The current systematic position follows Thompson (2011), who seems to have restricted the type locality to Mexico, Edo. Nayarit, Tepic (stating “known only from the type locality”).

##### Current systematic position.

Orthalicidae, *Orthalicus sphinx sphinx* (Strebel, 1909).

#### Bulimulus (Scutalus) stelzneri

Dohrn, 1875

http://species-id.net/wiki/Bulimulus_stelzneri

Figs 15A, 15i

Bulimulus (Scutalus) stelzneri Dohrn 1875: 202.

##### Type locality.

“republica Argentina: Cerro de Chepe”.

##### Label.

“Sierra de Catamarca”, in (Dohrn’s?) handwriting.

##### Dimensions.

“Long. 23–28, lat. 14–20 (...) mill.”; figured specimen herein H 27.9, D 16.8, W 6.0.

##### Type material.

ZMB 34734, one possible syntype; ex Doering.

##### Remarks.

Dohrn did not mention on how many specimens his description was based, but the range of measurements indicate that he had several specimens at hand. Moreover, he mentioned “Ich erhielt dieselbe in einigen Exemplaren von meinem Freunde Dr. A. Stelzner mit einer grösseren Suite argentinischer, von ihm gesammelter Land- und Süsswasser-Conchylien. Da die andere unbeschriebenen Arten Manuscriptnamen von Herrn Dr. Doering in Cordova führen, so steht wohl deren Publikation in Bälde zu erwarten” (Dohrn 1875: 203). These sentences make clear that Stelzner, Doering and Dohrn were in regular contact and transfers of material (with unpublished names) did occur. The type locality is likely Cerro de Chepes, Prov. La Rioja (teste Miquel 1993: 164), although there is also a Cerro Chepe in Prov. San Juan. Doering’s material has a very general locality that, however, falls within the distribution range of the species. It is therefore possible that Doering’s material was part of the original series and the specimen is thus considered a possible syntype. The current systematic position is after Miquel (1993).

##### Current systematic position.

Bulimulidae, *Bostryx stelzneri* (Dohrn, 1875).

#### Bulimus strangei

Pfeiffer, 1855

http://species-id.net/wiki/Bulimus_strangei\according_to_Breure_2013

Figs 10C, 10iii

Bulimus strangei Pfeiffer 1855a: 8.
Placocharis strangei ; Delsaerdt 2010: 73, pl. 12 figs 5–9 (lectotype designation); Breure and Ablett 2012: 40, figs 14D, 14iv.

##### Type locality.

[Solomon Islands, Simbo] “Eddystone Island, Australian Sea”.

**Label.** “Eddystone Island / Solomon Inzeln”, in Albers’ handwriting [ZMB 117764]. A second label “Eddingtone [sic] Isl.” in Dunker’s handwriting [ZMB 117765].

##### Dimensions.

“Long. 46, diam. 17 mill.”; figured specimen herein H 47.3, D 20.9, W 5.6.

##### Type material.

ZMB 117764, one paralectotype; ex Albers coll. No. 384, ex Cuming coll. ZMB 117765, two probable paralectotypes; ex Dunker coll., ex Cuming coll.

##### Remarks.

There is no doubt that the specimen from the Albers collection may be considered from the type series, despite the fact there is no label in Pfeiffer’s handwriting. The two specimens from the Dunker collection are very likely also from the same source and are considered probable type material. The lectotype is NHMUK 20100652 (see Breure and Ablett 2012), thus the ZMB material are paralectotypes.

##### Current systematic position.

Bothriembryontidae, *Placocharis strangei* (Pfeiffer, 1855).

#### Bulimus stutchbury

Pfeiffer, 1860

http://species-id.net/wiki/Bulimus_stutchbury

Figs 15D, 15iv

Bulimus stutchbury Pfeiffer 1860: 137, pl. 51 fig. 8.
Placostylus stutchbury ; Kobelt 1891 [1890–1891]: 135, pl. 32 fig. 8; Pilsbry 1900: 88, pl. 36 fig. 35.

##### Type locality.

“Erumanga, New Hebrides”; see remarks.

##### Label.

“Neu Caledonia”, in Dunker’s handwriting; see remarks.

##### Dimensions.

“Long. 53, diam. 11 mill.”; figured specimen herein H 47.4, D 23.6 W 5.1.

##### Type material.

ZMB 117766, one possible syntype; ex Dunker coll., ex Cuming coll.

##### Remarks.

**Figure 8.**
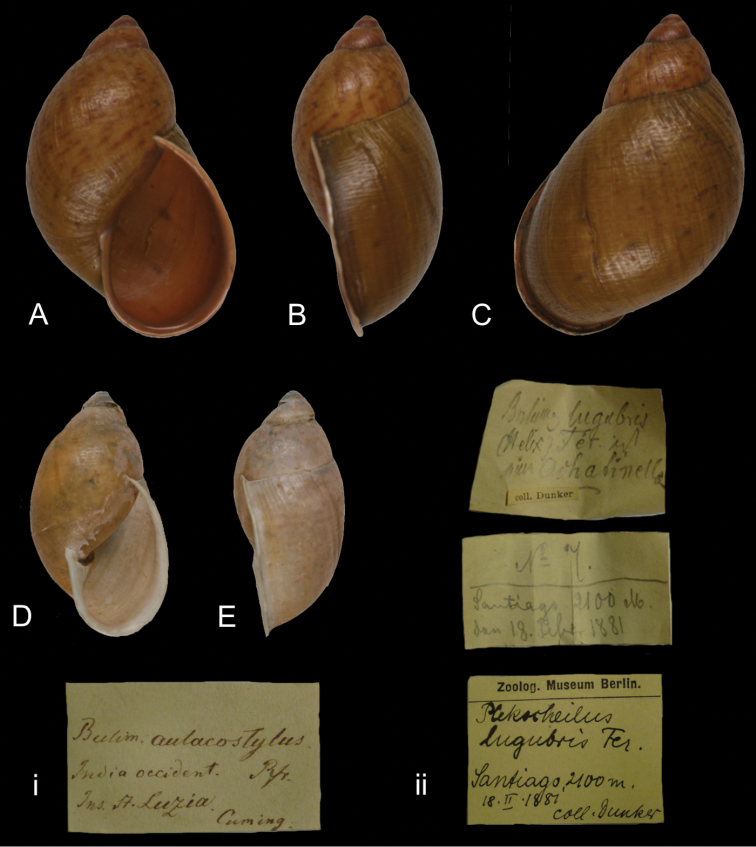
*Plekocheilus (Eurytus)* species **A–C, ii** *Plekocheilus (Eurytus) lugubris* (Dunker, 1882), probable syntype ZMB 117760 (H = 50.4) **D–E, i** *Plekocheilus (Eurytus) aulacostylus* (Pfeiffer, 1853), probable syntype ZMB 112723 (H = 37.6).

**Figure 9.**
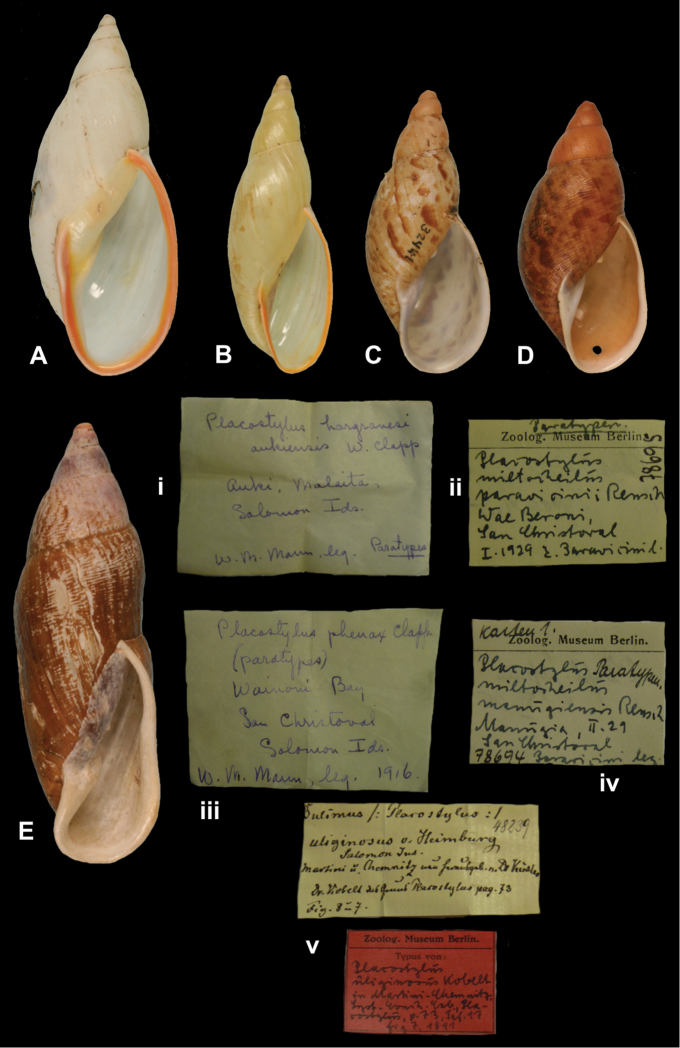
**A–B** *Aspastus* species **A, iv** *Aspastus miltocheilus manugiensis* (B. Rensch, 1934), paratype ZMB 78674 (H = 67.1) **B, ii** *Aspastus miltocheilus paravicinii* (B. Rensch, 1934), paratype ZMB 78795 (H = 55.9) **C–E** *Eumecostylus* species **C, i** *Eumecostylus hargravesi* (Cox, 1871), paratype of *Placostylus (Placocharis) hargravesi aukiensis* W.F. Clapp, 1923 ZMB 74853 (H = 51.1) **D, v** *Eumecostylus uliginosus* (Kobelt, 1891), syntype ZMB 48239 (H = 50.0) **E, iii** *Eumecostylus phenax* (W.F. Clapp, 1923), paratype ZMB 74851 (H = 81.3).

In Pfeiffer’s publication some mistakes have been made relating to the dimensions and figures. It seems likely that the dimensions of *Bulimus stutchbury* and the previous species (*Bulimus schomburgki*) have been exchanged. The dimensions given by Pfeiffer suggest a ratio of nearly 5:1 of shell height / diameter, which is clearly not according to his figure; the dimensions given for *Bulimus schomburgki* (“Long. 48, diam. 23 mill.”) better suit *Bulimus stutchbury*. The shell from the Dunker collection, which might have been erroneously labelled by him as he did not keep the original label after copying, closely matches Pfeiffer’s Pl. 51 fig. 8 except the parietal tubercle is not showing well in that figure. The legend of the plate assigns also fig. 9 to *Bulimus stutchbury*, but from the text on page 137 it is clear that this figure represents *Bulimus schomburgki*. It may be noted that the type of *Bulimus stutchbury* has not been found in the NHMUK collection.

Delsaerdt (2010: 69) has corrected the type locality to Solomon Islands, Russell Islands; this group of small island lies between New Georgia archipelago and Guadalcanar.

##### Current systematic position.

Bothriembryontidae, *Placocharis stutchbury* (Pfeiffer, 1860).

#### Bulimus (Dryptus) stuebeli

Martens, 1885

http://species-id.net/wiki/Bulimus_stuebeli

Bulimus (Dryptus) stuebeli Martens 1885: 172, pl. 32 figs 5–7 [sic, 6–8].
Dryptus stuebeli ; Köhler 2007: 129, fig. 14; Borrero and Breure 2011: 11.

##### Remarks.

Martens (1885: pl. 32 fig. 8) showed the sculpture on the last whorl, which appears upon inspection of the lectotype perfectly matching those of *Plekocheilus (Eurytus)* species. The lectotype lacks the top whorls and is otherwise quite worn. However, it has good locality data (Fusugasugá, 1700 m) and the taxon is here synonymized with *Bulimus (Eurytus) corticosus* Sowerby III, 1895 (**syn. n.**). The latter taxon strongly resembles *Bulimus stuebeli* morphologically, occurs in the same region, and has a similar altitudinal range (Borrero and Breure 2011: 26, figs 9C, 10D-G). As Martens’ taxon is a senior subjective synonym, the current systematic position of this taxon becomes *Plekocheilus (Eurytus) episcopalis stuebeli* (Martens, 1885) (**comb. n.**).

#### Bulimus terebralis

Pfeiffer, 1842

http://species-id.net/wiki/Bulimus_terebralis

Figs 14C–E, 14iv

Bulimus terebralis Pfeiffer 1842: 51.

##### Type locality.

“Coquimbo, Chile. (Bridges)”.

##### Label.

“Chili”, in Albers’ handwriting.

##### Dimensions.

“Long. 21, diam. 4 1/2 mill.”; figured specimen herein H 20.3, D 4.9, W 9.3.

##### Type material.

ZMB 117777, two probable syntypes; ex Albers coll. No. 17, ex Krantz, ex Cuming coll.

##### Remarks.

The locality data do not entirely match the published data, but there is no doubt that this material came from the Cuming collection via the German dealer Krantz (see introduction). The type status of the specimens is not disputed; type material of this taxon has not been found in the NHMUK collection (Breure, unpublished data). This taxon is provisionally classified with *Bostryx* sensu lato (Breure 1979), for which genus a revision is overdue.

##### Current systematic position.

Bulimulidae, *Bostryx terebralis* (Pfeiffer, 1842).

#### Bulimulus (Bulimulus) tortoranus

Doering, 1879

http://species-id.net/wiki/Bulimulus_tortoranus

Figs 15D–E, 15iv

Bulimulus (Bulimulus) tortoranus Doering 1879: 71.

##### Type locality.

[Argentina] “la sierra de Pocho ([Quebradas de] Totoras, Yatan, Cerro Salado, &.)”.

##### Label.

“Sierra de Cordoba”, taxon label in Doering’s handwriting.

##### Dimensions.

“Long. 26–27mm; lat. 10mm”; figured specimen herein H 21.9, D 8.93, W 6.0.

##### Type material.

ZMB 34718, three syntypes; ex Doering.

##### Remarks.

Doering did not mentioned on how many specimens his description was based. The specimens were directly received from Doering. The locality on the label is not exact, but the published type locality falls within the area indicated (cf. Miquel 1995: 123). The current systematic position follows Miquel (1995).

##### Current systematic position.

Bulimulidae, *Bostryx tortoranus* (Doering, 1879).

#### Odontostomus (Cyclodontina) inflatus trahyrae

Jaeckel, 1950

http://species-id.net/wiki/Odontostomus_inflatus_trahyrae

Figs 30I–K 2D

Odontostomus (Cyclodontina) inflatus trahyrae Jaeckel 1950: 131, fig. 1.
Plagiodontes trahyrae ; Simone 2006: 168, fig. 584.

##### Type locality.

“Insel Trauhyra, Brasilien”; see remarks.

##### Label.

“Insel Trahyra, / Brasilien”; in Jaeckel’s handwriting.

##### Dimensions.

“H. 12.0, Br. 7.5 mm”; figured specimen H 12.16, D 7.26 W 6.6.

##### Type material.

ZMB 95737, holotype; 96038, eight paratypes; all Schmierer leg., see remarks.

##### Remarks.

According to Jaeckel “eine Insel Trahyra [sic] zwar unbekannt sei, aber zwei Inseln mit dem Nahmen Trauhyra in Brasilien legen, beiden Flußinseln, und zwar eine im Fluß Japurá (Staat Amazonas) und die andere im Rio San Francisco (Staat Bahia)” [“Trahyra Island is unknown in Brazil, but there are two islands named Trauhyra, both river islands, one in Río Japurá (Edo. Amazonas) und the second one in Río San Francisco (Edo. Bahia)”]. Given the classification within *Cyclodontina*, which also occurs around Bahia, the latter locality may be more probable; this is also the interpration of Simone (2006). It may be noted that both the NGA and the Fuzzy gazetteers had no matches for “Trauhyra”. Furthermore, Jaeckel stated that his description was based upon “Holotypus und 9 Paratypoide”; only eight paratypes were found. The assignment of this taxon to *Plagiodontes* by Simone (2006) is erroneous; tentatively this taxon is retained in *Cyclodontina*.

##### Current systematic position.

Odontostomidae, *Cyclodontina trahyrae* (Jaeckel, 1950).

#### Bulinus translucens

Broderip in Broderip and Sowerby I, 1832

http://species-id.net/wiki/Bulinus_translucens

Figs 17I, 17iv

Bulinus translucens Broderip in Broderip and Sowerby I 1832a: 31; Sowerby I 1833 in Sowerby I and II 1832–1841: *Bulinus*, fig. 11.
Drymaeus translucens ; Pilsbry 1899: 89. pl. 24 fig. 28.

##### Type locality.

“in America meridionali, Kings and Saboga Islands, Bay of Panama”.

##### Label.

“in insulis ismus panamensis / Sobogon [sic] & Kings Island”, in Albers’ handwriting.

##### Dimensions.

“long. 7/8, lat. 4/8 poll. [H 22.2, D 12.7 mm]”; figured specimen herein H 13.3, D 8.62, W 4.8.

##### Type material.

ZMB 117778, one probable syntype; ex Albers coll. No. 125, ex Krantz, ex Cuming coll.

##### Remarks.

This specimen came from the Cuming collection via the German dealer Krantz (see Introduction) and has locality data matching the published one. The shell is not fully grown. Further type material is present in the NHMUK collection (Breure and Ablett, in preparation). The current systematic position is according to Thompson (2011).

##### Current systematic position.

Bulimulidae, *Drymaeus (Mesembrinus) translucens translucens* (Broderip in Broderip and Sowerby I, 1832).

#### Orthalichus tricinctus

Martens, 1893

http://species-id.net/wiki/Orthalichus_tricinctus

Figs 22E–F, 22iii

Orthalichus tricinctus Martens 1893 [1890–1901]: 185, pl. 11 fig. 8.

##### Type locality.

Various localities from Nicaragua to Peru; see remarks.

##### Label.

“Bois de Térraba / 250 m”, in Pittier’s handwriting; ”Terraba, Costarica / Pittier”, in Martens’ handwriting [ZMB 101828]. “Costarica / Pittier” [ZMB 109890]. “Vijagual / Costarica / Pittier”, in Pittier’s handwriting [ZMB 117786]. “Costarica Carmiol” [ZMB 117787].

##### Dimensions.

Not given; figured specimen herein H 48.1, D 30.1, W 5.9.

##### Type material.

ZMB 101828, lectotype and two paralectotypes; Pittier leg. ZMB 109890, two (juvenile) paralectotypes; Pittier leg. ZMB 117786, four paralectotypes; Pittier leg. ZMB 117787, one (juvenile) paralectotype; ex Carmiol.

##### Remarks.

Martens introduced this name for a variety of references and figures from previous authors, resulting in 13 different localities mentioned, mainly from Central America. The type locality is now restricted to Costa Rica, Prov. Puntarenas, Térraba (**restrict. n.**). In total ten specimens have been found in the ZMB collection which are referred to this taxon and may be traced in Martens (1893 [1890–1901]); one specimen corresponds to his figure and is here selected lectotype (**design. n.**). The current systematic position follows Thompson (2011).

##### Current systematic position.

Orthalicidae, *Orthalicus ferussaci tricinctus* Martens, 1893.

#### Bulimus (Odontostomus) tudiculatus

Martens, 1868

http://species-id.net/wiki/Bulimus_tudiculatus

Figs 30G–H, 30iv

Bulimus (Odontostomus) tudiculatus Martens 1868: 178.
Cyclodontina tudiculata ; Simone 2006: 168, fig. 583A.

##### Type locality.

[Brazil, Edo. Rio Grande do Sul] “Rödersberg” [Sao Leopoldo].

##### Label. 

“Rödersberg / S. Brasil”, with a second, taxon label also in Martens’ handwriting.

##### Dimensions.

“Long. 24, diam. 6 1/2 (...) Mill.”; figured specimen herein H 21.5, D 6.38, W 7+.

##### Type material.

ZMB 14543, three syntypes; R. Hensel leg.

##### Remarks.

Martens mentioned “drei mir vorliegenden Exemplaren”, corresponding with the three specimens found in the ZMB collection. The largest specimen, of which the top is damaged, is here figured; it probably would have corresponded to the measurements given by Martens if it had remained undamaged. The current systematic position is after Simone (2006).

##### Current systematic position.

Odontostomidae, *Cyclodontina tudiculata* (Martens, 1868).

#### Bulimus (Odontostomus) tumulorum

Doering, 1875

http://species-id.net/wiki/Bulimus_tumulorum

Figs 33H, 33vii

Bulimus (Odontostomus) tumulorum Doering 1875a: 187; Doering 1875b: 456; Neubert and Janssen 2004: 233, pl. 19 fig. 237.
Scalarinella (Spixia) tumulorum ; Zilch 1971: 203, pl. 12 fig. 32 (lectotype designation).

##### Type locality.

[Argentina] “la pendiente Oeste de la Sierra de Córdova”.

##### Label.

“Argentina”, taxon label in Doering’s handwriting.

##### Dimensions.

“Long. 12–13 mm; diam. 3 2/3–4 mm.”; figured specimen herein H 13.6, D 4.17, W 9.6.

##### Type material.

ZMB 25739, two paralectotypes; ex Dohrn ex Doering.

##### Remarks.

The locality given on the label does not match the published type locality. However, the taxon label in Doering’s handwriting leaves little doubt about the type status, as the specimens were received via Dohrn from Doering. The specimens are considered herein as paralectotypes. The lectotype and 24 other paralectotypes are in SMF (Neubert and Janssen 2004).

##### Current systematic position.

Odontostomidae, *Spixia tumulorum* (Doering, 1875).

#### Zebra maclurae turrita

Strebel, 1909

http://species-id.net/wiki/Zebra_maclurae_turrita

Figs 23D–E, 23ii

Orthalichus [sic] *melanochilus*; Martens 1893 [1890–1901]: 190, pl. 11 fig. 6 [figure only]
Zebra maclurae turrita Strebel 1909: 71, pl. 13 figs 204–205.

##### Type locality.

[Mexico] “Tehuantepec”.

##### Label.

No locality given, with a second, taxon label in Strebel’s handwriting.

##### Dimensions.

“59,0 × (29,5)35,1 [H × (Dmin)Dmax in mm]”; figured specimen herein H 56.2, D 31.2, W 6.6.

##### Type material.

ZMB 101829, one syntype; ex Rolle.

##### Remarks.

Strebel described this form from three specimens, of which one is housed in the ZMB collection. The specimen has labels both in Martens’ and Strebel’s handwriting, and corresponds to the figured specimen by Martens. The current systematic position is following Thompson (2011). Note that *Zebra maclurae turrita* Strebel, 1909 is a junior homonym of *Zebra quagga turrita* Strebel, 1909, and a senior homonym of *Zebra sphinx turrita* Strebel, 1909.

##### Current systematic position.

Orthalicidae, *Orthalicus maclurae* Martens, 1893.

#### Zebra sphinx turrita

Strebel, 1909

http://species-id.net/wiki/Zebra_sphinx_turrita

Figs 27C–D, 27ii

Zebra sphinx turrita Strebel 1909: 68, pl. 12 fig. 180.

##### Type locality.

“Tres Marias (...) Insel gegenüber San Blas, W.-Mexiko”.

##### Label.

“Tres Marias”, with a second, taxon label in Strebel’s handwriting.

##### Dimensions.

“56,9 × (25,5)30,5 [H × (Dmin)Dmax in mm]”; figured specimen herein H 56.9, D 28.7, W 6.7.

##### Type material.

ZMB 117784, holotype; Forrer leg.

##### Remarks.

Strebel described this taxon from a single specimen in the “Berl. Museum”. The shell corresponds to Strebel’s figure 180, and is regarded as holotype. The current systematic position follows Thompson (2011), who recognizes this taxon as subspecifically distinct from *Orthalicus sphinx sphinx* (Strebel, 1909). He also noticed that Strebel had introduced the name *turrita* three times under *Zebra*, but did not take any nomenclatorial actions. As the name [*Zebra sphinx*] *turrita* is preceded twice in Strebel’s work and is a junior homonym of *Zebra quagga turrita* Strebel, 1909 (Strebel 1909: 42), I here introduce the replacement name *tresmariae* Breure nom. n.

**Current systematic position.** Orthalicidae, *Orthalicus sphinx tresmariae* (**nom. n.**).

#### Orthalichus livens uhdeanus

Martens, 1893

http://species-id.net/wiki/Orthalichus_livens_uhdeanus

Figs 26A–D, 26i

Orthalicus livens Shuttleworth; Martens 1865: 38; Strebel and Pfeffer 1882: 32, pl. 11 fig. 19.
Orthalichus livens uhdeanus Martens 1893 [1890–1901]: 189.

##### Type locality.

“W. Mexico: West coast, State of Michoacan (*Uhde*)”.

##### Label.

“Mechoacan” [ZMB 4595], “West Küste / v. Mexico”, in Martens’ handwriting [ZMB 4596].

##### Dimensions.

Not given; figured specimen herein H 54.3, D 27.7, W 6+.

##### Type material.

ZMB 4595, one syntype. ZMB 4596, one syntype; both Uhde leg.

##### Remarks.

Martens (1865) mentioned the two specimens of Uhde in the ZMB collection under the name *Orthalicus livens* Shuttleworth,1856. ZMB 4596 corresponds to the specimen figured by Strebel and Pfeffer (1882); the top of the specimen is damaged. The specimen ZMB 4595 is accompanied by a label in Strebel’s handwriting “?livens / ?form / aberrans / Strebel”; in Strebel (1909: 43–44), however, there is no mention of this specimen. The current systematic position is after Thompson (2011).

##### Current systematic position.

Orthalicidae, *Orthalicus uhdeanus* Martens, 1893.

#### Placostylus (Charis) uliginosus

‘von Heimburg’ Kobelt, 1890

http://species-id.net/wiki/Placostylus_uliginosus

Figs 9D, 9v

Placostylus (Charis) uliginosus ‘von Heimb.’ Kobelt 1890 [1890–1891]: 73, pl. 17 figs 6–7.

##### Type locality.

“Salomonen”; see remarks.

##### Label.

“Salomon Ins.”, taxon label in Kobelt’s handwriting.

##### Dimensions.

Alt. 50, diam. 23 Mm.”; figured specimen herein H 50.0, D 24.2, W 5.2.

##### Type material.

ZMB 48239, one syntype; von Heimburg leg.

##### Remarks.

Kobelt did not state on how many specimens his description was based. However, the specimen corresponds to the dimensions given by him and his Pl. 17 fig. 7; his plate was published in 1890, the text in 1891 (Coan et al. 2012b). Delsaerdt (2010: 49) has restricted the type locality to Mara Masike.

##### Current systematic position.

Bothriembryontidae, *Eumecostylus uliginosus* (Kobelt, 1890).

#### Orthalicus varius

Martens, 1873

http://species-id.net/wiki/Orthalicus_varius

Figs 26E–F, 26ii

Orthalicus varius Martens 1873: 190, pl. 1 fig. 4.
Zebra varius ; Strebel 1909: 91–92.

##### Type locality.

[Venezuela] “Caracas (...) Angostura”.

##### Label.

“Caracas” [ZMB 21256], “Angostura” [ZMB 101835]; partly with labels in Albers’ and Martens’ handwriting.

##### Dimensions.

“Länge höchstens bis 51 Millim., Breite kaum die Hälfte derselben”; figured specimen herein H 47.2, D 23.7, W 6.9.

##### Type material.

ZMB 21256, two syntypes; ex Ernst. ZMB 101835a, c, eight syntypes, see remarks; ex Albers coll. Nos 49–55, Gruner leg.

##### Remarks.

Martens described this taxon on the basis of ten specimens; two young specimens from Ernst, and eigth specimens from the Albers collection and collected by Gruner. Lot ZMB 101835 has been split into four different ones. The specimens figured in Martens 1873 (: pl. 1 figs 4a–b) are ZMB 101835a; this lot contains two shells. ZMB 101835b contains five specimens with a taxon label in Martens’ handwriting, ex Albers collection Nos 56–61, and collected by Gruner. ZMB 101835c has the same data; these six shells had the numbers 49–55 in Albers’ collection. Finally, ZMB 101835d has three shells with identical data, and labelled in the Albers collection 62–64. The total number of shells in lot ZMB 101835 is thus 16 and larger than Martens stated (Martens 1873: 190). We now assume that Martens—for whatever reasons—did not consider the eight specimens in ZMB 101835b and 101835d when he described this taxon, and exclude these from the type material. The current systematic position follows Richardson (1993).

##### Current systematic position.

Orthalicidae, *Orthalicus varius* Martens, 1873.

#### Placostylus sanchristovalensis vicinus

B. Rensch, 1934

http://species-id.net/wiki/Placostylus_sanchristovalensis_vicinus

Figs 12C, 12v

Placostylus sanchristovalensis vicinus B. Rensch in I. and B. Rensch 1934: 452; I. and B. Rensch 1935: 76, pl. 1 fig. 5.
Eumecostylus vicinus ; Delsaerdt 2010: 42, pl. 6 figs 1–9.

##### Type locality.

[Solomon Islands] “Guadalcanar. Aola”.

##### Label.

“warscheinlich Guadalcanar” “Aola, Guadalcanar”, both in Rensch’s handwriting.

##### Dimensions.

“Höhe 58.2–61.2 mm; Durchm. 23.0–28.4 mm”; figured specimen herein H 63.6, D 27.5, W 5.7.

##### Type material.

ZMB 78702, one paratype; 78703, one paratype; both E. Paravicini leg.

##### Remarks.

Rensch and Rensch (1935: 77) wrote “Es liegen 5 Exemplare von Aola auf Guadalcanar vor, 2 Stücke von Rotalu, und 1 ohne Fundort, das den anderen so ähnlich ist, dass es wohl auch als von diesen Gebieten stammend betrachtet werden kann”. The last specimen is ZMB 78702 (“warscheinlich Guadalcanar”); Delsaerdt (2010: 42) mentioned “NMB (3952a) 2 paratypes, ... Rotalu; (3952c) 5 paratypes ... Aola)”. However, the status of the Berlin specimens is not disputed since each label reads “Paratyp” in the handwriting of Rensch. The current systematic position is after Delsaerdt (2010).

##### Current systematic position.

Bothriembryontidae, *Eumecostylus vicinus* (B. Rensch, 1934).

#### Plagiodontes weyenberghii

Doering, 1877

http://species-id.net/wiki/Plagiodontes_weyenberghii

Figs 32C, 32iii

Plagiodontes weyenberghii Doering 1877: 322; Doering 1878: 241; Pizá and Cazzaniga 2012: 390, fig. 1.
Plagiodontes weyemberghii Doering 1877: 322; Doering 1878: 241 (emendation).
Scalarinella (Plagiodontes) weyemberghii ; Zilch 1971: 199, pl. 11 fig. 13 (lectotype designation).

##### Type locality.

[Argentina, Prov. Córdoba] “Sierra de Aconjigasta, en algunas quebradas hondas y húmedas, como en la del ‘Nieve’ y en la de ‘Mermela’ en la pendiente Oeste”; see remarks.

##### Label.

“Sierra de Aconjigasta”, with a second, taxon label also in Martens’ handwriting.

##### Dimensions.

“Long. 26–28mm; lat. 12–14mm”; figured specimen H 26.6, D 14.67, W 6.7.

##### Type material.

ZMB 28501, two paralectotypes; ex Doering.

##### Remarks.

Doering did not mention on how many specimens his description was based. Zilch (1971) mentioned five specimens, of which he chose one as lectotype. The specimens in ZMB were received directly from Doering and are considered paralectotypes. Using modern gazetteers, the name ‘Aconjigasta’ could not be found; however, Quebrada de Mermela is in Prov. Córdoba. The current systematic position follows Richardson (1993). The correct spelling follows Pilsbry (1901 [1901–1902]: 101) as pointed out by Pizá and Cazzaniga (2012: 405).

##### Current systematic position.

Odontostomidae, *Plagiodontes weyenberghii* (Doering, 1877).

#### Bulimus willi

Dohrn, 1883

http://species-id.net/wiki/Bulimus_willi

Figs 31A–C, 31i

Bulimus willi Dohrn 1883: 350, pl. 11 figs 5–6.

##### Type locality.

Not specifically given. From the title and introduction it may be concluded “östlichen Brasilien (...) Quellgebiet des Mucury”.

##### Label.

“Minas geraes / Ob. Mucury”, in Dohrn’s handwriting.

##### Dimensions.

“Long. 28–32, diam. 9–9 1/2 (...) mm”; figured specimen herein H 27.9, D 9.33, W 7.8.

##### Type material.

ZMB 36423, one syntype; ex Dohrn.

##### Remarks.

As Dohrn mentioned a range in his measurements, he must have based his description on several specimens. Only one shell is present in the ZMB collection. The current systematic position is after Simone (2006).

##### Current systematic position.

Odontostomidae, *Moricandia willi* (Dohrn, 1883).

#### Zebra hackeri xanthus

Strebel, 1909

http://species-id.net/wiki/Zebra_hackeri_xanthus

Figs 25E–F, 25iii

Zebra hackeri xanthus Strebel 1909: 51, pl. 7 fig. 108.

##### Type locality.

[Mexico] “Dos Arroyos bei Acapulco”.

##### Label.

“Dos Arryos / b. Acapulco”, with a second, taxon label in Strebel’s handwriting.

##### Dimensions.

“43,6 × (24,0)28,3 [H × (Dmin)Dmax in mm]”; figured specimen herein H 57.4, D 31.8, W 6.4.

##### Type material.

ZMB 101833, holotype; H.H. Smith leg.

##### Remarks.

Strebel described this taxon as a “heterom[orph].” and from “ein unausgewachsenes Stück”. The shell, corresponding to Strebel’s figure 108, is thus the holotype. It may be noted that the taxon name *xanthus* was introduced three times by Strebel (1909) for a “heteromorph”; the others were *Zebra princeps xanthus* (1909: 20) and *Zebra boucardi xanthus* (1909: 74). The current systematic position follows Thompson (2011).

##### Current systematic position.

Orthalicidae, *Orthalicus hackeri* (Strebel, 1909).

## Plates

**Figure 10.**
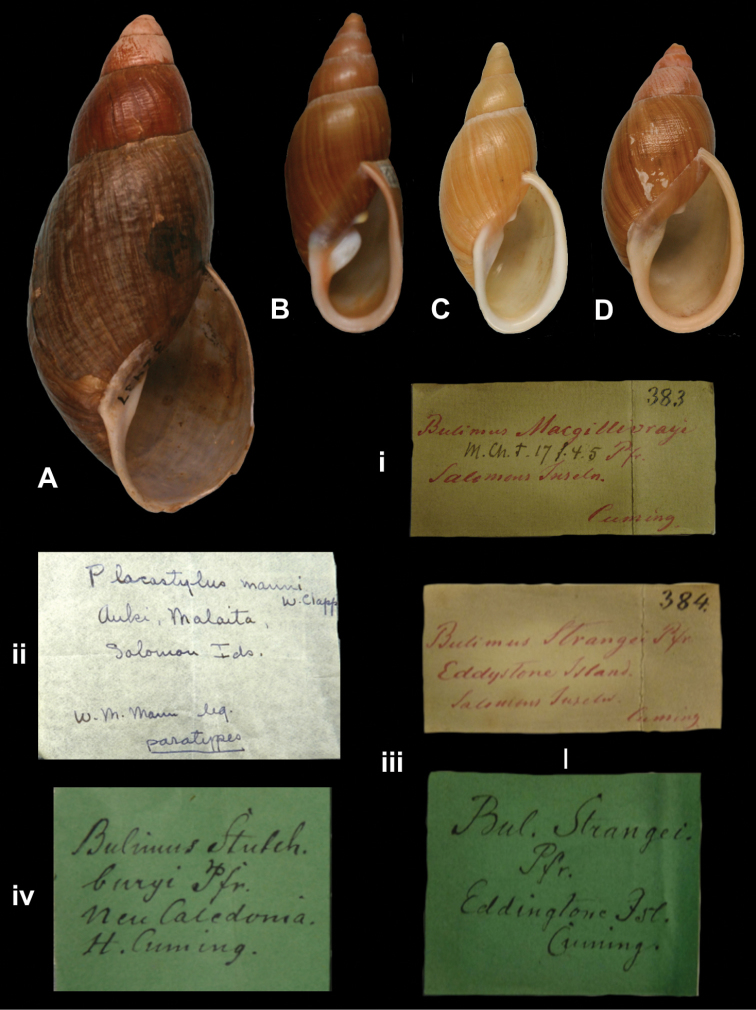
*Placocharis* species **A, ii** *Placocharis manni* (W.F. Clapp, 1923), paratype ZMB 74852 (H = 81.5) **B, i** *Placocharis macgillivrayi* (Pfeiffer, 1855) paralectotype ZMB 117763 (H = 51.3) Photo: C. Zorn **C, iii** *Placocharis strangei* (Pfeiffer, 1855) paralectotype ZMB 117764 (H = 47.3) **D, iv** *Placocharis stutchbury* (Pfeiffer, 1860) possible syntype ZMB 117766 (H = 47.4).

**Figure 11.**
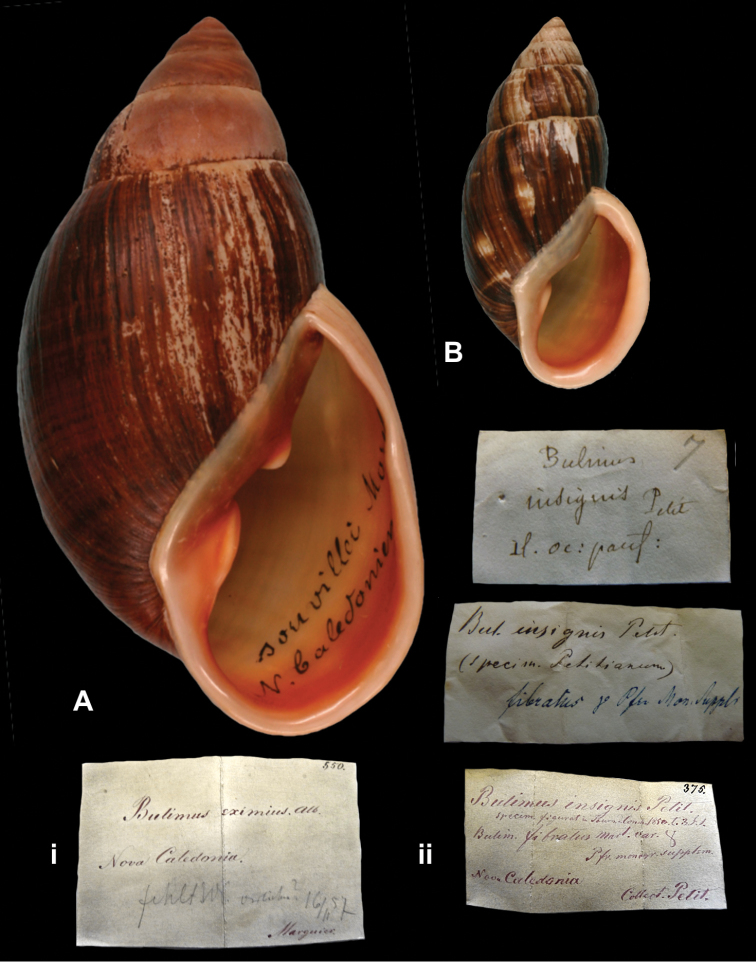
*Placostylus* species**A, i** *Placocharis fibratus souvillei* (Morelet, 1857), syntype of *Bulimus eximius* Albers, 1857 ZMB 117761 (H = 119.1) **B, ii** *Placocharis fibratus fibratus* (Martyn, 1784), syntype of *Bulimus insignis* Petit, 1850 ZMB 117762 (H = 61.9)

**Figure 12.**
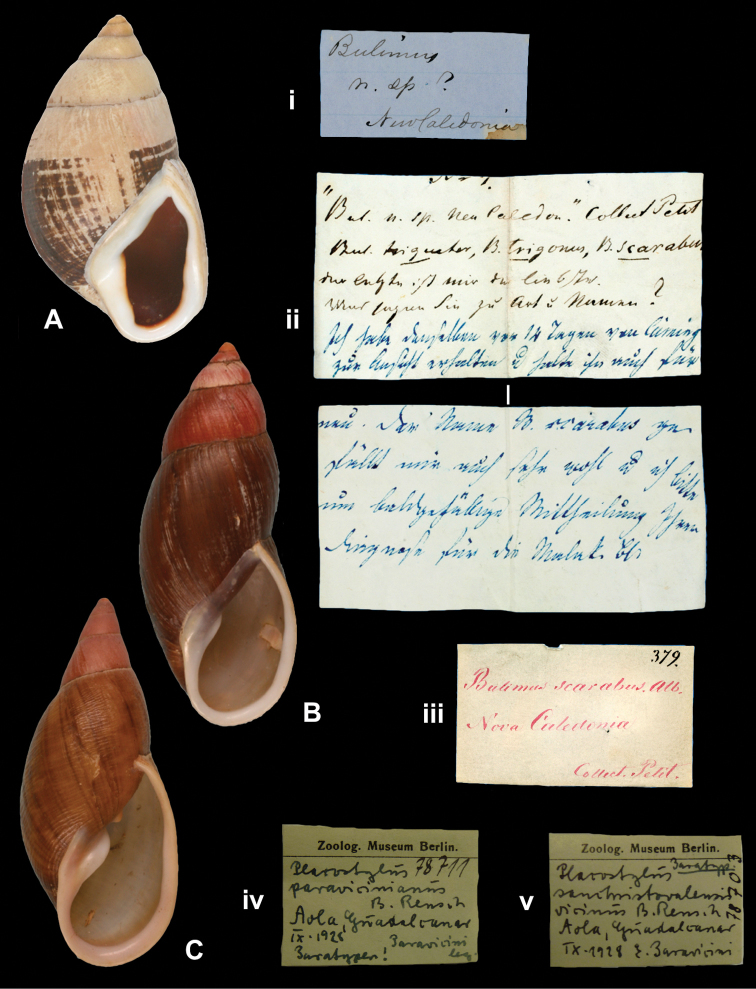
**A, i–iii** *Placostylus scarabus* (Albers, 1854), syntype ZMB 101820 (H = 53.8) Photo: C. Zorn **B, iv** *Aspastus paravicinianus* (B. Rensch, 1934), paratype ZMB 78711 (H = 66.6) **C, v** *Eumecostylus vicinus* (B. Rensch, 1934), paratype ZMB 78702 (H = 63.6).

**Figure 13.**
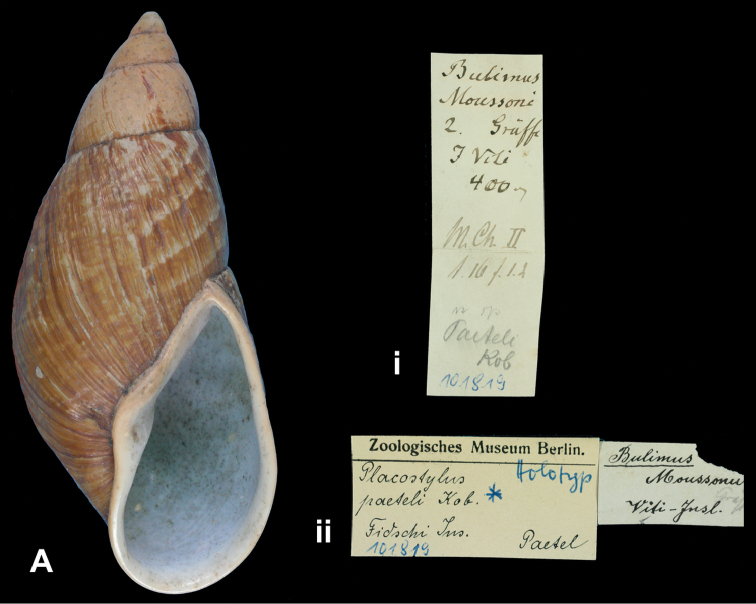
**A, i–ii** *Callistocharis paeteli* (Kobelt, 1890), holotype ZMB 101819 (H = 74.0) Photo: C. Zorn.

**Figure 14.**
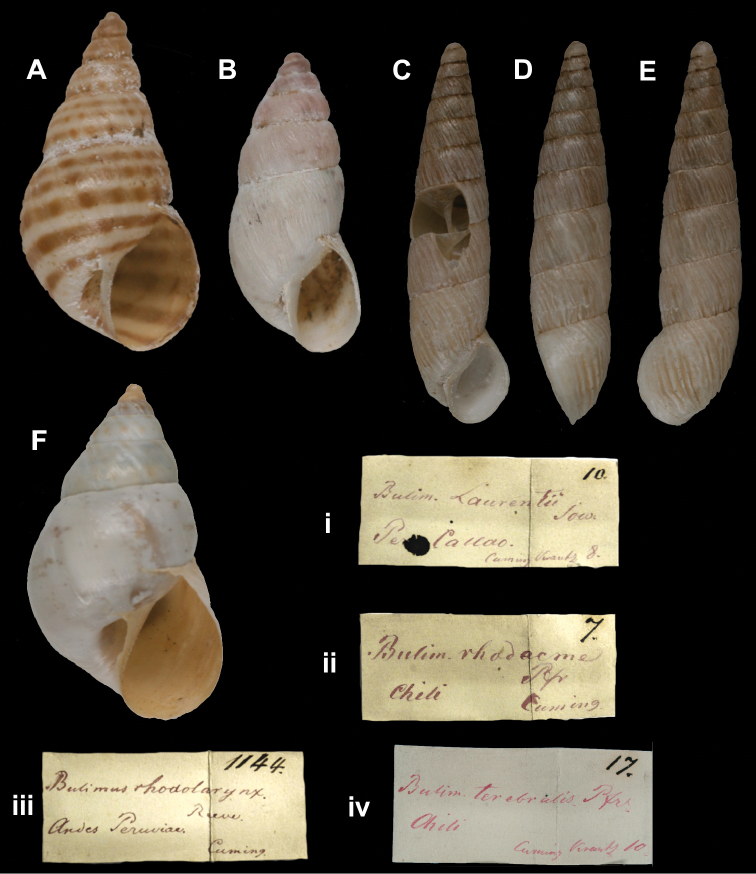
*Bostryx* (sensu lato) species **A, i** *Bostryx modestus* (Broderip in Broderip and Sowerby, 1832), probable syntype of *Bulimus laurentii* Sowerby I, 1833 ZMB 117770 (H = 15.3) **B, ii** *Bostryx rhodacme* (Pfeiffer, 1842), syntype ZMB 117775 (H = 13.1) **C–E, iv** *Bostryx terebralis* (Pfeiffer, 1842), probable syntype ZMB 117777 (H = 20.3) **F, iii** *Bostryx rhodolarynx* (Reeve, 1849), paralectotype ZMB 117776(H = 27.0). All enlarged.

**Figure 15.**
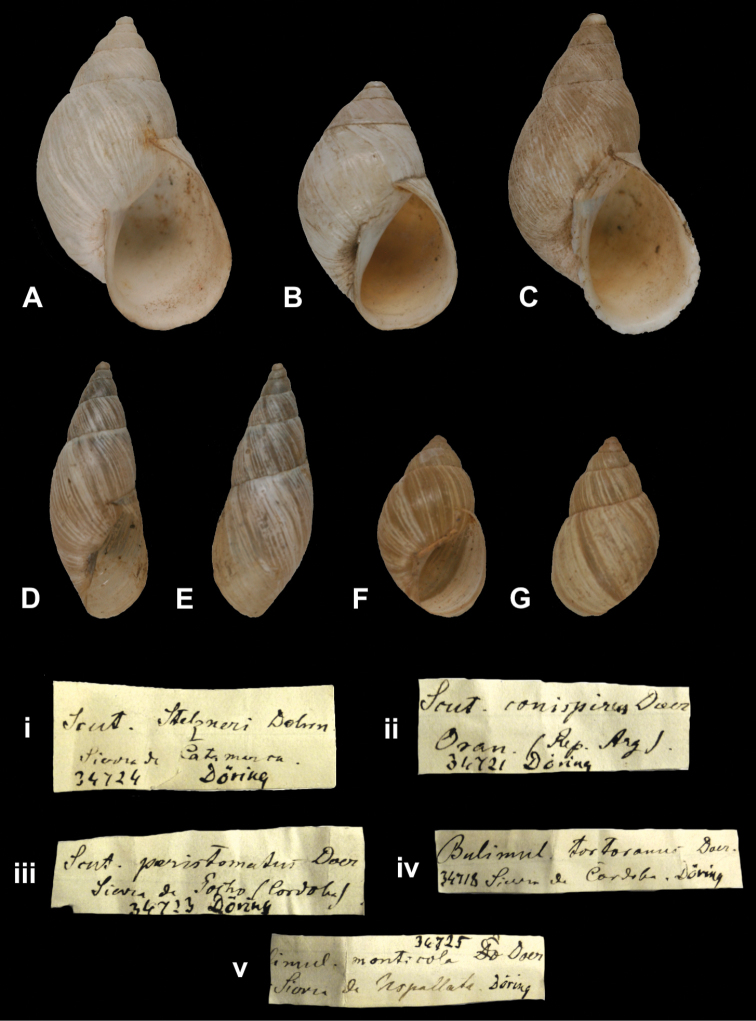
*Bostryx* (sensu lato) species **A–C** *Bostryx stelzneri* (Dohrn, 1875) **A, i** possible syntype of *Bulimulus (Scutalus) stelzneri* Dohrn, 1875 ZMB 34734 (H = 27.9) **B, ii** syntype of *Bulimulus (Scutalus) conospirus* Doering, 1879 ZMB 34721 (H = 21.9) **C, iii** syntype of *Bulimulus (Scutalus) peristomatus* Doering, 1879 ZMB 34723 (H = 26.5) **D–E, iv** *Bostryx tortoranus* (Doering, 1879) syntype ZMB 34718 (H = 21.9) **F–G, v** *Bostryx tortoranus* (Doering, 1879), syntype of *Bulimulus (Bulimulus) monticola* Doering, 1879 ZMB 34725 (H = 13.05).All enlarged.

**Figure 16.**
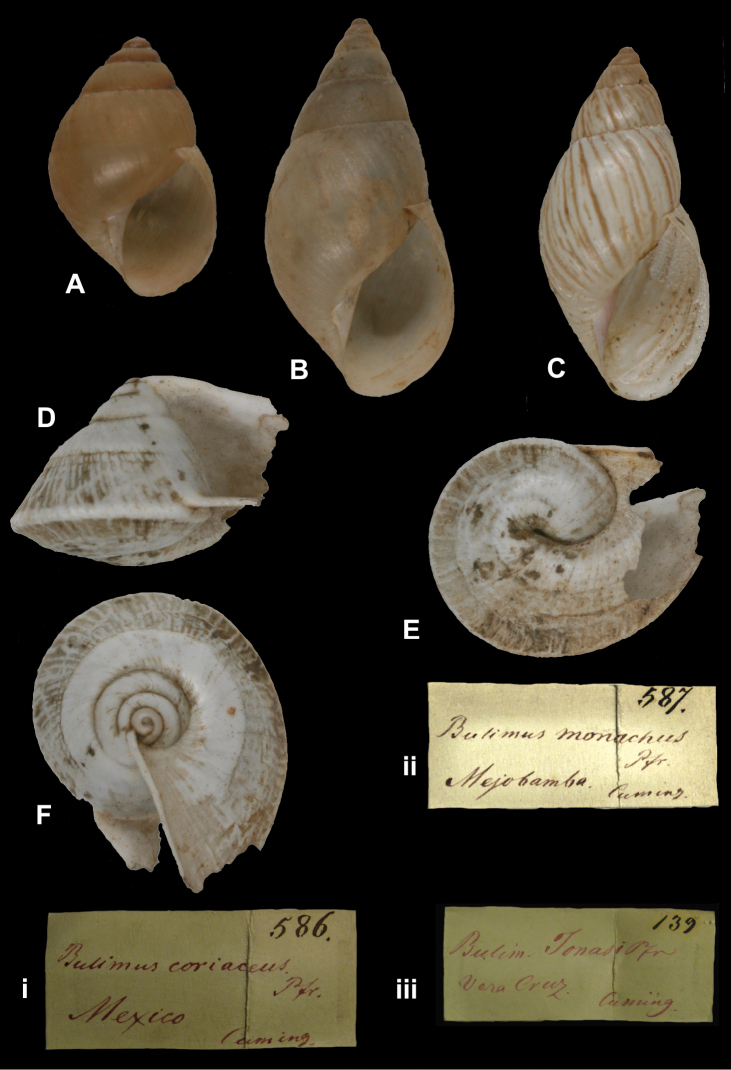
**A–B** *Bulimulus* species **A, i** *Bulimulus coriaceus* (Pfeiffer, 1857) syntype ZMB 117767 (H = 12.9) **B, ii** *Bulimulus monachus* (Pfeiffer, 1857) syntype ZMB 117773 (H = 24.2) **C, iii** *Drymaeus (Mesembrinus) jonasi* (Pfeiffer, 1846) syntype ZMB 117769 (H = 21.5) **D–F** *Cochlorina involuta* (Martens, 1867), holotype ZMB 117768 (H = 15.2). All enlarged.

**Figure 17.**
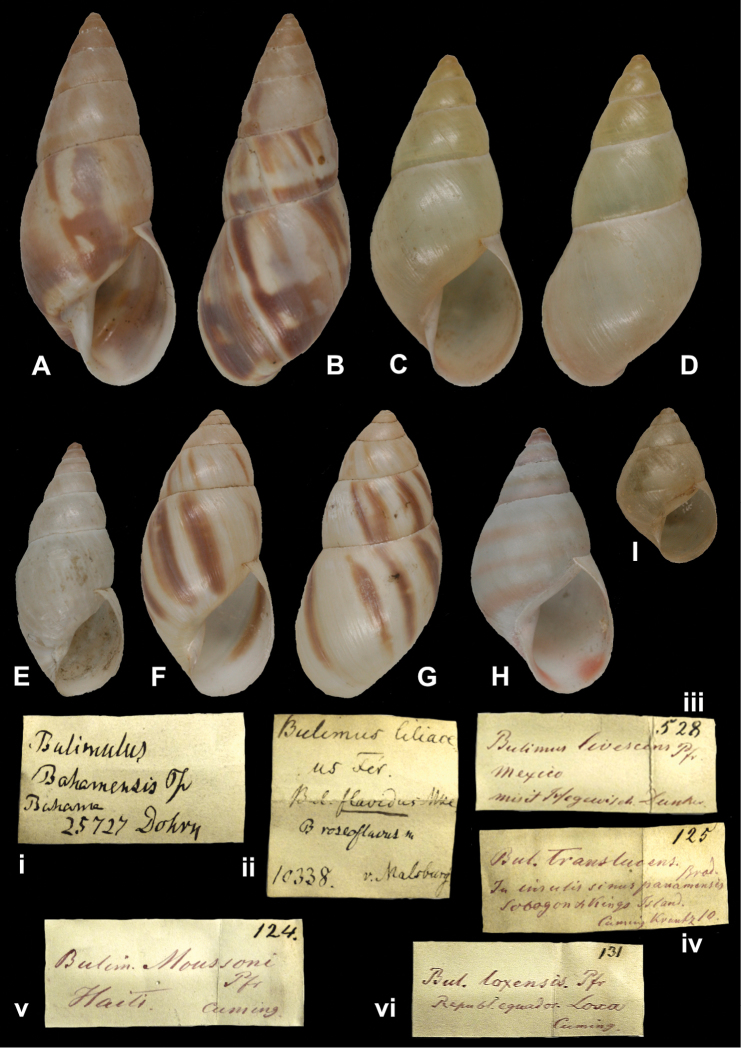
*Drymaeus (Mesembrinus)* species **A–B, i** *Drymaeus (Mesembrinus) bahamensis* (Pfeiffer, 1862), syntype ZMB 25727 (H = 29.4) **C–D, ii** *Drymaeus (Mesembrinus) flavidus* (Menke, 1829) probable syntype ZMB 10338 (H = 23.4) **E, iii** *Drymaeus (Mesembrinus) livescens* (Pfeiffer, 1842), probable syntype ZMB 117771 (H = 22.5) **F–G, vi** *Drymaeus (Mesembrinus) loxensis* (Pfeiffer, 1846), syntype ZMB 117772 (H = 24.7) **H, v** *Drymaeus (Mesembrinus) moussoni* (Pfeiffer, 1853), syntype ZMB 117774 (H = 23.5) **I, iv** *Drymaeus (Mesembrinus) translucens* (Broderip in Broderip and Sowerby I, 1832), probable syntype ZMB 117778 (H = 13.3).

**Figure 18.**
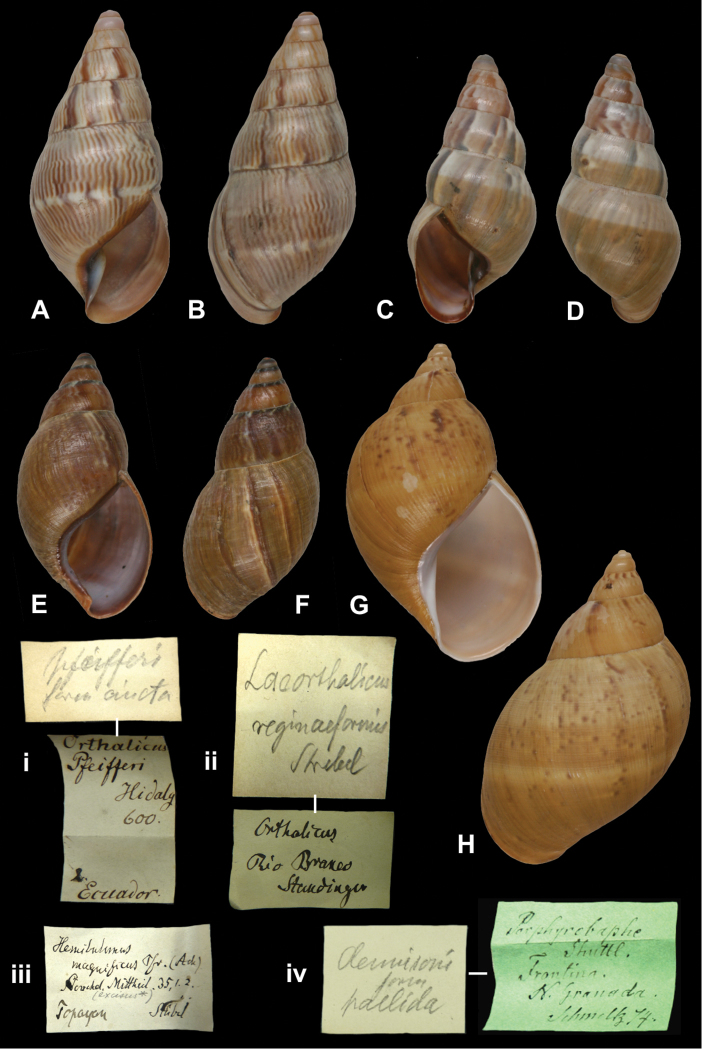
**A–D** *Corona* species **A–B, i** *Corona pfeifferi* (Hidalgo, 1869), syntype of *Corona pfeifferi cincta* Strebel, 1909 ZMB 101856 (H = 55.0) **C–D, ii** *Corona perversa* (Swainson, 1821), holotype of *Orthalicus (Laeorthalicus) reginaeformis* Strebel, 1909 ZMB 101824 (H = 46.9) **E–H** *Hemibulimus* species **E–F, iii** *Hemibulimus excisus* (Martens, 1885), holotype ZMB 101837 (H = 43.1) **G–H, iv** *Hemibulimus dennisoni* (Reeve, 1848), probable syntype of *Porphyrobaphe (Myiorthalicus) dennisoni pallida* Strebel, 1909 ZMB 117782 (H = 55.0).

**Figure 19.**
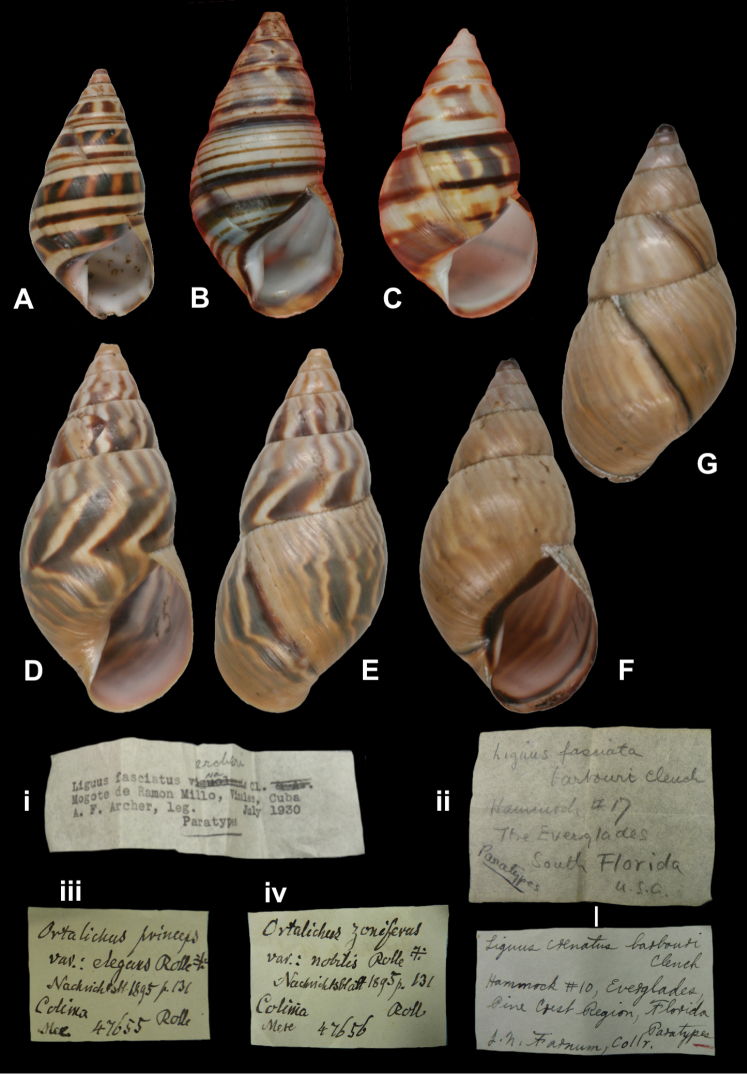
**A–C** *Liguus fasciatus* (Müller, 1774) **A, v** Probable syntype of *Achatina blainiana* Poey, 1853 ZMB 117781 (H = 39.0) **B, i** Paratype of *Liguus fasciatus archeri* Clench, 1934 ZMB 78796 (H = 51.9) **C, ii** Paratype of *Liguus crenatus barbouri* Clench, 1929 ZMB 74876 (H = 46.3) **D–E, iii** *Orthalicus elegans* Rolle, 1895, lectotype ZMB 47655 (H = 61.2) **F–G, iv** *Orthalicus nobilis* Rolle, 1895, lectotype ZMB 47656 (H = 58.8).

**Figure 20.**
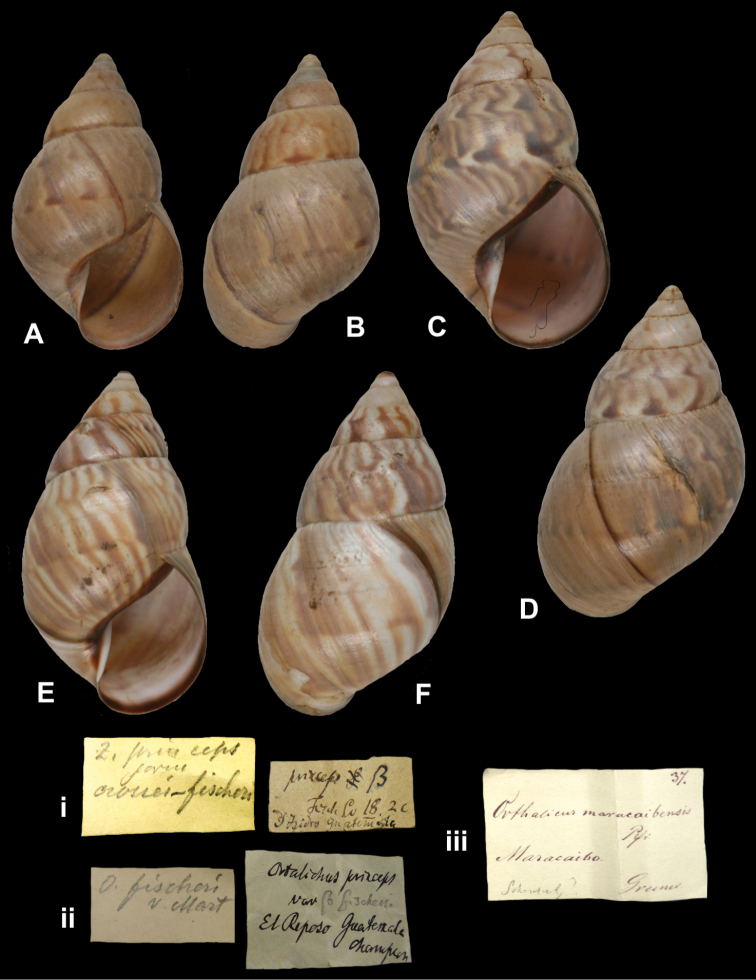
*Orthalicus* species **A–D** *Orthalicus princeps fischeri* Martens, 1893 **A–B, i** Syntype ZMB 109951 ( = holotype of *Zebra crosseifischeri* Strebel, 1909) (H = 50.2) **C–D, ii** Syntype ZMB 109950 (H = 60.0) **E–F, iii** *Orthalicus gruneri* (Strebel, 1909), holotype ZMB 117783 (H = 57.4).

**Figure 21.**
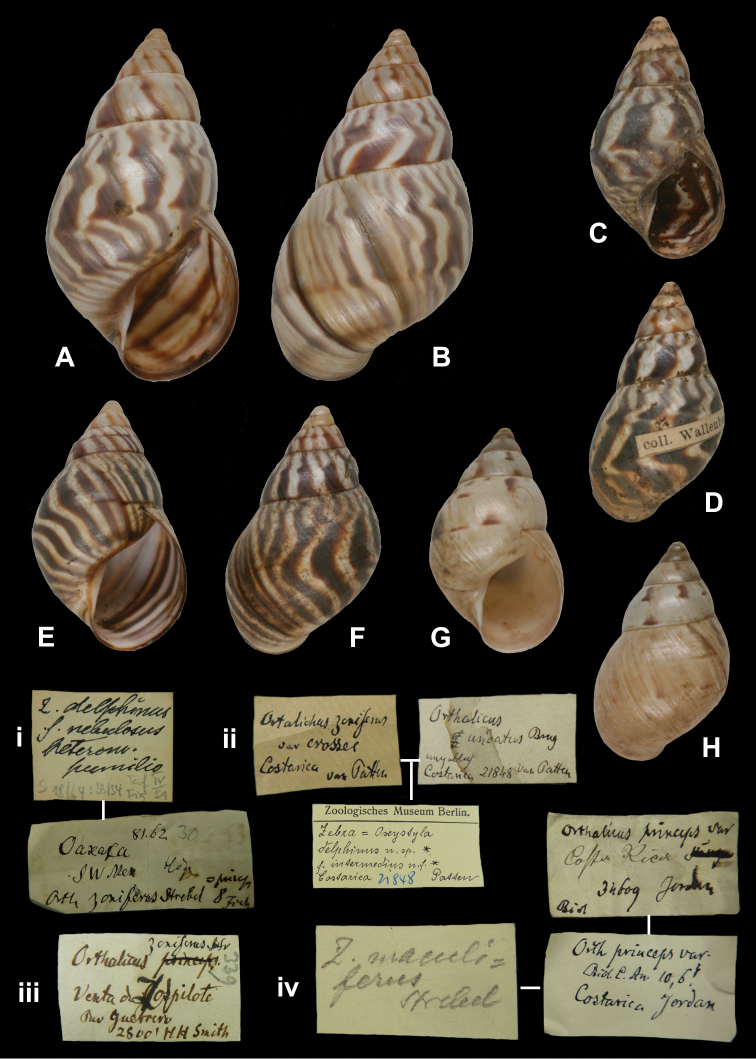
*Orthalicus* species **A–D** *Orthalicus delphinus* (Strebel, 1909) **A–B, ii** Holotype of *Zebra delphinus intermedius* Strebel, 1909 ZMB 21848 (H = 57.4) **C–D, i** Holotype of *Zebra delphinus pumilio* Strebel, 1909 ZMB 101834 (H = 39.3) **E–F, iii** *Orthalicus zoniferus* Strebel and Pfeffer, 1882, syntype of *Zebra zoniferus euchrous* Strebel, 1909 ZMB 28001 (H = 41.6) **G–H, iv** *Orthalicus maculiferus* (Strebel, 1909), holotype ZMB 34609 (H = 38.7).

**Figure 22.**
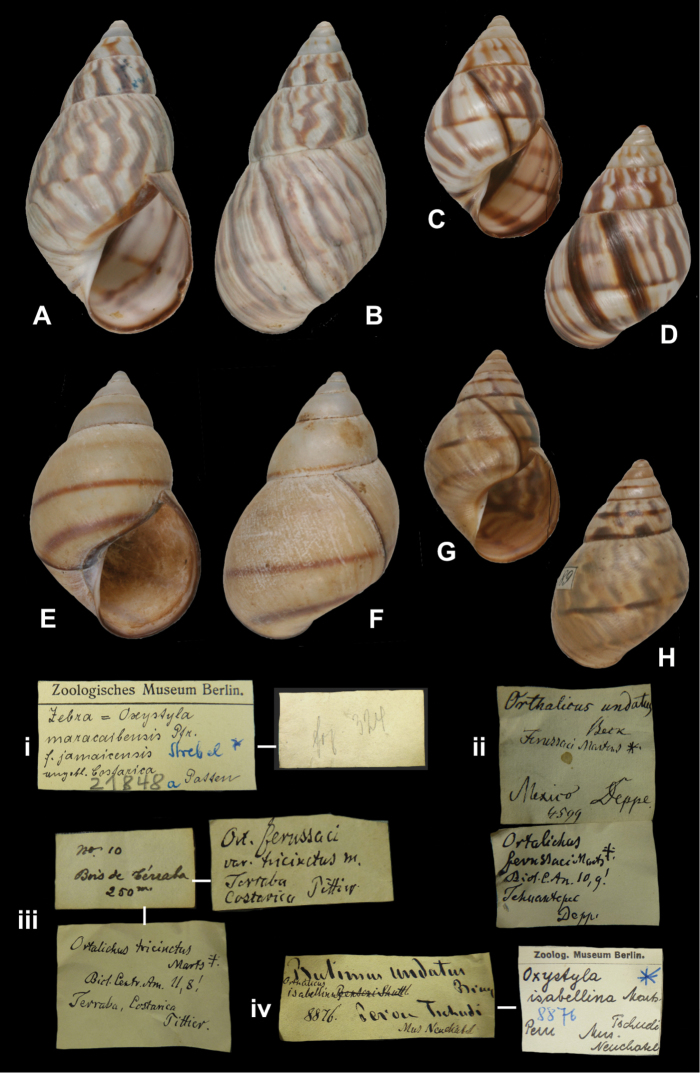
*Orthalicus* species **A–B, i** *Orthalicus maracaibensis* (Pfeiffer, 1856), syntype of *Zebra maracaibensis jamaicensis* Strebel, 1909 ZMB 21848a (H = 54.9) **C–D, ii** *Orthalicus ferussaci ferrusaci* Martens, 1864, syntype ZMB 4599 (H = 39.5) **E–F, iii** *Orthalicus ferussaci tricinctus* Martens, 1893, lectotype ZMB 101828 (H = 48.1) **G–H, iv** *Orthalicus bensoni* (Reeve, 1849), syntype of *Orthalicus isabellinus* Martens, 1873 ZMB 8876 (H = 37.0).

**Figure 23.**
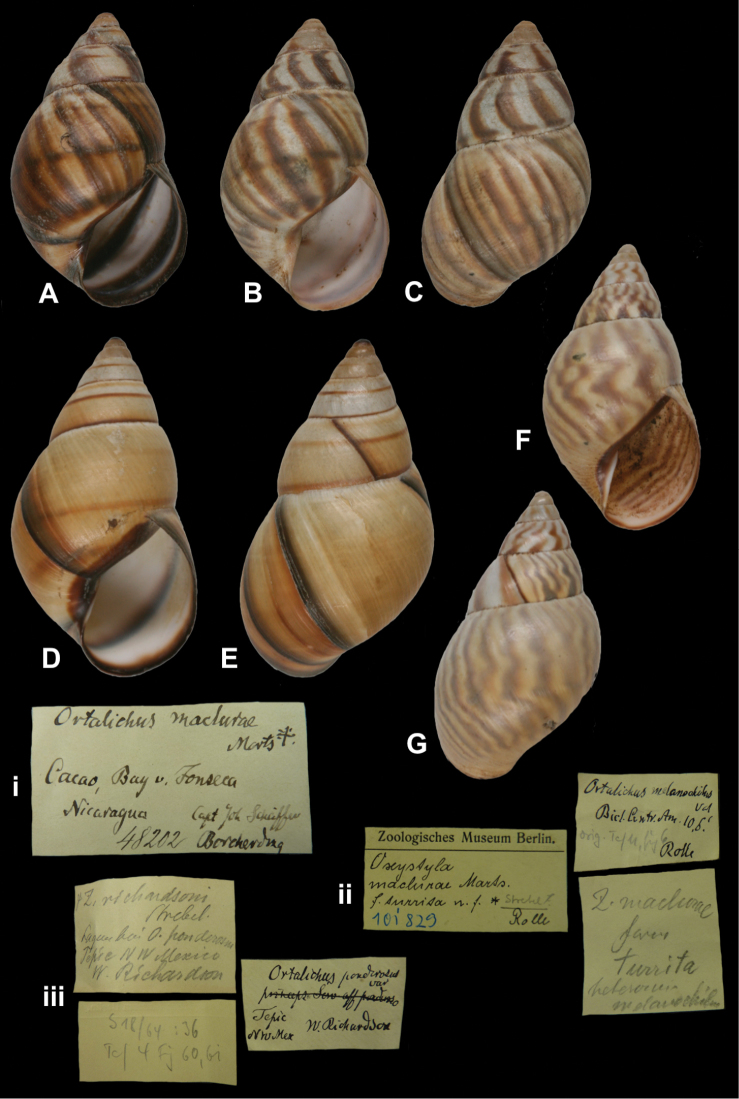
*Orthalicus* species **A–E** *Orthalicus maclurae* Martens, 1893 **A–C, i** Syntypes (**A** ZMB 48202a “var. b”, **B–C** ZMB 48202b) (H = **A** 49.0, **B–C** 48.5) **D–E, ii** Syntype of *Zebra maclurae turrita* Strebel, 1909 ZMB 101829 (H = 56.2) **F–G, iii** *Orthalicus richardsoni* (Strebel, 1909), syntype ZMB 101831 (H = 47.1).

**Figure 24.**
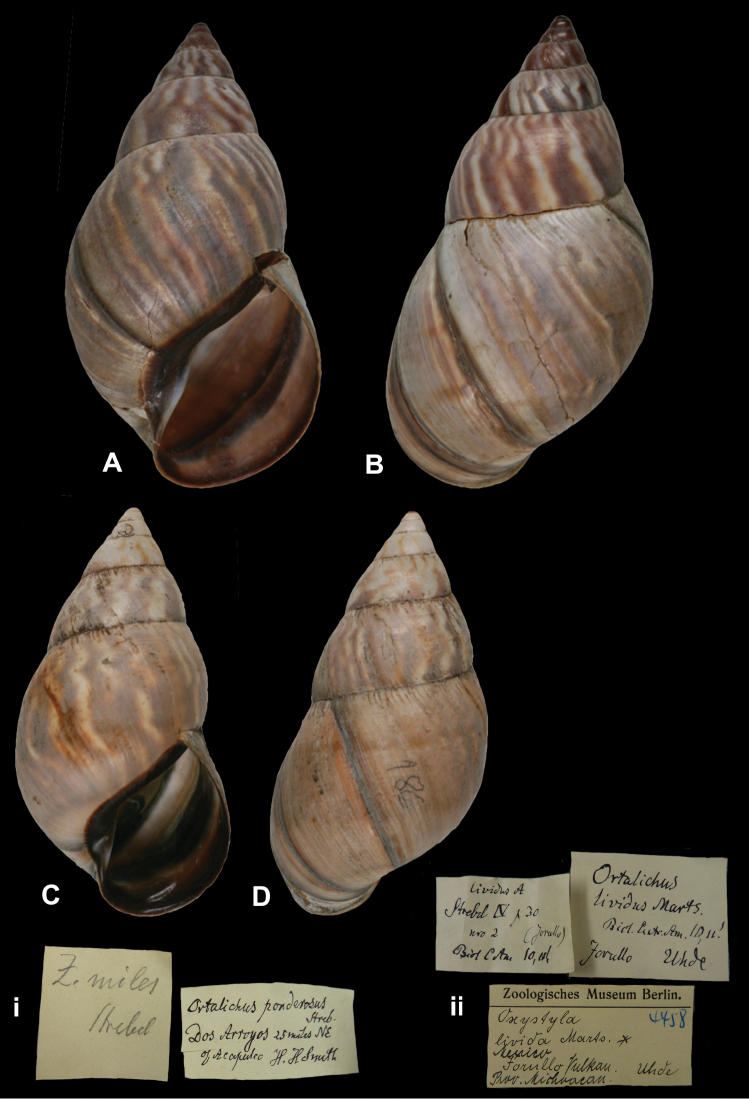
*Orthalicus* species **A–B, ii** *Orthalicus lividus* Martens, 1864, syntype ZMB 4458 (H = 79.4) **C–D, i** *Orthalicus ponderosus ponderosus* Strebel in Strebel and Pfeffer, 1882, syntype of *Zebra miles* Strebel, 1909 ZMB 101830 (H = 71.0).

**Figure 25.**
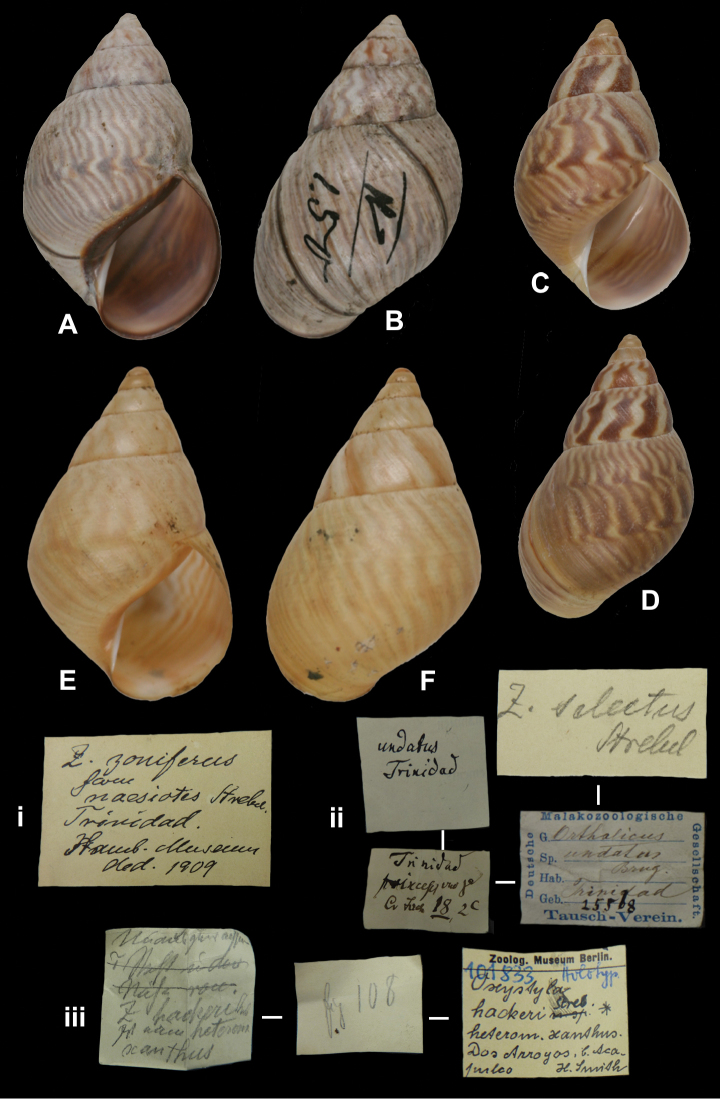
*Orthalicus* species **A–D** *Orthalicus undatus* (Bruguière, 1789) **A–B, i** Syntype of *Zebra zoniferus naesiotes* Strebel, 1909 ZMB 117785 (H = 53.4) **C–D, ii** Syntype of *Zebra selectus* Strebel, 1909 (H = 50.3) **E–F, iii** *Orthalicus hackeri* (Strebel, 1909), holotype of *Zebra hackeri xanthus* Strebel, 1909 ZMB 101833 (H = 57.4).

**Figure 26.**
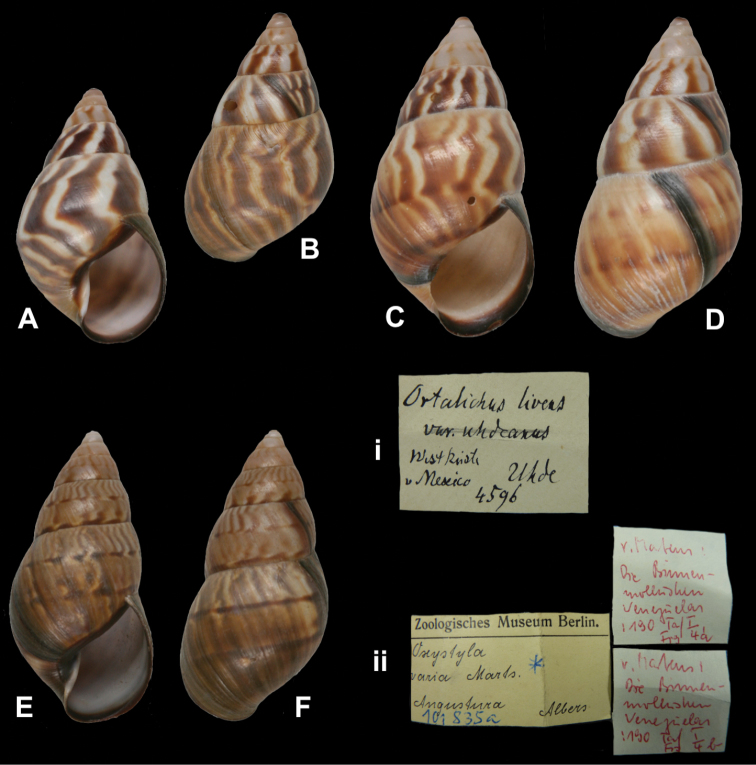
*Orthalicus* species **A–D** *Orthalicus uhdeanus* Martens, 1893 **A–B, i** syntype ZMB 4995 (H = 39.3) **C–D** syntype ZMB 4996 (H = 54.3) **E–F, ii** *Orthalicus varius* Martens, 1873, syntype ZMB 101835a (H = 47.2).

**Figure 27.**
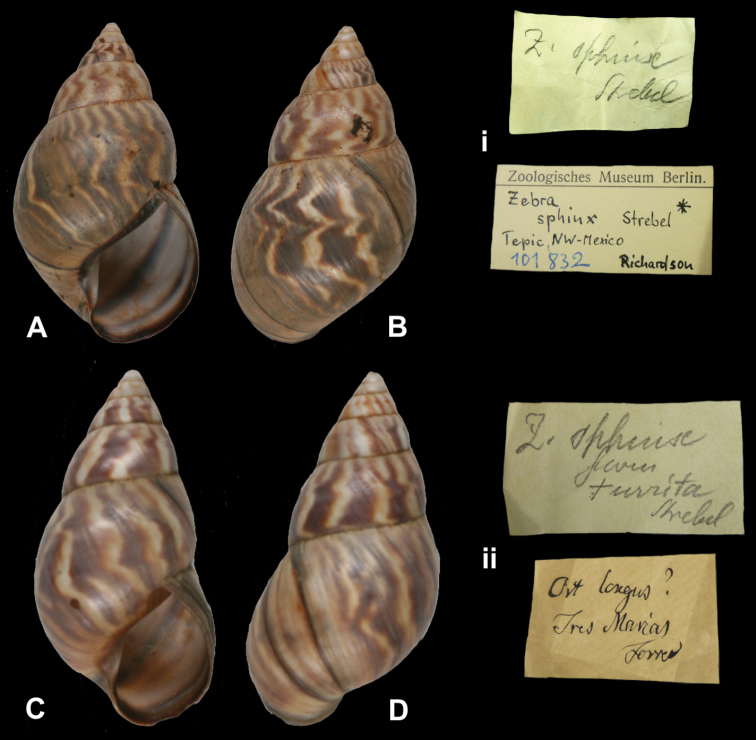
*Orthalicus* species **A–B, i** *Orthalicus sphinx sphinx* (Strebel, 1909), syntype ZMB 101832 (H = 50.9) **C–D, ii** *Orthalicus sphinx tresmariae* Breure nom. n., holotype ZMB 117784 (H = 56.9).

**Figure 28.**
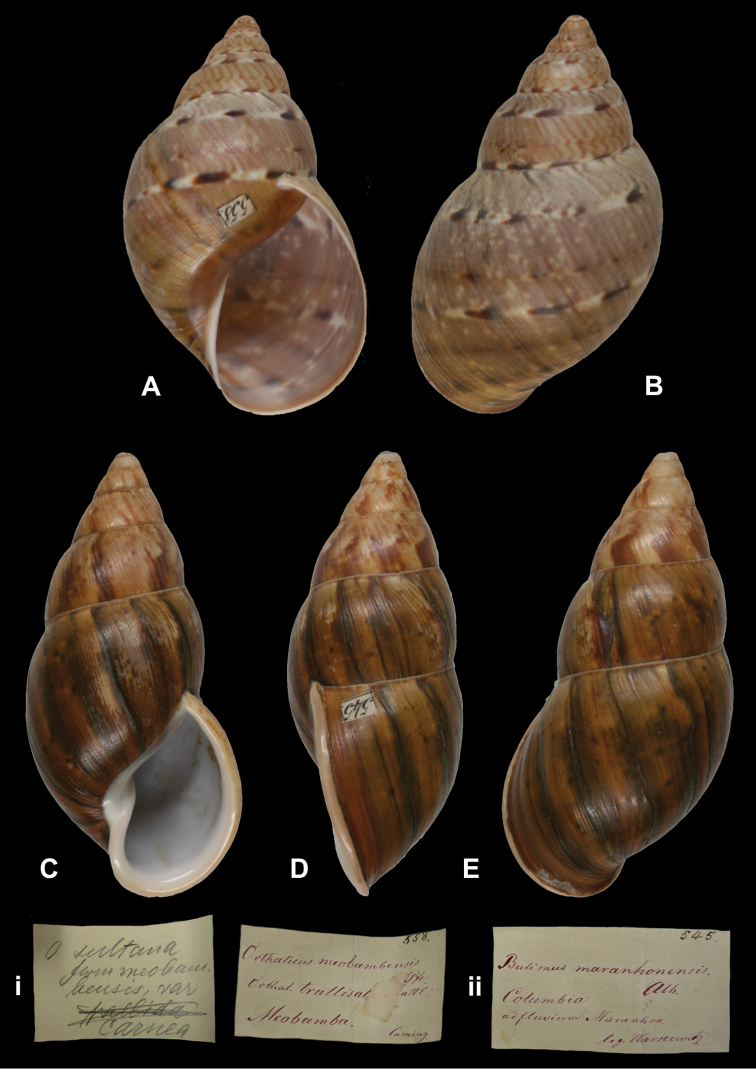
*Sultana (Metorthalicus)* species. **A–B, i** *Sultana (Metorthalicus) meobambensis* (Pfeiffer, 1855), holotype of *Orthalicus meobambensis carnea* Strebel, 1909 ZMB 101823 (H = 68.7) **C–E, ii** *Sultana (Metorthalicus) maranhonensis* (Albers, 1854), lectotype ZMB 101825 (H = 75.6).

**Figure 29.**
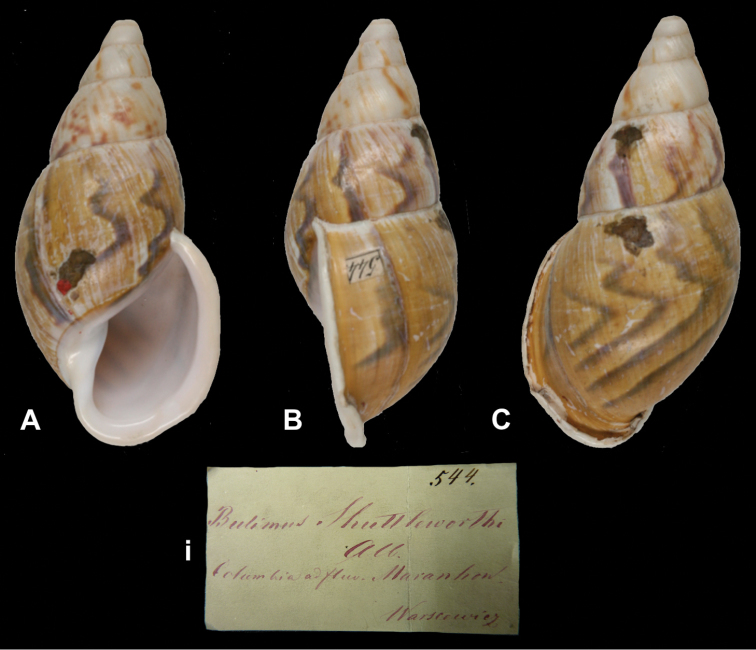
**A–B, i** *Sultana (Metorthalicus) shuttleworthi* (Albers, 1854), syntype ZMB 101827 (H = 70.3).

**Figure 30.**
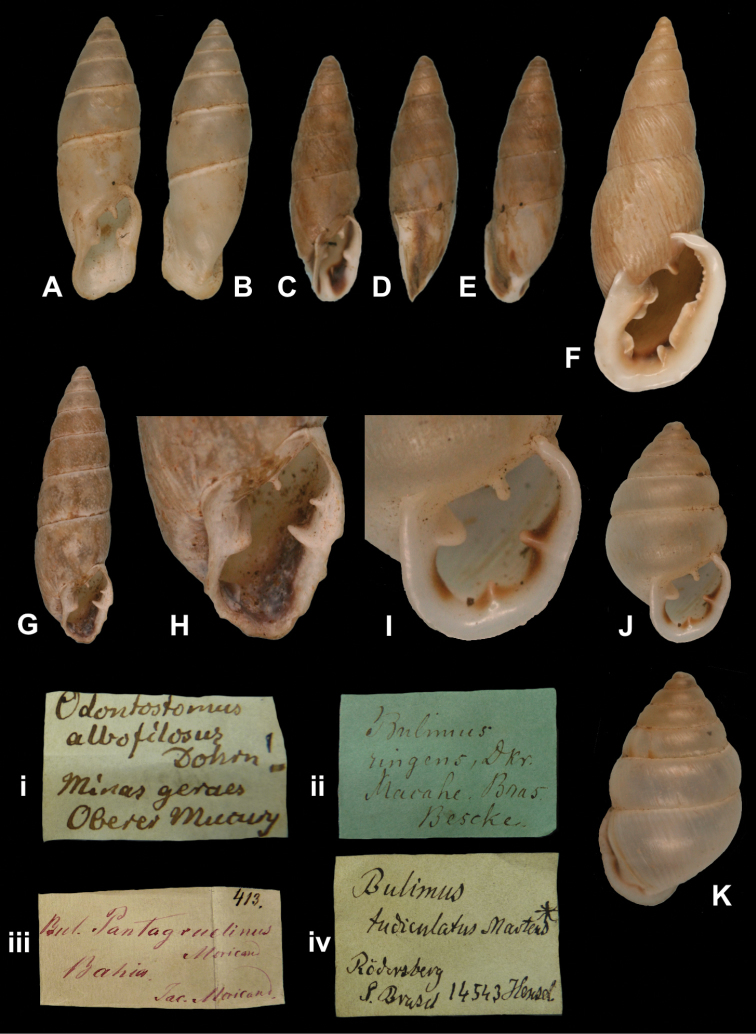
**A–B, i** *Bahiensis albofilosus* (Dohrn, 1863), syntype ZMB 36424 (H = 22.5) **C–E, ii** *Bahiensis ringens* (Dunker in Dunker et al., 1847), syntype ZMB 117780 (H = 18.6) **F, iii** *Burringtonia pantagruelina* (Moricand, 1833), syntype ZMB 117779 (H = 54.3) **G–H, iv** *Cyclodontina tudiculata* (Martens, 1868), syntype ZMB 14543 (H = 21.5) **I–K** *Cyclodontina trahyrae* (Jaeckel, 1950), holotype ZMB 95737 (H = 12.16). All enlarged.

**Figure 31.**
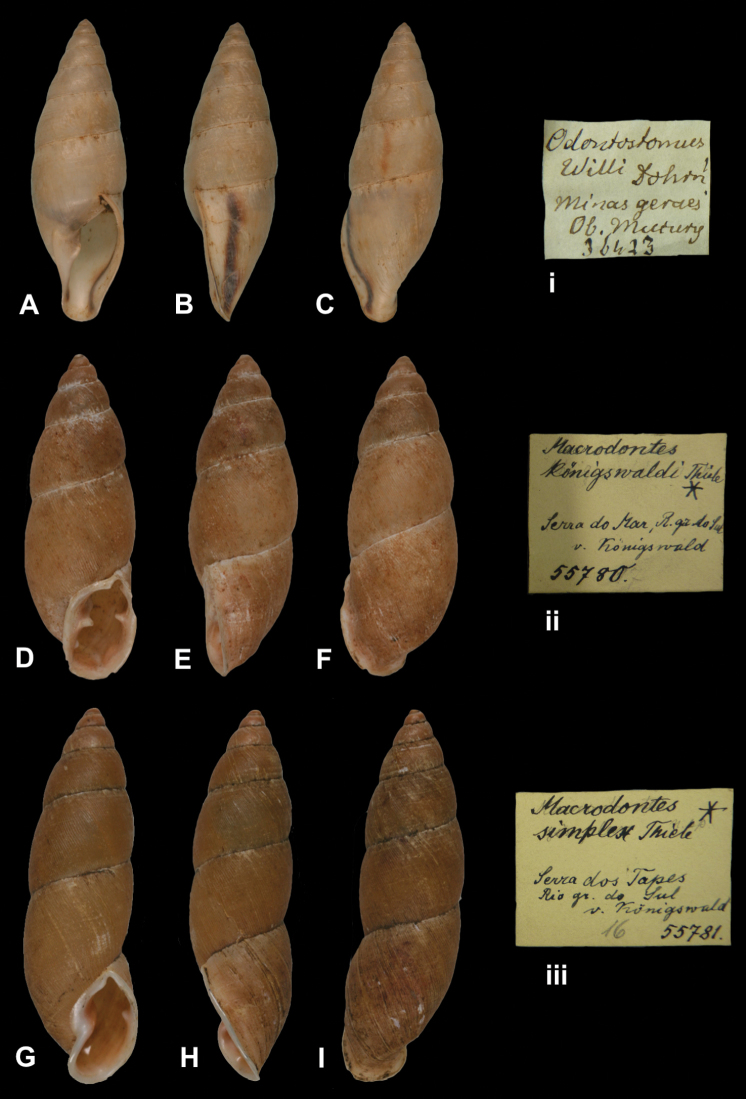
**A–C, i** *Moricandia willi* (Dohrn, 1883), syntype ZMB 36423 (H = 27.9) **D–F, ii** *Odontostomus koenigswaldi* (Thiele, 1906), holotype ZMB 55780 (H = 30.5) **G–I, iii** *Odontostomus simplex* (Thiele, 1906), holotype ZMB 55781 (H = 34.9). All enlarged.

**Figure 32.**
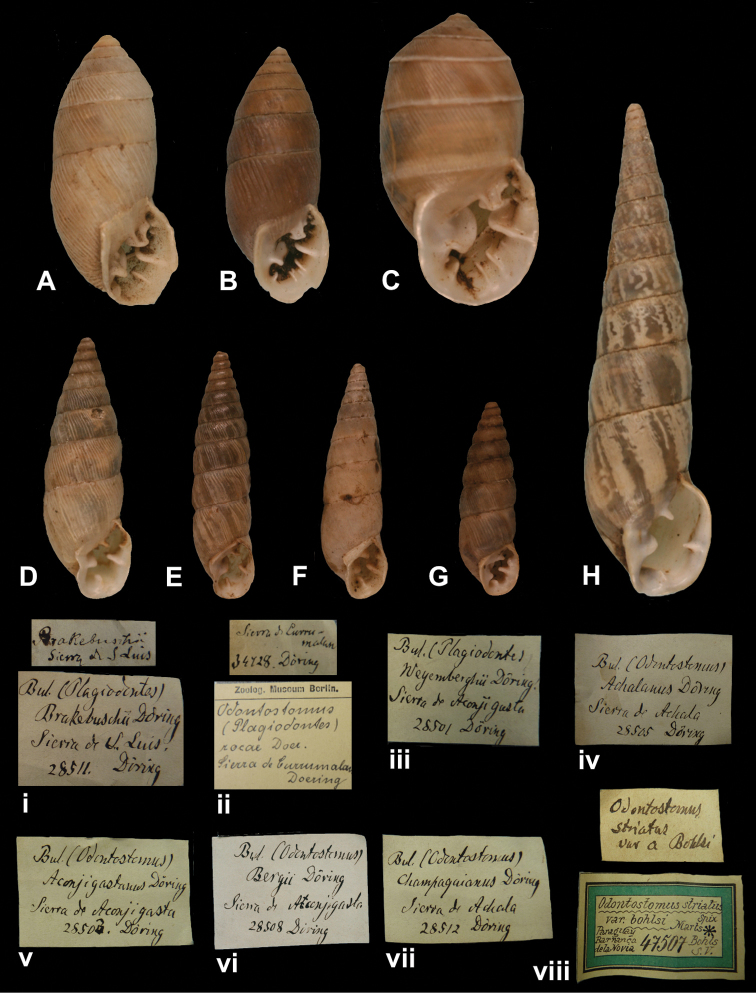
**A–C** *Plagiodontes* species **A, i** *Plagiodontes brackebuschii* (Doering, 1877), paralectotype ZMB 28511 (H = 25.1) **B, ii** *Plagiodontes rocae* Doering, 1881, syntype ZMB 34728 (H = 23.7) **C, iii** *Plagiodontes weyenberghii* (Doering, 1877), paralectotype ZMB 28501 (H = 26.6) **D–H** *Spixia* species **D, iv** *Spixia achalanus* (Doering, 1877), paralectotype ZMB 28505 (H = 21.7) **E, v** *Spixia aconjigastanus* (Doering, 1877), paralectotype ZMB 28503 (H = 20.0) **F, vi** *Spixia bergii* (Doering, 1877), paralectotype ZMB 28508 (H = 19.2) **G, vii** *Spixia champaquianus* (Doering, 1877), paralectotype ZMB 28512 (H = 14.1) **H, viii** *Spixia striata* (Spix, 1827), syntype of *Odontostomus striatus bohlsi* Martens, 1894 (H = 50.2) All enlarged.

**Figure 33.**
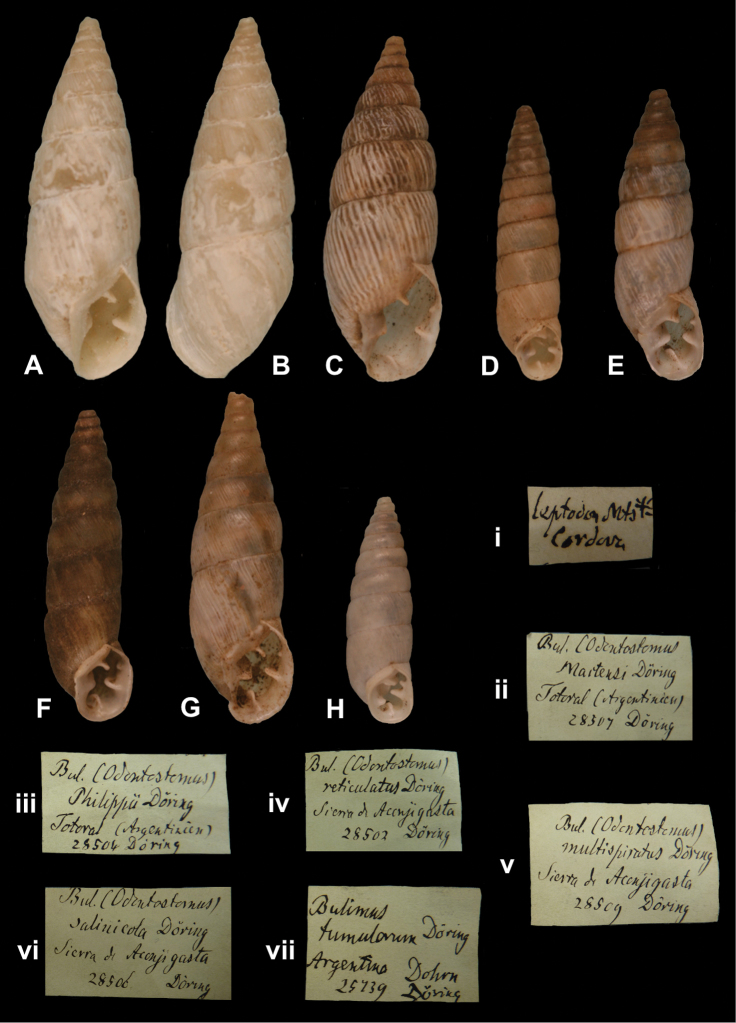
*Spixia* species **A–B, i** *Spixia alvarezii* (d’Orbigny, 1837), syntype of *Bulimus (Odontostomus) leptodon* Martens, 1875 ZMB 24077 (H = 22.2) **C, ii** *Spixia martensii* (Doering, 1875), paralectotype ZMB 28807 (H = 21.4) **D, v** *Spixia multispirata* (Doering, 1877), paralectotype ZMB 28509 (H = 17.4) **E, iii** *Spixia philippii* (Doering, 1875), syntype ZMB 28504 (H = 18.0) **F, iv** *Spixia reticulata* (Doering, 1877), paralectotype ZMB 28502 (H = 18.8) **G, vi** *Spixia salinicola* (Doering, 1877), paralectotype ZMB 28506 (H = 20.4) **H, vii** *Spixia tumulorum* (Doering, 1875), paralectotype ZMB 25739 (H = 13.6). All enlarged.

## Figures and Tables

**Table 1.** Number of types of Orthalicoidea per family in the ZMB collection.

**Status**	**Amphibulimidae**	**Bothriembryontidae**	**Bulimulidae**	**Megaspiridae**	**Odontostomidae**	**Orthali-cidae**	**Simpulopsidae**	**Orthalicoidea**
Holotypes	1	2	5		3	9		20
Paratypes		7	6			5		18
Lectotypes	6	2	45			4		57
Paralectotypes	3	3	9		11	2		28
Syntypes	2	9	37	1	7	18	6	80
Possible / probable types	3	1	23	1	2	5		35
Total	15	24	125	2	23	43	6	238

## Appendix

### List of all taxa for which Orthalicodea types are extant, or discussed, in the ZMB collection

**Remarks.** This list has been compiled both from Köhler (2007)—taxa indicated by K followed by the page number therein—and from this paper.

*achalanus* Doering, 1877—8

*achatellinus* Forbes, 1850—K136

*achrous* Haas, 1952—K141

*aconjigastanus* Doering, 1877—9

*acutus* Reibisch, 1892—K136

*albemarlensis* Dall, 1917—K136

*albida* da Costa, 1907—K143

*albidus* Martens, 1893—K148

*albivaricosus* Martens, 1893—K143

*albofilosus* Dohrn, 1883—9

*albus* Sowerby I, 1833—K131

*anachoraeta* Pfeiffer, 1856—K131

*anceps* Albers, 1854—K150

*angosturensis* Gruner, 1841—K154

*appunii* Dunker, 1875—K127

*archeri* Clench, 1934—10

*atacamensis* Pfeiffer, 1856—K131

*aukiensis* Clapp, 1923—10

*aulacostylus* Pfeiffer, 1853—11

*bahamensis* Pfeiffer, 1862—11

*barbouri* Clench, 1929—12

*baroni* Fulton, 1896—K140

*bergii* Doering, 1877—12

*bilineatus* Sowerby I, 1833—K131

*blainianus* Poey, 1853—13

*blanchetiana* Moricand, 1833—K143

*bolshi* Martens, 1894—13

*bonneti* Ancey, 1902—14, K131

*borealis* Martens, 1893—K150

*brackebuschi* Doering, 1877—14

*brasiliensis* Moricand, 1846—K156

*broderipii* Sowerby I, 1832—K141

*camachoi* Weyrauch, 1967—K143

*canaliferus* Reibisch, 1892—K137

*carandaitiensis* Preston, 1907—K143

*cardinalis* Pfeiffer, 1853—K127

*carnea* Strebel, 1909—15

*catlowiae* Pfeiffer, 1853—K137

*centralis* Doering, 1879—K137

*chacoensis* Preston, 1907—K132

*champaquianus* Doering, 1877—15

*chanchamayensis* Hidalgo, 1870—K144

*chemnitzioides* Forbes, 1850—K137

*chiletensis* Weyrauch, 1967—K140

*chrysomelas* Martens, 1867—K144

*cincta* Strebel, 1909—16

*colimensis* Rolle, 1895—K144

*concolor (attenuatus)* Martens, 1893—K145

*concolor (josephus)* Martens, 1893—K145

*conospirus* Doering, 1879—16

*coquimbensis* Broderip, 1832—K141

*cordillerae* Strobel, 1874—K132

*coriaceus* Pfeiffer, 1857—16

*coturnix* Sowerby I, 1832—K142

*coxeirana* Moricand, 1836—K154

*crenulatus* Weyrauch, 1958—17

*crosseifischeri* Strebel, 1909—17

*edmuelleri* Albers, 1854—K145

*elegans* Rolle, 1895—18

*elegantulus* Weyrauch, 1956—K137

*erosus* Broderip, 1832—K132

*erythrostoma* Sowerby I, 1833—K132

*euchrous* Strebel, 1909—18

*excisus* Martens, 1885—19

*eximius* Albers, 1857—19

*fenestrellus* Martens, 1864—K145

*ferussaci* Martens, 1864—20

*fischeri* Martens, 1893—20

*flavidus* Menke, 1829—##

*fuscus* Thiele, 1930—K142

*gayi* Pfeiffer, 1857—K141

*glaucostomus* Albers, 1852—K145

*gracilior* Martens, 1893—K150

*gruneri* Strebel, 1909—21

*guerini* Pfeiffer, 1846—K128

*guildingi* Dohrn, 1875—K127

*henselii* Martens, 1868—K145

*hepaticus* Albers, 1854—K146

*heterotricha* Moricand, 1836—K154

*hiabundus* Martens, 1893—K146

*hypozonus* Martens, 1893—K150

*ictericus (depictus)* Martens, 1873—K150

*ictericus (liliacinus)* Martens, 1893—K146

*icterostomus* Martens, 1901—K146

*infundibulum* Pfeiffer, 1853—K132

*insignis* Petit, 1850—22

*intermedius* Strebel, 1909—22

*interpictus* Martens, 1867—K146

*interpunctus* Martens, 1886—K150

*interruptus* Preston, 1909—K151

*interstitialis* Martens, 1893—K151

*inusitatus* Fulton, 1900—K151

*involutus* Martens, 1867—23

*isabellinus* Martens, 1873—23

*jacobi* Sowerby I, 1833—K137

*jamaicensis* Strebel, 1909—24

*jansoni* Martens, 1893—K146

*jonasi* Pfeiffer, 1846—24

*koenigswaldi* Thiele, 1906—25

*lachayensis* Weyrauch, 1967—K140

*largillierti* Philippi, 1845—K129

*latior* Strebel, 1882—K146

*laureani* Pratt, 1949—25

*laurentii* Sowerby I, 1833—25

*leptodon* Martens, 1875—26

*leucomelas* Albers, 1854—K151

*leucostictus* Philippi, 1856—K132

*lineolatus* Martens, 1893—K151

*livescens* Pfeiffer, 1842—26

*lividus* Martens, 1864—27

*loxensis* Pfeiffer, 1846—27

*lugubris* Dunker, 1882—28

*macgillivrayi* Pfeiffer, 1856—28

*maclurae* Martens, 1893—29

*maculiferus* Strebel, 1909—29

*major* Martens, 1897—K134

*manni* Clapp, 1923—30

*manugiensis* B. Rensch, 1934—30

*maranhoensis* Albers, 1854—31

*mariae* Albers, 1850—K140

*martensii* Doering, 1875—31

*martensi* Kobelt, 1901—K143

*martensianus* Pilsbry, 1899—K151

*melanoscolops* Dohrn, 1882—K147

*mendozanus* Strobel, 1874—K135

*menkei* Gruner, 1841—K151

*midas* Albers, 1852—K128

*miles* Strebel, 1909—32

*millegranus* Martens, 1885—K143

*miltochrous* Albers, 1854—K152

*minor* Pfeiffer, 1847—K129

*moestai* Dunker, 1864—K142

*monachus* Pfeiffer, 1857—32

*montaguae* Martens, 1893—K152

*monticola* Doering, 1879—33

*morenoi* Preston, 1907—K152

*moritinctus* Martens, 1893—K152

*moritzianus* Pfeiffer, 1847—K129

*moussoni* Pfeiffer, 1853—33

*muelleggeri* Jaeckel, 1927—K147

*multispiratus* Doering, 1877—34

*mutabilis* Broderip, 1832—K140

*naesiotes* Strebel, 1909—34

*nanus* Reeve, 1849—K133

*narcissus* Albers, 1854—K147

*nasutus* Martens, 1885—35

*nehringi* Martens, 1889—K131

*nigrogularis* Dohrn, 1882—K147

*nobilis* Rolle, 1895—35

*nuciformis* Petit, 1853—K137

*nux* Broderip, 1832—K138

*nystianus* Pfeiffer, 1853—K147

*occidentalis* Weyrauch, 1960—K129

*ochsenii* Dunker, 1855—K142

*ortizpuenti* Weyrauch, 1967—36

*paeteli* Albers, 1854—K152

*paeteli* Kobelt, 1890—36

*pallida* Strebel, 1909—36

*pallidus* Reibisch, 1892—K138

*pantagruelina* Moricand, 1833—37

*paposensis* Pfeiffer, 1856—K133

*paravicinianus* B. Rensch, 1934—37

*paravicinii* B. Rensch, 1934—38

*perdix* Pfeiffer, 1848—K128

*peristomatus* Doering, 1879—38

*phenax* Clapp, 1923—39

*philippii* Doering, 1875—39

*piescheli* Martens, 1863—K152

*pinchoti* Pilsbry, 1930—K152

*piperitus* Sowerby I, 1833—K127

*pittieri* Martens, 1893—K147

*piuranus* Albers, 1854—K133

*porphyrius* Pfeiffer, 1847—K129

*porrectus* Martens, 1893—K153

*proteus* Broderip, 1832—K141

*psidii* Martens, 1877—40, K156

*pumilio* Strebel, 1909—40

*punctatus* da Costa, 1907—K148

*punctulifer* Sowerby I, 1833—K142

*pupiformis* Broderip, 1832—K133

*pustulosus* Broderip, 1832—K133

*reflexus* Pfeiffer, 1842—K142

*reginaeformis* Strebel, 1909—40

*regularis* Fulton, 1905—K148

*reticulatus* Doering, 1879—41

*rhodacme* Pfeiffer, 1842—42

*rhodolarynx* Reeve, 1849—42

*rhodotrema* Martens, 1868—K153

*richardsoni* Strebel, 1909—43

*riisei* Pfeiffer, 1855—K135

*ringens* Dunker, 1847—43

*rocae* Doering, 1881—44

*rugiferus* Sowerby I, 1833—K138

*sachsei* Albers, 1854—K148

*salinicola* Doering, 1877—44

*sarcodes* Pfeiffer, 1846—K135

*scabiosus* Sowerby I, 1833—K134

*scarabus* Albers, 1854—44

*selectus* Strebel, 1909—45

*sepulcralis* Poey, 1853—K136

*shuttleworthi* Albers, 1854—45

*simplex* Thiele, 1906—46

*sinuatus* Albers, 1854—K128

*solidus* Preston, 1907—K148

*solutus* Troschel, 1847—K134

*spectrum* Albers, 1854—K128

*sphinx* Strebel, 1909—46

*stelzneri* Dohrn, 1875—47

*strangei* Pfeiffer, 1855—47

*stutchburyi* Pfeiffer, 1860—48

*stuebeli* Martens, 1885—49, K129

*subunicolor* Martens, 1893—K153

*taylorianu*s Reeve, 1849—K127

*terebralis* Pfeiffer, 1842—49

*tetensii* Dunker, 1875—K127

*tortoranus* Doering, 1879—50

*trahyrae* Jaeckel, 1950—50

*translucens* Broderip, 1832—51

*tresmariae* Breure nom. n.—54

*tricinctus* Martens, 1893—51

*trimarianus* Martens, 1893—K153

*tschudii* Pfeiffer, 1848—K134

*tudiculatus* Martens, 1868—52

*tumulorum* Doering, 1875—52

*turrita, maclurae* Strebel, 1909—53

*turrita, sphinx* Strebel, 1909—53

*turritus* Broderip, 1832—K134

*uhdeanus (Bulimulus)* Martens, 1863—K153

*uhdeanus (Orthalicus)* Martens, 1893—54

*uliginosus* Kobelt, 1890—54

*undulosus* Martens, 1893—K148

*unicolor* Martens, 1893—K153

*unicolor* Sowerby I, 1833—K136

*ustulatus* Sowerby I, 1833—K138

*varius* Martens, 1873—55

*velutinohispida* Moricand, 1836—K155

*ventrosus* Reibisch, 1892—K138

*versicolor* Broderip, 1832—K141

*vestalis* Albers, 1854—K139

*vicinus* Preston, 1907—K148

*vicinus* B. Rensch, 1934—55

*viminea* Moricand, 1833—K154

*vincentinus* Pfeiffer, 1846—K154

*weyenberghii* Doering, 1877—56

*willi* Dohrn, 1883—57

*wolfi* Reibisch, 1892—K140

*xantholeucus* Martens, 1893—K154

*xanthus* Strebel, 1909—57

### List of persons relevant for this study of which handwritten correspondence is present in the ZMB archive

**Remarks.** The following persons are known to have worked with orthalicoid taxa or contributed or collected material used for studies in this group. The letters in the Handwriting archive are not necessarily dealing with these studies. Persons without active contribution but of interest for the biohistory of Neotropical terrestrial malacology are marked with an asterisk (*). For a double asterisk (**), see also Breure and Ablett (2011: 4).

Albers, J.C.; Berlin—1850

Biolley, P.; San José—1891

Boettger, O.; Frankfurt a.M.—1874–1910

Bohls, J.; Hamburg—1894

Bonnet, A.; Paris—1909

Champion, C.; London—1890–1898

Charpentier, J. de; Devens near Bex—1852–1853

Cox, J.C.; Sydney—1869–1870

Cuming, H.; London—1864–1865

Dall, W.H.; Washington D.C.—1897–1905

Dautzenberg, Ph.; Paris—1928–1931

Dohrn, A.; Stettin / Berlin—1876, 1901

Dunker, W.; Cassel / Marburg—1901–1904

Ernst, A.; Caracas—1871

Eyerdam, W.; Seattle—1930

Fulton, H.C.; London—1909

Germain, L.; Paris—1929

Göldi, E.A.*; Rio de Janeiro—1888–1889

Gundlach, J.; Habana—1874–1881

Haas, F.; Heidelberg—1909

Hedley, C.; Sydney—1906

Hidalgo, J.G.; Madrid—1913

Ihering, H. von; São Paulo—1897–1898

Iredale, T.; London—1914

Kobelt, W.; Schwanheim—1892–1912

Martens, E. von; Berlin—1858–1899

Menke, K.Th.; Pyrmont—1843, 1853

Mousson, A.; Zürich—1860

Oberwimmer, A.; Mitterarnsdorf—1924

Odhner, N.; Stockholm—1929

Paetel, F.; Berlin—1855

Paravicini, E.; Basel—1931

Pfeffer, G.; Hamburg—1896–1931

Pfeiffer, L.; Kassel—1841–1868

Pilsbry, H.A.; Kansas—1910?

Pittier, H.; San José—1891, 1893

Preston, H.B.; London—1912

Reibisch, T.; Dresden—1853, 1855

Robson, G.C.**; London—1828, 1830

Rolle, H.; Berlin—1927

Salvin, O.*; London—1889–1897

Smith, E.A.; London—1897–1900?

Sowerby, G.B. III; London—1906

Strebel, H.; Hamburg—1874, 1877

Strobel, P.; Pavia—1871

Suter, H.; Christchurch—1897–1914

Thiele, J.; Dresden—1892

Troschel, H.; Bonn—1864–1871

Vernhout, J.H.; Leiden—1913

Wagner, A.*; Dremlach—1913

Wessel, C.; Hamburg—1874
